# Expanding Glycomic Investigations through Thiol-Derivatized Glycans

**DOI:** 10.3390/molecules28041956

**Published:** 2023-02-18

**Authors:** Robert D. Hurst, Angel Nieves, Matthew Brichacek

**Affiliations:** Department of Chemistry, University of Maine, Orono, ME 04469, USA

**Keywords:** glycans, thiols, reductive amination, photolithography, bioconjugation, covalent chromatography, neoglycoprotein

## Abstract

N-(2-thioethyl)-2-aminobenzamide (TEAB), a novel glycan auxiliary, was synthesized and its utility was evaluated. The auxiliary was conjugated to glycans by reductive amination with the water-stable reagent 2-picoline borane complex. Glycan products, which ranged from 1 to 7 linked hexoses, were all isolated in yields ranging from 60% to 90% after purification by reverse-phase chromatography. The novel conjugate introduces a convenient, shelf-stable thiol directly onto the desired free glycans with purification advantages and direct modification with efficient reactions through alkenes, halides, epoxides, disulfides, and carboxylates in yields of 49% to 93%. Subsequently, a thiol-selective modification of the BSA protein was used to generate a neoglycoprotein with a bifunctional PEG–maleimide linker. To further illustrate the utility of a thiol motif, 2-thiopyridine activation of a thiol-containing support facilitated the covalent chromatographic purification of labeled glycans in yields up to 63%. Finally, initial proof of concept of implementation in a light printed microarray was explored and validated through FITC-labeled concanavalin A binding. In conclusion, the thiol-functionalized glycans produced greatly expand the diversity of bioconjugation tools that can be developed with glycans and enable a variety of biological investigations.

## 1. Introduction

Glycomics has been of growing interest within the scientific community recently but has historically [1,2,3] lagged behind proteomics and genomics due to the inherent difficulties of isolation and subsequent application of complex glycans. Glycomics investigations commonly rely on sophisticated techniques such as hydrophilic interaction chromatography (HILIC), polymeric anion exchange (WAX), and porous graphitic carbon (PGC) to purify complex glycans [4,5] and mass spectrometry (MS) [6] to detect desired glycan-containing fractions, and it is further challenging to perform chemical modifications of the selected glycans to facilitate biological studies [7]. In contrast, the field of proteomics has brought about innovative [8], selective, mild [9], and occasionally reversible modifications [10] of proteins enabling site-specific tagging [11] and pull-down approaches [12]. Therefore, the field of proteomics has rapidly progressed with assigning structures and functions to an ever-increasing number of targets [13]. Many of these chemical biology approaches exploit thiol chemistry, including intrinsically present cysteine residues, due to the soft nucleophilic nature of thiol groups [14,15,16].

**Figure 1:**
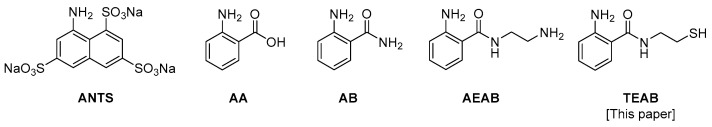
Iterations of glycan auxiliaries.

Glycans containing reducing ends are typically conjugated with an aromatic auxiliary using reductive amination, where the reducing end of the glycan reversibly forms an aldehyde as the ring opens, allowing for reactivity with an amine of interest to form an intermediate imine, which is then reduced with a borane reagent to form the secondary amine-labeled glycan [6]. This then allows for UV-visible (UV-Vis) detection and efficient separations using high-performance liquid chromatography (HPLC). The auxiliary 2-amino-N-(2-aminoethyl)-benzamide (AEAB) has become the most used auxiliary after being popularized by Cummings and Song [17]. AEAB-functionalized glycans after reductive amination possess a primary amine to further conjugate to a handful of functional groups such as N-hydroxysuccinimide (NHS) esters and epoxide-linked solid supports or are converted to azides to enable Staudinger ligation [18,19,20,21,22]. Current glycan auxiliaries, shown in Figure 1, include 8-aminonaphthalene-1,3,6-trisulfonic acid (ANTS), anthranilic acid (AA), and aminobenzamide (AB) and have also been utilized extensively [23] but possess an even more limited bifunctional arm than is present in AEAB. Many of these glycan auxiliaries have been shown to not separate via traditional C18 chromatography and instead must use elaborate techniques such as HILIC/PGC or ion-pairing chromatography to be isolated from crude reaction mixtures, which can vary greatly depending on solvent conditions and the type of column, therefore limiting scalability and reproducibility [5]. Previous studies have also evaluated these synthetically modified glycans as coupling partners to various enzymes and shown general binding to glycosyl transferases [24,25,26] to generate complex glycans as well as posing general binding to most lectins [27]. To further expand upon the success of the bifunctionality of the aromatic reporter, a thiol-containing group was evaluated as a versatile tool to increase the functionality of labeled glycans. 

## 2. Results

**Figure 2:**
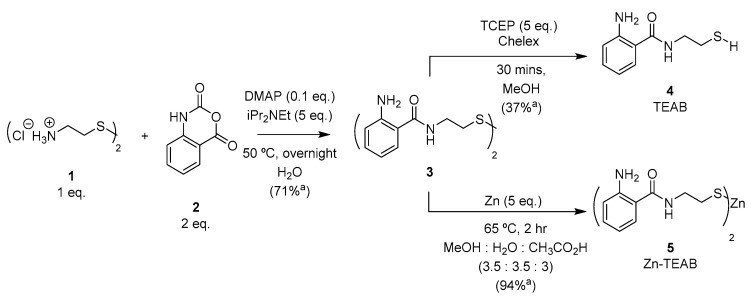
Synthesis of Zn-TEAB **5**. ^a^ Isolated yield.

The synthesis of N-(2-thioethyl)-2-aminobenzamide (TEAB) was previously described by Sherman and Beck [28] utilizing anthranilic acid, cysteamine dihydrochloride (**1**) with hydroxybenzotriazole (HOBt), and benzotriazol-1-yl-oxytripyrrolidinophosphonium hexafluorophosphate (PyBOP), followed by NaBH_4_-mediated reduction and finally purification with silica gel chromatography. In order to establish a more efficient and scalable synthesis of TEAB, an alternative approach using the activated carbonyl isatoic anhydride (**2**) was investigated [29]. Nucleophilic acyl substitution and decarboxylation of **2** proceeded readily in the presence of diisopropylethylamine and a substoichiometric amount of 4-dimethylaminopyridine (DMAP) overnight at 50 °C. The resulting disulfide (**3**) precipitated from the aqueous solvent as the reaction proceeded, providing the product in good yield and purity after sufficient washing of the desired solid. Reduction of disulfide **3** with zinc powder [30] furnished the thiolate-zinc salt **5** (Zn-TEAB), which could be isolated by precipitation in 94% yield (displayed in Figure 2). Alternatively, tris(2-carboxyethyl)phosphine hydrochloride (TCEP, 5 eq.) could be employed to reduce the disulfide **3** to the thiol **4** (TEAB) in 37% yield. TEAB (**4**) was observed as an excellent metal chelator when the thiol form was isolated, resulting in oxidation to disulfide **3** occurring even when dissolved in deionized water. Consequently, thiol salt **5** is a shelf-stable source of thiol (**3**) that does not readily oxidize as a solid or in solution over two months (results displayed in Supplementary Materials) as well as possessing similar fluorescent properties comparable to other popular aminobenzamide derivatives due to the unchanged electronics present on the aromatic ring.

**Figure 3:**
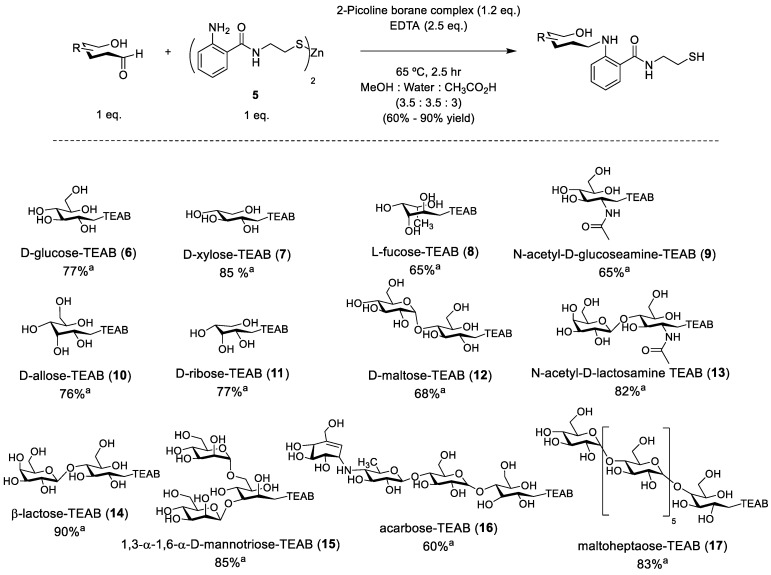
General synthesis of model TEAB-linked glycans. ^a^ Isolated yield.

With gram quantities of Zn-TEAB (**5**) in hand, conjugation to a glycan substrate was evaluated. Typically, reductive amination is conducted using sodium cyanoborohydride as a reducing agent to reduce the intermediate imine [31], but it was deemed more advantageous to utilize the 2-picoline borane complex [32] due to its water-stability and relatively non-toxic properties, therefore allowing for faster glycan conjugation and less hazardous reaction conditions when scaled up. Ethylenediaminetetraacetic acid (EDTA) was also included in the reaction in excess to prevent thiol oxidation and to coordinate the zinc ion. Once completed, the crude mixture was filtered and TCEP (2.5 eq.) was added to ensure isolation of only thiol-conjugated glycans. The mixture was concentrated and purified by preparative reverse-phase chromatography using a gradient of 99:1% to 50:50% (water:acetonitrile) to provide the desired products, shown in Figure 3 (**6**–**17**). This process is unique to TEAB, as other major auxiliaries such as AEAB/AB/AA and ANTS require extensive purification via HILIC/ion-pairing chromatography since these auxiliaries do not enable retention to C18 media. The co-solvent mixture chosen for the reaction was a compromise from a simple water:acetic acid mixture to allow for the proper solvation of the Zn-TEAB auxiliary whilst still solvating the longer glycans of interest, such as maltoheptaose.

A variety of commercially available glycans were selected with varying chain lengths of hexoses and stereochemistry present between all substrates. The yields of the resulting glycoconjugates generally reflected the ease in isolation, with disaccharides such as lactose achieving a 90% yield while the tetrasaccharide acarbose achieved a poorer yield of 60%. Reactivity between the substrates was similar, with no further reductive amination observed past 2.5 h. In addition, uniformity between all separations was observed to be significant, with only a 30-second change in retention time during reverse-phase chromatography from xylose to lactose and a further 30 s to maltoheptaose. In the purification, small single glycans were challenging to separate from starting materials as they eluted a full minute closer to TEAB (**4**), while the longer glycans retained slightly less to the C18 media. Finally, inclusion of TCEP resulted in poorer yields of the larger glycans, causing more co-elution of the glycoconjugates (**16** and **17**) at the dead volume in preparative C18. The overall yields are comparable to other auxiliaries utilized but with the significant difference of the utilization of C18 chromatography instead of the less reproducible HILIC/ion-pairing chromatography. 

**Figure 4:**
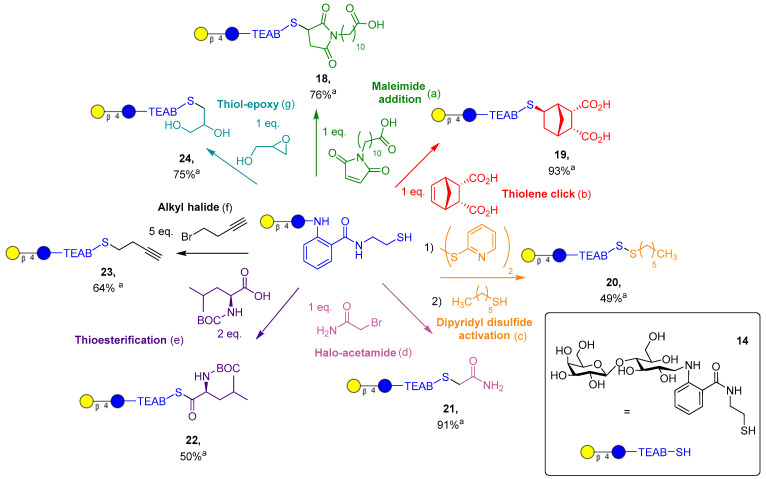
Synthesis of TEAB-modified lactose targets. ^a^ Isolated yield. (a) (NH_4_)_2_CO_3_ (1 eq.), water, 30 min; (b) LAP (0.05% *w*/*v*), water, 30 min irradiation at 365 nm, 10 mW/cm^2^; (c) (1) dipyridyl disulfide (25 eq.), DMF, 10 min; (c) (2) hexane thiol (2 eq.), DMF, 30 min; (d) (NH_4_)_2_CO_3_ (1 eq.), water, 2 h; (e) DCC (2 eq.), DMAP (0.1 eq.), DMF, 2 h; (f) TCEP (0.7 eq.), (NH_4_)_2_CO_3_ (1 eq.), MeOH, 65 °C, 8 h; (g) (NH_4_)_2_CO_3_ (1 eq.), water, 1.5 h.

With the TEAB glycan conjugate probes ready, the chemoselective conjugation of the thiol-derivatized glycans was investigated. Lactose-TEAB (**14**) was selected to identify the optimal conditions for reaction with a diverse range of functional groups (Figure 4). To display the utility of a thiol group over an amino derivative, a wide variety of coupling partners were selected to highlight the selective chemistry of thiols.

First, conjugate addition to the maleimide functional group was performed in the presence of a volatile base, (NH_4_)_2_CO_3_, to promote generation of the thiolate ion to attack the maleimide present. Addition was achieved in 30 min, yielding the desired product **18**, which was then purified from trace salts via prep-C18 in 76% yield. Next, thiol-ene click addition to endo-norbornene-cis-5,6-dicarboxylic acid was performed with the photoinitiator lithium phenyl-2,4,6-trimethylbenzoylphosphinate (LAP, 0.05% *w*/*v*), which proceeded in an exo-selective manner, resulting in product **19**. The desired product was purified with prep-C18 in 93% yield, and stereochemistry was indicated by 2D NOESY and HSQC NMR (supplied in supporting information, SI 25–27) and supported by the literature [33]. Asymmetric disulfide synthesis of **20** is a reversible conjugation to lactose-TEAB, where an unstable disulfide bridge is formed by an excess of dipyridyl disulfide reagent. The desired intermediate could be isolated and purified by prep-C18 but only in a modest yield (30%). Alternatively, the crude product can be used in situ to further react with a simple alkyl thiol, providing the disulfide **20** in 49% yield, once again after reverse-phase chromatography. 

Halo acetamide addition to the lactose-TEAB proceeded in a similar fashion to the maleimide addition but with a longer time period of 2 h, resulting in **21** in 91% yield post-purification. The amino acid thioesterfication with the BOC-protected leucine was an important example of glycosylation of an amino acid, which proved to produce the stable thioester **22** in a moderate yield (50%, unoptimized) using N,N′-dicyclohexylcarbodiimide (DCC, 2 eq.) and DMAP (0.1 eq.). Simple alkylation with an alkyl halide produced the thioether **23** slowly over 8 h. Oxidation of the thiol (**14**) could be observed, and inclusion of TCEP in the reaction conditions provided the desired product in 64% yield after purification. Finally, epoxide ring opening proceeded similarly to maleimide addition and acetamide alkylation, with a slightly shorter time of 1.5 h, giving product **24** in 75% yield post-purification. It is important to note that products **18**, **21**, and **24** can be obtained with minimal impurities or salts without C18 purification if a 1:1 stoichiometry, with respect to substrate and TEAB, is utilized. Overall, out of the seven reactions displayed, amines can only participate in two of the reactions and cannot form a reversible covalent bond, displaying a significant advantage thiol chemistry has over amines. Thiol-ene click chemistry is the most notable example of this, as the reaction is highly controlled via the presence of ultraviolet light and has been shown to be orthogonal to many other functional groups.

**Figure 5:**
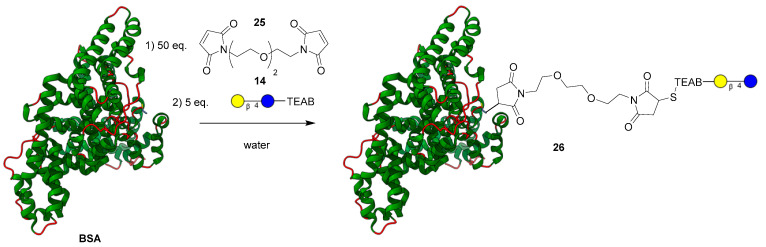
Attachment of bifunctional maleimide linker and lactose-TEAB (**14**) to BSA.

After demonstrating chemoselective conjugation with the thiol to a variety of coupling partners, the model lactose-TEAB probe (**14**) was applied to a more complex system to demonstrate the selectivity of a combined thiol-fluorophore-glycan. Upon reviewing the literature [25], bovine serum albumin (BSA) was chosen as a model protein partner to conjugate a glycan with since it possesses a singular reduced cysteine residue. Similar to the formation of compound **18**, the thiolate ion present on BSA added to the PEG-bismaleimide **25** [34]. After 1 h, the now linked BSA-PEG-maleimide was then submitted to dialysis in a 10–13 MWKO tube for 24 h to remove any small molecule reagents. The modified protein in solution was then reacted with a slight excess of lactose-TEAB (**14**) displayed in Figure 5 for an additional hour and finally purified using dialysis once again. After freeze-drying the sample, the identity of the glycoprotein **26** was confirmed using MALDI mass spectrometry (theoretical: ~67,307 Da; observed mass: 67,309 Da), indicating a significant shift of ~846 Da from the original BSA protein (66,463 Da). Analytical HPLC was used to track the reaction progress as well as the purity of the resultant product. 

To further illustrate the importance of the soft nucleophilic nature of the thiol present on a fluorescent auxiliary, inspiration was taken from a 2-thiopyridine motif installed onto a Sepharose support [16], which was previously utilized as a method to purify proteins through covalent chromatography [12,35]. A commercially available thiol functionalized containing silica gel was determined as a direct way to obtain a 2-thiopyridine-activated support, and the thiol could readily be chlorinated [36] with sulfuryl chloride (10 eq.). The chlorinated thiols were then subsequently treated with 2-thiopyridine (2 eq.) in dry DCM, resulting in the desired thiol selective support (**27**). The activated SiliMet-SH (**27**, ~0.31 mmol SH/g silica) was then applied to crude reductive amination reactions to investigate the effectiveness of a covalent chromatography method.

**Figure 6:**
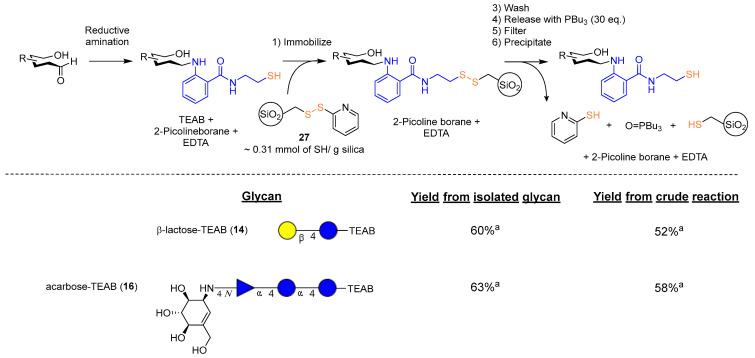
‘Catch and release’ of TEAB-labeled glycans. ^a^ Isolated yield.

Following the process illustrated (Figure 6), it was found that crude reactions could be purified through this method of washing and subsequent release with excess tributyl phosphine. The released glycans could then be precipitated, and any remaining organic compounds were removed via washing with dichloromethane. Most of the product loss was found to occur in the initial immobilization step, where the filtered solvent from the initial ‘catch’ was found to contain the functionalized glycan in the disulfide form (37–40% of original thiol by mass) along with 2-thipyridine. No significant amount of product in the reduced state thiol CH_2_ at 2.70 ppm was observed in NMR analysis (of the initial filtrate), indicating complete capture of the desired thiol. Saturated solutions of ethylenediaminetetraacetic acid disodium salt and maltol, described in the literature [37], were applied to small portions of the activated support **27** as a method to remove redox catalyzing species, but the effect of both chelators on overall yield was found to be negligible. Overall, synthesis of other thiol-containing supports to improve activation or reduce the amount of redox-catalyzing species should be investigated to fully optimize this overall process. 

Finally, to demonstrate that this auxiliary can facilitate glycoarray creation, the use of an appropriate alkene-coated surface was explored [38]. Thiol-coated slides were prepared through dip coating in 3-(Trimethoxysilyl)-1-propanethiol with HCl for 24 h. Iodine starch colorimetric titration was used to confirm the presence of thiols on the glass slide, and then they were further alkylated with a bis allyl PEG linker (**28**, 1 kg/mol) rather than the reported 1,2-polybutene (1.2 kg/mol) to avoid non-specific binding interactions to the surface [39]. The method of generating radicals onto the thiols was also optimized from the dip coating of slides for 24 h with UV irradiation to a drop casting approach with the radical initiator azobisisobutyronitrile (AIBN) in an air oven at 100 °C. Iodine starch titration was once again used to estimate the consumption of thiols to confirm that the thiol content had decreased by half, which indicated the presence of allyl-containing PEG on the drop-casted side of the slide. 

**Figure 7:**
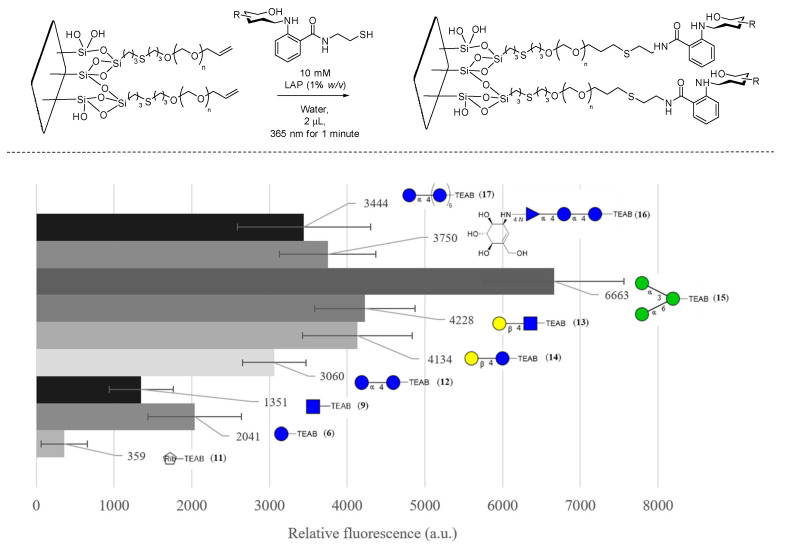
FITC concanavalin A binding assay of glycosylated PEG-coated slides in CFG notation, with error bars denoting 95% confidence intervals (*n* = 3). Blank is defined as a non-glycosylated region of the slide measured at the same time.

The pegylated slides then underwent glycosylation, shown in Figure 7, where fluorescence quantification of the TEAB fluorophore was measured, allowing for the optimization of various parameters, including lithium phenyl-2,4,6-trimethylbenzoylphosphinate (LAP—0.1, 0.5, and 1% *w*/*v*), time of irradiation (1, 5, and 10 min), irradiation power (10, 20, and 40 mW/cm^2^), and concentration of glycans (1, 5, and 10 mM), which were varied until optimized to the values given in Figure 7. Additionally, 1-[4-(2-hydroxyethoxy)-phenyl]-2-hydroxy-2-methyl-1-propane-1-one (I2959), a popular photoinitiator, was also investigated but was found not to produce any fluorescence signal at similar concentrations to LAP, likely due to the poor molar absorptivity at the desired wavelength (365 nm) due to the overlap with the highly absorbing TEAB fluorophore. Utilizing the optimal glycosylation conditions, the quantifiable fluorescence of TEAB was then used to further optimize the PEGylation conditions, where the concentrations of PEG and AIBN (1, 5, 10, and 20% *w*/*v*) and the time (10, 15, 60, and 120 min) were varied. It is important to note that no significant fluorescence was observed when PEG and AIBN concentrations were dropped below 5% *w*/*v*, supporting the fact that thiol-ene addition was occurring on the alkene-PEG rather than disulfide formation to the SH-coated glass.

To fully establish that the surface was glycosylated, FITC-labeled concanavalin A from Canavalia ensiformis (1 mg/mL) was then used as a probe, as described in the literature [40], to investigate the binding of various glycans present on the surface. A slide of nine glycans was generated in triplicate spots, and a control region of PEG allyl-capped thiol was used to ensure sufficient washing of the surface. The variability in spotting, consistency of the surface, and washing of the FITC concanavalin A was optimized to minimize the variability of each well on the slide. Two standard deviations of the measurements at the blank region were determined to have contributed, on average, 51% (instrument error +/− 273 a.u.) of the error of the samples measured. Overall, the binding assay showed a significant signal for the primary binding target of concanavalin A, 1,3-α-1,6-α-D-mannotriose (**15**). The error was low enough to determine that there was a significant replicable binding event to **15** relative to a non-binding monosaccharide such as D-ribose-TEAB (**11**) as well as partial binding to a variety of other glucose-bearing glycans which have also been reported to bind display partial binding to concanavalin A in other research [38].

## 3. Materials and Methods

### 3.1. General Information

All materials and reagents were obtained from either Sigma-Aldrich (St. Louis, MO, USA), Oakwood Scientific (Estill, SC, USA), BioSynth (Gardner, MA, USA), or Fischer Scientific (Waltham, MA, USA). All water used throughout procedures was filtered through a Barnstead NANOpure system (Van Nuys, CA USA) with a resistance of 18.1 MΩ^−cm^. NMR was obtained on a Bruker NEO Avance 500 MHz (Billerica, MA, USA) and a Varian INOVA 400 MHz (Palo Alto, CA, USA). The coupling constant (J) values are given in hertz (Hz) and δ values are given in ppm. The chemical multiplicities have been abbreviated as follows: s = singlet, d = doublet, t = triplet, q = quartet, br = broad signal, quint = quintet, m = multiplet (denotes complex pattern), and their combinations as well. Analytical HPLC was performed on an Agilent 1260 infinity II system (Santa Clara, CA, USA) equipped with a UV-Vis detector and a Kinetex 5 μm EVO C18 100 Å column (Torrance, CA, USA), 150 × 4.6 mm. Prep-HPLC was performed on a Gilson 322 system (Madison, WI, USA) equipped with a UV-Vis-155 detector and a Kinetex 5 μm EVO C18 100 Å column (Torrance, CA, USA), 150 × 21.2 mm. Silica gel column chromatography was performed on SiliaFlash^®^ F60 gel (Silicycle 40–64 μm, Quebec City, Quebec, Canada). Mass spectrometry data were collected through the University of Illinois School of Chemical Sciences’ mass spectrometry service. ESI was collected on a Waters Quattro Ultima mass spectrometer, and MALDI analysis was performed on a Bruker Autoflex Speed LRF MALDI. UV-Vis and fluorescence data were acquired on a SpectraMax i3x reader (San Jose, CA, USA), and UV irradiation was performed with an OmniCure S2000 Spot UV curing system (Saint Louis, MO, USA). Optical rotation was measured on a Jasco DIP-370 digital polarimeter (Easton, MD, USA). 

### 3.2. TEAB Synthesis Methods

#### 3.2.1. Synthesis of N,N′-(dithiodi-2,1-ethanediyl)bis [2-amino-benzamide] (**3**)

Cysteamine dihydrochloride **1** (690 mg, 3.064 mmol, 1 eq.), isatoic anhydride **2** (1000 mg, 6.135 mmol, 2 eq.), DIPEA (2.69 mL, 9.192 mmol, 5 eq.), and DMAP (38 mg, 0.3110 mmol, 0.1 eq.) were dissolved in water (35 mL) in that order. The solution was then heated to 50 °C and stirred overnight, at which point the desired product had precipitated out from the crude mixture. The product was then filtered through P2 filter paper, yielding the insoluble disulfide precipitate, which was then washed with water until analytical HPLC of the product showed the desired purity, yielding product **3** (851 mg, 71% yield).

^1^H NMR (500 MHz, acetone with 0.25% *v*/*v* TMS): δ 7.83 (br, 1H), 7.53 (dd, *J* = 7.9, 1.1 Hz, 1H), 7.25–7.04 (m, 1H), 6.75 (dd, *J* = 8.2, 0.7 Hz, 1H), 6.62–6.39 (m, 1H), 6.19 (br, 1H), 3.70 (dd, *J* = 12.9, 6.5 Hz, 3H), 2.99 (t, *J* = 6.9 Hz, 3H).

Compound previously published in the literature [28].

#### 3.2.2. Synthesis of N-(2-Thioethyl)-2-Aminobenzamide (**4**)

N,N′-(dithiodi-2,1-ethanediyl)bis[2-amino-benzamide] **3** (100 mg, 0.256 mmol, 1 eq.), TCEP (367 mg,1.28 mmol, 5 eq.), and Chelex 100 sodium form (1 g) were suspended in MeOH (10 mL). The suspension was stirred for half an hour; then, the Chelex was filtered off and the filtrate was concentrated to dryness. The crude product was re-dissolved in water (10 mL), and the desired product was extracted with ethyl acetate (50 mL three times). The resulting organic layer was washed with a saturated NaCl solution (10 mL five times), and the resulting organic layer was concentrated, yielding the colorless oil **4** (38 mg, 37% yield).

^1^H NMR (500 MHz, acetone): δ 8.01 (br, 1H), 7.73 (s, 1H), 7.51 (dd, *J* = 7.9, 1.4 Hz, 1H), 7.14 (ddd, *J* = 8.4, 7.1, 1.5 Hz, 1H), 6.74 (dd, *J* = 8.3, 1.0 Hz, 1H), 6.52 (br, 2H), 3.61–3.46 (m, 2H), 2.80–2.69 (m, 2H); ^13^C{^1^H} NMR (126 MHz, Acetone): δ 170.1, 150.5, 132.6, 128.4, 117.4, 116.0, 115.9, 43.5, 24.5.

Compound previously published in the literature [28].

#### 3.2.3. Synthesis of N-(2-Thioethyl)-2-Aminobenzamide Zinc Salt (**5**)

N,N′-(dithiodi-2,1-ethanediyl)bis[2-amino-benzamide] **3** (500 mg, 1.282 mmol, 1 eq.) and zinc powder (420 mg, 6.462 mmol, 5 eq.) were suspended in MeOH, water, and acetic acid (25 mL, 3.5:3.5:3). The suspension was heated at 65 °C for 2.5 h and then left to cool. The excess undissolved metallic zinc was then filtered off from the solution using P2 filter paper. The crude mixture was then concentrated under vacuum to remove most of the methanol and water until a fine oil formed at the bottom of the flask. The product was then precipitated with an excess of water, and the solid was filtered with P2 filter paper. The white precipitate was once again washed with water, finally yielding pure zinc salt **5** (547 mg, 94% yield).

^1^H NMR (500 MHz, DMSO): δ 8.27 (br, 1H), 7.46 (1H, d, J = 7.6 Hz, H**4**), 7.10 (1H, t, J = 7.3 Hz, H**3**), 6.66 (1H, d, = 8.2 Hz, H**1**), 6.45 (br, 1H), 6.35 (1H, s, H**2**), 3.47 (1H, br, H**8**), 2.72 (2H, br, H**9**). ^13^C{^1^H} NMR (126 MHz, DMSO): δ 168.7 (C**7**), 149.6 (C**6**), 131.6 (C**3**), 128.1 (C**4**), 116.3 (C**1**), 114.8 (C**5**), 114.6 (C**2**), 43.6 (C**8**), 26.3 (C**9**). HRMS (ESI) m/z [M + H]^+^: calcd for C_18_H_23_N_4_O_2_S_2_Zn^+^, 455.0554; found 455.0463.

### 3.3. General Synthesis of Glyco-Conjugates (***6***–***17***)

A glycan of interest (1.0 eq.), Zn-TEAB **5** (1.0 eq.), 2-picoline borane (1.2 eq.), and EDTA (2.5 eq.) were dissolved in a MeOH, water, and acetic acid mixture (3.5:3.5:3, [glycan] = 0.1 M). The solution was heated to 65 °C and stirred for 2.5 h. The reaction was then filtered through a cotton plug to remove the undissolved EDTA. All reactions were then concentrated under vacuum, filtered through a 0.45 µM filter, and purified by reverse-phase chromatography on a semi-preparative C18 column. All desired glycans were eluted with a gradient of 99:1% to 50:50% (water: acetonitrile) over a 15-min period. If the resulting product contained trace amounts of fluorophore due to partial co-elution, the starting material could be extracted away with a large excess of ethyl acetate with the product fully dissolved in water. All glyco-conjugates should be stored as a solid with no solvent present in a −20 °C freezer to avoid oxidation if storing for longer than a week.

#### 3.3.1. Example Synthesis of Lactose-TEAB (**14**)

Lactose (100 mg, 0.292 mmol, 1 eq.), 2-picoline borane complex (36 mg, 0.351 mmol, 1.2 eq.), Zn-TEAB **5** (133 mg, 0.292 mmol, 1 eq.), and EDTA (214 mg, 0.730 mmol, 2.5 eq.) were dissolved in a MeOH, water, and acetic acid mixture (3 mL, 3.5:3.5:3). The solution was heated at 65 °C for 2.5 h, and afterwards, the solution was filtered through a cotton plug to remove undissolved EDTA and TCEP (209 mg, 0.730 mmol, 2.5 eq.), which were added to ensure complete reduction of any disulfide. The reaction was then concentrated down to a crude oil (~2 mL), which was filtered through a 0.45 µM filter and purified by reverse-phase chromatography on a semi-preparative C18 column with a gradient of 99:1% to 50:50% (water:acetonitrile) over a 15-min period. The desired fraction was collected and concentrated to yield the off-white foaming solid lactose-TEAB **14** (137 mg, 90% yield).

^1^H NMR (500 MHz, D_2_O with 0.25% *v*/*v* MeOH): δ 7.64 (d, J = 7.7 Hz, 1H), 7.56 (t, J = 7.8 Hz, 1H), 7.12 (d, J = 8.3 Hz, 1H), 7.03 (t, J = 7.5 Hz, 1H), 4.56 (d, J = 7.6 Hz, 1H), 4.29 –4.15 (m, J = 7.9, 3.7 Hz, 1H), 4.06 –3.87 (m, 5H), 3.85 –3.78 (m, 1H), 3.78 –3.51 (m, 9H), 3.33 (dd, J = 12.6, 8.6 Hz, 1H), 2.84 (t, J = 6.2 Hz, 2H). ^13^C{^1^H} NMR (126 MHz, D_2_O with 0.25% *v*/*v* MeOH): δ 171.7,148.0, 133.2, 128.7, 118.0, 117.1, 113.1, 103.2, 79.6, 75.1, 72.7, 71.3, 71.2, 70.8, 70.3, 68.6, 62.2, 60.8, 45.7, 38.5, 37.3. HRMS (ESI) m/z [M+H]^+^: calcd for C_21_H_35_N_2_O_11_S^+^, 523.1962; found 523.1957. ${\left[\alpha \right]}_{D}^{20}\;$+ 0.2 (*c* 0.37, MeOH).

#### 3.3.2. Synthesis of D-glucose-TEAB (**6**)

Following the general synthesis procedure, the following reagents were reacted: D-glucose (100 mg, 0.555 mmol, 1 eq.), Zn-TEAB **5** (252 mg, 0.555 mmol, 1 eq.), 2-picoline borane complex (70 mg, 0.666 mmol, 1.2 eq.), and EDTA (402 mg, 1.375 mmol, 2.5 eq.), yielding compound **6** as a foaming white solid (154 mg, 77% yield).

^1^H NMR (500 MHz, D_2_O with 0.25% *v*/*v* CH_3_CN): δ 7.50 (d, J = 7.7 Hz, 1H), 7.44 (t, J = 11.4, 4.3 Hz, 1H), 6.93 (d, J = 8.3 Hz, 1H), 6.83 (t, J = 7.5 Hz, 1H), 4.02 (ddd, J = 8.3, 5.3, 4.2 Hz, 1H), 3.91–3.77 (m, 3H), 3.76–3.70 (m, 1H), 3.70–3.63 (m, 1H), 3.55 (t, J = 6.5 Hz, 2H), 3.45 (dd, J = 13.2, 4.0 Hz, 1H), 3.23 (dd, J = 13.2, 8.2 Hz, 1H), 2.77 (t, J = 6.5 Hz, 2H).^13^C{^1^H} NMR (126 MHz, D_2_O with 0.25% *v*/*v* CH_3_CN): δ 172.2, 147.8, 133.5, 129.0, 117.7, 113.6, 71.8, 71.5, 71.2(2), 71.1(5), 63.3, 46.1, 42.8, 23.8. HRMS (ESI) m/z [M-H]^−^: calcd for C_15_H_23_N_2_O_6_S^−^, 359.1227; found 359.1275. ${\left[\alpha \right]}_{D}^{20}\;$− 5.9 (*c* 1.23, MeOH).

#### 3.3.3. Synthesis of D-xylose-TEAB (**7**)

Following the general synthesis procedure, the following reagents were reacted: D-xylose (100 mg, 0.667 mmol, 1 eq.), Zn-TEAB **5** (302 mg, 0.667 mmol, 1 eq.), 2-picoline borane complex (83 mg, 0.800 mmol, 1 eq.), and EDTA (487 mg, 1.668 mmol, 2.5 eq.), yielding compound **7** as a foaming white solid (187 mg, 85% yield).

^1^H NMR (500 MHz, D_2_O with 0.25% *v*/*v* MeOH): δ 7.45 (d, J = 7.7 Hz, 1H), 7.41 (dd, J = 18.6, 11.0 Hz, 2H), 6.88 (d, J = 8.3 Hz, 1H), 6.77 (t, J = 7.5 Hz, 1H), 4.01–3.91 (m, J = 8.1, 4.1 Hz, 1H), 3.88–3.80 (m, J = 11.0, 4.5 Hz, 1H), 3.77–3.60 (m, 2H), 3.50 (t, J = 6.5 Hz, 2H), 3.43–3.32 (m, 2H), 3.23 (dd, J = 13.1, 8.1 Hz, 1H), 2.73 (t, J = 6.5 Hz, 2H). ^13^C{^1^H} NMR (126 MHz, D_2_O with 0.25% *v*/*v* MeOH): δ 171.7, 147.8, 133.2, 128.6, 117.0, 112.9, 72.2, 71.9, 69.9, 62.8, 45.8, 42.4, 23.4. HRMS (ESI) m/z [M-H]^−^: calcd for C_14_H_21_N_2_O_5_S^−^, 329.1171; found 329.1172. ${\left[\alpha \right]}_{D}^{20}\;$− 4.8 (*c* 1.99, MeOH).

#### 3.3.4. Synthesis of L-fucose-TEAB (**8**)

Following the general synthesis procedure, the following reagents were reacted: L-fucose (100 mg, 0.610 mmol, 1 eq.), Zn-TEAB **5** (277 mg, 0.610 mmol, 1 eq.), 2-picoline borane complex (76 mg, 0.732 mmol, 1.2 eq.), and EDTA (446 mg, 1.525 mmol, 2.5 eq.), yielding compound **8** as a foaming white solid (136 mg, 65% yield).

^1^H NMR (500 MHz, D_2_O with 0.25% *v*/*v* CH_3_CN): δ 7.52 (d, J = 7.7 Hz, 1H), 7.46 (t, J = 7.8 Hz, 1H), 6.96 (d, J = 8.4 Hz, 1H), 6.84 (t, J = 7.5 Hz, 1H), 4.26–4.06 (m, 2H), 3.67 (d, J = 9.0 Hz, 1H), 3.62–3.49 (m, 4H), 3.46–3.33 (m, J = 12.9, 11.9, 4.8 Hz, 3H), 2.80 (t, J = 6.5 Hz, 2H), 1.25 (d, J = 6.6 Hz, 4H).^13^C{^1^H} NMR (126 MHz, D_2_O with 0.25% *v*/*v* CH_3_CN): δ 172.3, 148.0, 133.5, 129.1, 117.6, 113.5, 73.6, 71.2, 68.7, 66.6, 46.6, 42.8, 23.8, 19.1. HRMS (ESI) m/z [M–H]^−^: calcd for C_15_H_23_N_2_O_5_S^−^, 343.1328; found 343.1330. ${\left[\alpha \right]}_{D}^{20}$ − 3.1 (*c* 0.25, MeOH).

#### 3.3.5. Synthesis of N-acetyl-D-glucoseamine-TEAB (**9**)

Following the general synthesis procedure, the following reagents were reacted: N-acetyl-D-glucoseamine (100 mg, 0.452 mmol, 1 eq.), Zn-TEAB **5** (205 mg, 0.452 mmol, 1 eq.), 2-picoline borane complex (56 mg, 0.542 mmol, 1.2 eq.), and EDTA (330 mg, 1.130 mmol, 2.5 eq.), yielding compound **9** as a foaming white solid (118 mg, 65% yield).

^1^H NMR (500 MHz, D_2_O): δ 7.44 (d, J = 6.9 Hz, 1H), 7.39 (t, J = 13.7, 5.7 Hz, 1H), 6.91 (d, J = 8.4 Hz, 1H), 6.76 (t, J = 7.5 Hz, 1H), 4.20 (dt, J = 9.6, 5.0 Hz, 1H), 4.03 (d, J = 5.7 Hz, 1H), 3.90–3.84 (m, 1H), 3.71 (ddd, J = 17.0, 10.8, 3.0 Hz, 2H), 3.65–3.42 (m, 6H), 3.21 (dd, J = 13.9, 9.4 Hz, 1H), 2.72 (t, J = 6.5 Hz, 2H), 1.90 (s, 3H).^13^C{^1^H} (126 MHz, D_2_O with 0.25% *v*/*v* CH_3_CN): δ 174.7, 172.1, 147.8, 133.4, 129.1, 117.7, 113.7, 71.9, 71.6, 70.1, 69.9, 63.1, 44.2, 42.8, 23.8, 22.5. HRMS (ESI) m/z [M-H]^−^: calcd for C_17_H_26_N_2_O_6_S^−^, 400.1542; found 400.1541. ${\left[\alpha \right]}_{D}^{20}$ + 28.1 (*c* 0.29, MeOH).

#### 3.3.6. Synthesis of D-Allose-TEAB (**10**)

Following the general synthesis procedure, the following reagents were reacted: D-allose (100 mg, 0.667 mmol, 1 eq.), Zn-TEAB **5** (302 mg, 0.667 mmol, 1 eq.), 2-picoline borane complex (83 mg, 0.800 mmol, 1.2 eq.), and EDTA (487 mg, 1.668 mmol, 2.5 eq.), yielding compound **10** as a foaming white solid (183 mg, 76% yield).

^1^H NMR (500 MHz, D_2_O with 0.25% *v*/*v* CH_3_CN): δ 7.58 (d, J = 7.8 Hz, 1H), 7.52 (t, J = 7.8 Hz, 1H), 7.03 (d, J = 8.4 Hz, 1H), 6.91 (t, J = 7.5 Hz, 1H), 4.19–4.12 (m, J = 5.1 Hz, 1H), 4.00 (s, 1H), 3.95–3.84 (m, 3H), 3.76 (dd, J = 11.9, 7.0 Hz, 1H), 3.69–3.58 (m, 4H), 3.32 (dd, J = 13.4, 8.6 Hz, 1H), 2.86 (t, J = 6.5 Hz, 2H).^13^C{^1^H} NMR (126 MHz, D_2_O with 0.25% *v*/*v* CH_3_CN): δ 172.2, 148.0, 133.5, 129.0, 117.6, 113.6, 73.7, 72.9(0), 72.8(5), 70.4, 62.8, 45.5, 42.8, 23.8. HRMS (ESI) m/z [M-H]^−^: calcd for C_15_H_23_N_2_O_6_S^−^, 359.1277; found 359.1274.$\;{\left[\alpha \right]}_{D}^{20}$ − 1.3 (*c* 0.75, MeOH).

#### 3.3.7. Synthesis of D-ribose-TEAB (**11**)

Following the general synthesis procedure, the following reagents were reacted: D-ribose (100 mg, 0.667 mmol, 1 eq.), Zn-TEAB **5** (302 mg, 0.667 mmol, 1 eq.), 2-picoline borane complex (83 mg, 0.800 mmol, 1.2 eq.), and EDTA (487 mg, 1.668 mmol, 2.5 eq.), yielding compound **11** as a foaming white solid (170 mg, 77% yield).

^1^H NMR (500 MHz, D_2_O with 0.25% *v*/*v* MeOH): δ 7.49 (d, J = 7.7 Hz, 1H), 7.47–7.41 (m, 1H), 6.94 (d, J = 8.4 Hz, 1H), 6.82 (t, J = 7.5 Hz, 1H), 4.00 (ddd, J = 8.7, 5.9, 3.0 Hz, 1H), 3.92–3.79 (m, 3H), 3.75 (t, J = 6.2 Hz, 1H), 3.69 (dd, J = 11.0, 4.1 Hz, 2H), 3.54 (dd, J = 12.1, 4.9 Hz, 4H), 3.22 (dd, J = 13.3, 8.7 Hz, 1H), 2.77 (t, J = 6.6 Hz, 2H). ^13^C{^1^H} NMR (126 MHz, D_2_O with 0.25% *v*/*v* MeOH) δ 172.2, 148.0, 133.5, 129.0, 117.6, 113.6, 73.7, 72.6, 70.3, 63.0, 45.6, 42.8, 23.8. HRMS (ESI) m/z [M-H]^−^: calcd for C_14_H_21_N_2_O_5_S^−^, 329.1171; found 329.1176. ${\left[\alpha \right]}_{D}^{20}$ − 6.4 (*c* 0.62, MeOH).

#### 3.3.8. Synthesis of D-maltose-TEAB (**12**)

Following the general synthesis procedure, the following reagents were reacted: D-maltose (100 mg, 0.292 mmol, 1 eq.), Zn-TEAB **5** (133 mg, 0.292 mmol, 1 eq.), 2-picoline borane complex (36 mg, 0.350 mmol, 1.2 eq.), and EDTA (213 mg, 0.730 mmol, 2.5 eq.), yielding compound **12** as a foaming white solid (104 mg, 68% yield).

^1^H NMR (500 MHz, D_2_O with 0.25% *v*/*v* MeOH): δ 7.52 (dd, J = 7.8, 1.2 Hz, 1H), 7.49–7.42 (m, 1H), 6.95 (d, J = 8.4 Hz, 1H), 6.84 (t, J = 7.5 Hz, 1H), 5.12 (d, J = 3.9 Hz, 1H), 4.05 (ddd, J = 7.7, 5.1, 2.8 Hz, 1H), 3.98 (dd, J = 7.3, 3.7 Hz, 1H), 3.96–3.71 (m, 8H), 3.65 (dd, J = 11.8, 7.3 Hz, 1H), 3.61–3.55 (m, 3H), 3.45 (dt, J = 8.5, 7.3 Hz, 2H), 3.35 (d, J = 5.8 Hz, 1H), 2.80 (t, J = 6.5 Hz, 2H). ^13^C{^1^H} NMR (126 MHz, D_2_O with 0.25% *v*/*v* MeOH): δ 171.8, 147.4, 133.1, 128.7, 117.3, 113.2, 100.6, 82.0, 73.0, 72.6(4), 72.5(8), 71.7, 71.4, 69.5, 69.2, 62.3, 60.5, 46.0, 42.4, 23.5. HRMS (ESI) m/z [M-H]^−^: calcd for C_21_H_33_N_2_O_11_S^−^, 521.1805; found 521.1808. ${\left[\alpha \right]}_{D}^{20}$ + 54.2 (*c* 1.41, MeOH).

#### 3.3.9. Synthesis of N-acetyl-D-lactoseamine-TEAB (**13**)

Following the general synthesis procedure, the following reagents were reacted: N-acetyl-D-lactoseamine (40 mg, 0.104 mmol, 1 eq.), Zn-TEAB **5** (48 mg, 0.104 mmol, 1 eq.), 2-picoline borane complex (13 mg, 0.125 mmol, 1.2 eq.), and EDTA (76 mg, 0.260 mmol, 2.5 eq.), yielding compound **13** as a foaming white solid (48 mg, 82% yield).

^1^H NMR (500 MHz, D_2_O with 0.25% *v*/*v* CH_3_CN): δ 7.47 (d, J = 7.8 Hz, 1H), 7.43 (t, J = 7.8 Hz, 1H), 6.94 (d, J = 8.4 Hz, 1H), 6.80 (t, J = 7.5 Hz, 1H), 4.49 (d, J = 7.6 Hz, 1H), 4.47–4.34 (m, 1H), 4.00–3.83 (m, 5H), 3.83–3.75 (m, 1H), 3.75–3.47 (m, 11H), 3.22 (dd, J = 13.1, 9.2 Hz, 1H), 2.76 (t, J = 6.5 Hz, 2H), 1.96 (s, 3H). ^13^C{^1^H} NMR (126 MHz, D_2_O with 0.25% *v*/*v* CH_3_CN): δ 174.8, 172.1, 147.9, 133.4, 129.0, 118.7, 117.6, 113.6, 103.5, 79.4, 75.5, 73.1, 71.6, 70.1, 69.0, 62.3, 61.3, 50.7, 44.3, 42.8, 23.8, 22.5. HRMS (ESI) m/z [M + H]^+^: calcd for C_23_H_38_N_3_O_11_S^+^, 564.2225; found 564.2227. ${\left[\alpha \right]}_{D}^{20}$ − 3.5 (*c* 0.27, MeOH).

#### 3.3.10. Synthesis of 1,3-α-1,6-α-D-mannotriose-TEAB (**15**)

Following the general synthesis procedure, the following reagents were reacted: 1,3-α-1,6-α-D-mannotriose (40 mg, 0.079 mmol, 1 eq.), Zn-TEAB **5** (36 mg, 0.079 mmol, 1 eq.), 2-picoline borane complex (10 mg, 0.0952 mmol, 1.2 eq.), and EDTA (58 mg, 0.198 mmol, 2.5 eq.), yielding compound **15** as a foaming white solid (46 mg, 85% yield).

^1^H NMR (500 MHz, D_2_O with 0.25% *v*/*v* CH_3_CN): δ 7.49 (dd, J = 7.8, 1.2 Hz, 1H), 7.47–7.40 (m, 1H), 6.93 (d, J = 8.4 Hz, 1H), 6.82 (t, J = 7.5 Hz, 1H), 5.06 (d, J = 1.1 Hz, 1H), 4.89 (d, J = 1.3 Hz, 2H), 4.15–4.07 (m, 1H), 4.07–4.02 (m, 2H), 3.99 (dd, J = 3.3, 1.7 Hz, 1H), 3.98–3.82 (m, 6H), 3.82–3.58 (m, 10H), 3.58–3.51 (m, 3H), 3.27 (dd, J = 13.4, 8.0 Hz, 1H), 2.76 (t, J = 6.5 Hz, 2H). ^13^C{^1^H} NMR (126 MHz, D_2_O with 0.25% *v*/*v* CH_3_CN): δ 172.2, 147.8, 133.5, 129.1, 119.0, 117.7, 113.7, 102.9, 100.4, 79.9, 74.0, 73.2, 71.0, 70.8(2), 70.8(0), 70.6, 70.5, 70.4, 69.7, 69.0, 67.2, 66.9, 61.4, 61.1, 46.5, 42.8, 23.8. HRMS (ESI) m/z [M + H]^+^: calcd for C_27_H_45_N_2_O_16_S^+^, 684.2412; found 685.2490. ${\left[\alpha \right]}_{D}^{20}\;$+ 43.8 (*c* 0.44, MeOH).

#### 3.3.11. Synthesis of Acarbose-TEAB (**16**)

Following the general synthesis procedure, the following reagents were reacted: acarbose (100 mg, 0.155 mmol, 1 eq.), Zn-TEAB **5** (71 mg, 0.155 mmol, 1 eq.), 2-picoline borane complex (20 mg, 0.186 mmol, 1.2 eq.), and EDTA (113 mg, 0.388 mmol, 2.5 eq.), yielding compound **16** as a foaming white solid (77 mg, 60% yield).

^1^H NMR (500 MHz, D_2_O with 0.25% *v*/*v* MeOH): δ 7.75 (t, J = 7.7 Hz, 1H), 7.61 (dd, J = 18.5, 11.0 Hz, 1H), 7.47 (dd, J = 13.0, 6.5 Hz, 2H), 5.78 (d, J = 3.2 Hz, 1H), 5.29 (dd, J = 16.9, 3.9 Hz, 1H), 4.96 (d, J = 3.8 Hz, 1H), 4.19–3.42 (m, 29H), 3.15 (t, J = 10.3 Hz, 1H), 2.68 (t, J = 6.6 Hz, 2H), 1.29 (d, J = 6.2 Hz, 3H). ^13^C{^1^H} NMR (126 MHz, D_2_O with 0.25% *v*/*v* MeOH): δ 170.8, 147.3, 133.8, 129.1, 121.2, 120.2, 118.0, 116.7, 115.7, 114.7, 100.8, 100.1, 82.5, 78.1, 73.6, 72.9, 72.7, 72.0, 71.8, 71.3, 70.8, 68.9, 68.6, 66.9, 64.8, 63.3, 62.6(1), 61.5(8), 60.9, 56.9, 49.4, 42.8, 23.7, 17.8. HRMS (ESI) m/z [M–H]^−^: calcd for C_34_H_54_N_3_O_18_S^−^, 824.3122; found 824.3122. ${\left[\alpha \right]}_{D}^{20}\;$+ 100.2 (*c* 0.30, MeOH).

#### 3.3.12. Synthesis of Maltoheptaose-TEAB (**17**)

Following the general synthesis procedure, the following reagents were reacted: maltoheptaose (20 mg, 0.017 mmol, 60% purity, 1 eq.), Zn-TEAB **5** (8 mg, 0.017 mmol, 1 eq.), 2-picoline borane complex (2 mg, 0.021 mmol, 1.2 eq.), and EDTA (12 mg, 0.043 mmol, 2.5 eq.), yielding compound **17** as a foaming white solid (11 mg, 83% yield).

^1^H NMR (500 MHz, D_2_O with 0.25% *v*/*v* CH_3_CN): δ 7.50 (d, J = 7.6 Hz, 1H), 7.44 (t, J = 7.9 Hz, 1H), 6.94 (d, J = 8.4 Hz, 1H), 6.82 (t, J = 7.5 Hz, 1H), 5.39 (s, 5H), 5.11 (d, J = 3.6 Hz, 1H), 4.10–3.75 (m, 28H), 3.75–3.52 (m, 20H), 3.42 (t, J = 8.0 Hz, 2H), 3.34 (d, J = 5.8 Hz, 1H), 2.78 (t, J = 6.3 Hz, 2H). ^13^C{^1^H} NMR (126 MHz, D_2_O with 0.25% *v*/*v* CH_3_CN): δ 172.3, 147.8, 133.5, 129.1, 119.6, 118.8, 117.6, 113.5, 100.7, 100.2, 100.1, 82.5, 77.4(2), 77.3(7), 77.2, 73.8(1), 73.7(8), 73.3, 73.2, 72.9, 72.2, 72.0, 71.9, 71.8, 71.7, 71.4, 69.8, 69.4, 62.6, 60.9(4), 60.8(8), 60.8, 46.2, 42.8, 23.9, 23.7. HRMS (ESI) m/z [M + H]^+^: calcd for C_51_H_85_N_2_O_36_S^+^, 1333.4603; found 1333.4603. ${\left[\alpha \right]}_{D}^{20}\;$+ 70.5 (*c* 0.14, H_2_O).

### 3.4. Derivatives of TEAB Glycans

#### 3.4.1. Synthesis of Lactose-TEAB-Maleimidoundecanoic Acid (**18**)

Lactose-TEAB (50 mg, 0.0958 mmol, 1 eq.), 11-maleimidoundecanoic acid (27 mg, 0.0958 mmol, 1 eq.), and ammonium carbonate (9 mg, 0.0929 mmol, 1 eq.) were dissolved in water (1 mL), and the mixture was then left to stir for half an hour. The reacted product was then concentrated, giving the crude product with trace salt impurities; therefore, it was further purified with prep-C18 with a gradient of 99:1% to 0:100% (water:acetonitrile), resulting in an overall yield of **18** (58 mg, 76% yield).

^1^H NMR (500 MHz, MeOD): δ 7.33 (dd, J = 7.8, 1.4 Hz, 1H), 7.19–7.12 (m, 1H), 6.70–6.64 (m, 1H), 6.50–6.42 (m, 1H), 4.34 (d, J = 7.6 Hz, 1H), 3.96–3.91 (m, 1H), 3.86 (dd, J = 8.8, 3.3 Hz, 1H), 3.81–3.68 (m, 4H), 3.65–3.49 (m, 5H), 3.46–3.38 (m, 3H), 3.38–3.31 (m, 3H), 3.27 (dd, J = 12.9, 4.6 Hz, 1H), 3.21–2.98 (m, 4H), 2.78 (ddd, J = 13.3, 7.2, 6.1 Hz, 1H), 2.38 (ddd, J = 18.6, 3.5, 2.3 Hz, 1H), 2.09–1.98 (m, 2H), 1.43 (dt, J = 14.6, 7.2 Hz, 4H), 1.15 (s, 13H). ^13^C{^1^H} NMR (126 MHz, MeOD): δ 182.1, 179.1, 177.2, 172.2, 150.5, 133.8, 129.3, 117.5, 116.2, 113.0, 105.4, 82.8, 76.9, 74.8, 73.1, 72.9, 72.6, 71.3, 70.2, 63.7, 62.4, 47.1, 39.7(8), 39.7(5), 39.7(2), 38.5, 37.0, 32.3, 30.7, 30.5, 30.2, 28.5, 27.7, 27.4. HRMS (ESI) m/z [M-H]^−^: calcd for C_36_H_56_N_3_O_15_S^−^, 802.3432; found 802.3430. ${\left[\alpha \right]}_{D}^{20}\;$− 3.0 (*c* 0.33, MeOH).

#### 3.4.2. Synthesis of Lactose-TEAB-Noroborene Dicarboxylic Acid (**19**)

Lactose-TEAB (50 mg, 0.0958 mmol, 1 eq.), endo-norbornene-cis-5,6-dicarboxylic acid (17 mg, 0.0958 mmol, 1 eq.), and LAP (0.05 *w*/*v* %) were dissolved in water (10 mL). The solution was then irradiated under 365 nm light, 10 mW/cm^2^, for half an hour. The crude product was submitted onto a prep-C18 column with a gradient of 99:1% to 50:50% (water:acetonitrile with 0.1% TFA), yielding product **19** (63 mg, 93% yield).

^1^H NMR (400 MHz, D_2_O with 0.25% *v*/*v* acetone): δ 7.46 (1H, d, J = 7.5 Hz, H**15**), 7.39 (1H, t, J = 7.9 Hz, H**16**), 6.87 (1H, d, J = 8.4 Hz, H**18**), 6.78 (1H, t, J = 7.6 Hz, H**17**), 4.44 (1H, d, J = 7.7 Hz, H**4**), 4.14–4.04 (1H, m), 3.93–3.75 (5H, m), 3.72–3.63 (1H, m, H**12_b_**), 3.63–3.53 (6H, m), 3.53–3.32 (3 H, m, H**9, 10_a_, 22**), 3.14 (1H, dd, J = 12.8, 8.5 Hz, H**10_b_**), 2.91–2.75 (4H, m, H**21, 25, 26**), 2.39 (2H, d, J = 13.5 Hz, H**24, 27**), 2.08–2.07 (1H, m, H**23_a_**), 1.62 (1H, d, J = 10.3 Hz, H**28_a_**), 1.34 (1H, d, J = 8.8 Hz, H**28_b_**), 1.23–1.08 (1H, m, H**23_b_**). ^13^C{^1^H} NMR (126 MHz, D_2_O with 0.25% *v*/*v* acetone): δ 180.9 9 (C, C**29**), 175.4 (C, C**30**), 171.2 (C, C**19**), 147.3 (C, C**13**), 132.5 (CH, C**16**), 128.2 (CH, C**15**), 118.0 (C, C**14**), 116.7 (CH, C**18**), 112.6 (CH, C**17**), 102.7 (CH, C**4**), 79.0 (CH), 74.6 (CH), 72.2 (CH), 70.8 (CH), 70.7 (CH), 70.2 (CH), 69.7 (CH, C**7**), 68.0 (CH), 61.7 (CH_2_, C**12**), 60.3 (CH_2_, C**6**), 50.2 (CH, C**26**), 48.9 (CH, C**25**), 46.3 (CH, C**27**), 45.3 (CH_2_, **10**), 40.7 (CH, **22**), 40.6 (CH, C**24**), 38.4 (CH_2_, C**20**), 35.9 (CH_2_, C**28**), 34.2 (CH_2_, C**23**), 30.6 (CH_2_, C**21**). HRMS (ESI) m/z [M + H]^+^: calcd for C_30_H_45_N_2_O_15_S^+^, 705.2541; found 705.2527.$\;{\left[\alpha \right]}_{D}^{20}\;$+ 2.6 (*c* 0.28, MeOH).

#### 3.4.3. Synthesis of Lactose-TEAB-Disulfidehexane (**20**)

Dipyridyl disulfide (264 mg, 1.20 mmol, 25 eq.) was dissolved in DMF (1 mL). Lactose-TEAB (25 mg, 0.0479 mmol, 1.0 eq.) was dissolved in a separate portion of DMF (1 mL), and the latter mixture was then added to the stirring thiol-activating reagent dropwise over a period of 5 min. The reaction mixture was stirred for a further 5 min and then diluted by water (10 mL). DCM (5 mL, five times) was then used to extract any remaining dipyridyl disulfide reagent. At this point, the activated thiopyridine intermediate could be isolated with a prep-C18 gradient from 1% to 50% acetonitrile, yielding the solid in poor yield (8 mg, 30% yield). The aqueous layer was then concentrated under air and re-dissolved in DMF (1 mL). Hexane thiol (7 μL, 0.0479 mmol, 1 eq.) was added and left to stir for an hour to generate the disulfide of interest. The crude mixture was concentrated with air and re-dissolved in MeOH (2 mL) and submitted onto a prep-C18 column with a gradient of 99:1% to 0:100 % (water:acetonitrile), yielding **20** (15 mg, 49% yield).

^1^H NMR (500 MHz, MeOD): δ 7.46 (dd, J = 7.9, 1.2 Hz, 1H), 7.32–7.25 (m, 1H), 6.81 (d, J = 8.1 Hz, 1H), 6.63–6.57 (m, 1H), 4.47 (d, J = 7.7 Hz, 1H), 4.10–4.01 (m, 1H), 3.95–3.81 (m, 2H), 3.79–3.69 (m, 1H), 3.62 (t, J = 6.9 Hz, 1H), 3.60–3.52 (m, 1H), 3.49 (dd, J = 9.7, 3.3 Hz, 1H), 3.40 (dd, J = 12.9, 4.9 Hz, 1H), 3.28 (dd, J = 12.9, 7.6 Hz, 1H), 2.91 (t, J = 6.9 Hz, 1H), 2.80–2.67 (m, 1H), 1.68 (dt, J = 14.9, 7.4 Hz, 1H), 1.39 (dd, J = 14.8, 7.4 Hz, 1H), 1.35–1.25 (m, 2H), 0.90 (t, J = 7.0 Hz, 1H). ^13^C{^1^H} NMR (126 MHz, CD_3_CN): δ 170.8, 150.7, 133.7, 128.9, 116.4, 115.6, 112.7, 105.5, 84.6, 76.4, 74.4, 72.8, 72.6, 72.0, 71.4, 70.0, 63.5, 62.5, 46.9, 39.5, 38.5, 32.1, 29.8, 28.8, 23.3, 14.3. HRMS (ESI) m/z [M + H]^+^: calcd for C_27_H_47_N_2_O_11_S_2_^+^, 639.2621; found 639.2618. ${\left[\alpha \right]}_{D}^{20}$ − 8.2 (*c* 0.11, MeOH).

#### 3.4.4. Synthesis of Lactose-TEAB-Acetamide (**21**)

Lactose-TEAB (50 mg, 0.0958 mmol, 1 eq.), bromo acetamide (13 mg, 0.0958 mmol, 1 eq.), and ammonium carbonate (9 mg, 0.0958 mmol, 1 eq.) were dissolved in water (1 mL). The solution was stirred for 2 h and submitted onto a prep-C18 column with a gradient of 99:1% to 50:50% (water:acetonitrile), yielding **21** (50 mg, 91% yield).

^1^H NMR (500 MHz, D_2_O with 0.25% *v*/*v* CH_3_CN): δ 7.46 (d, J = 7.7 Hz, 1H), 7.42 (t, J = 7.8 Hz, 1H), 6.89 (d, J = 8.4 Hz, 1H), 6.79 (t, J = 7.5 Hz, 1H), 4.47 (d, J = 7.7 Hz, 1H), 4.17–4.06 (m, J = 7.4, 4.4 Hz, 1H), 3.99–3.80 (m, 5H), 3.72 (dd, J = 11.8, 5.8 Hz, 1H), 3.67–3.47 (m, 8H), 3.47–3.37 (m, 1H), 3.32 (s, 2H), 3.18 (dd, J = 12.4, 8.5 Hz, 1H), 2.84 (t, J = 6.4 Hz, 2H). ^13^C{^1^H} NMR (126 MHz, D_2_O with 0.25% *v*/*v* CH_3_CN): δ 176.0, 172.1, 148.3, 133.6, 129.0, 118.5, 117.5, 113.5, 103.5, 79.8, 75.4, 73.0, 71.6, 71.5, 71.1, 70.6, 68.9, 62.5, 61.2, 46.0, 38.8, 34.9, 32.0. HRMS (ESI) m/z [M + H]^+^: calcd for C_23_H_38_N_3_O_12_S^+^, 580.2176; found 580.2179. ${\left[\alpha \right]}_{D}^{20}\;$+ 1.6 (*c* 0.42, MeOH).

#### 3.4.5. Synthesis of Lactose-TEAB-BOC-L-Leucine Thioester (**22**)

Lactose-TEAB (50 mg, 0.0958 mmol, 1 eq.), DCC (39 mg, 0.192 mmol, 2 eq.), DMAP (1 mg, 0.00958 mmol, 0.1 eq.), and BOC-L-Leucine (44 mg, 0.192 mmol, 2 eq.) were dissolved in DMF (1 mL). The solution was stirred for 12 h and then concentrated under air to be re-dissolved in MeOH (2 mL) and submitted onto a prep-C18 column with a gradient of 99:1% to 0:100% (water:acetonitrile), yielding **22** (35 mg, 50% yield).

^1^H NMR (500 MHz, DMSO): δ 8.39 (t, J = 5.4 Hz, 1H), 7.88 (dd, J = 13.0, 7.9 Hz, 1H), 7.59 (d, J = 7.8 Hz, 1H), 7.49 (t, J = 7.9 Hz, 1H), 7.24 (t, J = 7.6 Hz, 1H), 6.69 (d, J = 8.4 Hz, 1H), 6.51 (dd, J = 15.4, 7.9 Hz, 1H), 5.57 (d, J = 7.9 Hz, 1H), 5.12 (d, J = 3.5 Hz, 1H), 4.79–4.65 (m, 3H), 4.65–4.51 (m, 3H), 4.51–4.39 (m, 2H), 4.28 (d, J = 7.4 Hz, 2H), 4.10 (d, J = 4.5 Hz, 1H), 4.09–3.99 (m, 1H), 3.83 (s, 2H), 3.79–3.61 (m, 6H), 3.61–3.42 (m, 8H), 3.17 (ddd, J = 17.1, 11.4, 4.9 Hz, 3H), 2.98 (dt, J = 11.4, 8.0 Hz, 2H), 1.65 (ddd, J = 12.9, 10.5, 6.6 Hz, 6H), 1.50 (ddd, J = 28.3, 19.0, 4.4 Hz, 5H), 1.33–1.19 (m, 5H), 1.19–0.97 (m, 3H), 0.84 (dd, J = 20.4, 6.5 Hz, 6H). ^13^C{^1^H} NMR (126 MHz, DMSO): δ 203.0, 169.1, 156.6, 155.5, 149.6, 132.4, 128.3, 114.6, 113.7, 111.2, 104.3, 82.4, 78.6, 75.4, 73.3, 71.3, 71.2, 71.1, 68.9, 68.0, 62.2, 60.2, 59.3, 47.5, 45.5, 33.3, 28.2, 25.3, 24.5, 24.2, 22.9, 21.0. HRMS (ESI) m/z [M + H]^+^: calcd for C_32_H_54_N_3_O_14_S^+^, 736.3326; found 736.3327.$\;{\left[\alpha \right]}_{D}^{20}\;$− 14.6 (*c* 0.16, MeOH).

#### 3.4.6. Synthesis of Lactose-TEAB-Butyne (**23**)

1-Bromo-butyne (18 μL, 0.192 mmol, 5 eq.), lactose-TEAB (20 mg, 0.0383 mmol, 1 eq.), and triethylamine (27 μL, 0.192 mmol, 5 eq.) were dissolved in methanol (1 mL). The sample was heated at 65 °C in a sealed vial and stirred for 6 h, with TCEP (8 mg, 0.0268 mmol, 0.7 eq.) added to the mixture to reduce any disulfide formed. The mixture was heated and stirred for a further 2 h and then concentrated under vacuum, treated with NaOH (1 M, 0.2 mL), and re-concentrated to remove any triethylamine. The crude product was then submitted onto a prep-C18 column with a gradient of 99:1% to 0:100 % (water:acetonitrile), yielding **23** (14 mg, 64% yield).

^1^H NMR (500 MHz, D_2_O): δ 7.49 (dd, J = 7.8, 1.4 Hz, 1H), 7.46–7.41 (m, 1H), 6.92 (d, J = 8.3 Hz, 1H), 6.81 (t, J = 7.5 Hz, 1H), 4.49 (d, J = 7.7 Hz, 1H), 4.17–4.08 (m, 1H), 3.90 (qdd, J = 15.1, 8.5, 3.1 Hz, 6H), 3.73 (dd, J = 11.8, 5.9 Hz, 1H), 3.69–3.51 (m, 8H), 3.45 (dd, J = 12.8, 3.9 Hz, 1H), 3.19 (dd, J = 12.8, 8.3 Hz, 1H), 2.87 (t, J = 6.5 Hz, 2H), 2.78 (t, J = 6.8 Hz, 2H), 2.55 (td, J = 6.8, 2.6 Hz, 2H), 2.41 (t, J = 2.6 Hz, 1H). ^13^C{^1^H} NMR (126 MHz, D_2_O with 0.25% *v*/*v* CH_3_CN): δ 172.1, 148.2, 133.5, 129.0, 118.7, 117.6, 113.5, 103.6, 84.3, 79.8, 75.4, 73.0, 71.6, 71.5, 71.1, 70.9, 70.6, 68.9, 62.5, 61.2, 46.1, 39.2, 31.1, 30.3, 19.3. HRMS (ESI) m/z [M + H]^+^: calcd for C_25_H_39_N_2_O_11_S^+^, 575.2275; found 575.2276.$\;{\left[\alpha \right]}_{D}^{20}$ − 4.4 (*c* 0.24, MeOH).

#### 3.4.7. Synthesis of Lactose-TEAB-Propanediol (**24**)

Lactose-TEAB (50 mg, 0.0958 mmol, 1 eq.), glycidol (7 μL, 0.0958 mmol, 1 eq.), and ammonium carbonate (9 mg, 0.0958 mmol, 1 eq.) were dissolved in water (1 mL). The solution was stirred for 1.5 h, concentrated under vacuum, and submitted onto a prep-C18 column with a gradient of 99:1% to 50:50% (water:acetonitrile), yielding **24** (43 mg, 75% yield).

^1^H NMR (500 MHz, D_2_O): δ 7.49 (d, J = 7.7 Hz, 1H), 7.43 (t, J = 7.7 Hz, 1H), 6.92 (d, J = 8.4 Hz, 1H), 6.81 (t, J = 7.5 Hz, 1H), 4.49 (d, J = 7.7 Hz, 1H), 4.21–4.10 (m, 1H), 4.01–3.80 (m, 6H), 3.80–3.53 (m, 12H), 3.45 (dd, J = 12.8, 3.6 Hz, 2H), 3.19 (dd, J = 12.7, 8.3 Hz, 1H), 2.95–2.75 (m, 3H), 2.66 (dd, J = 13.7, 7.7 Hz, 1H). ^13^C{^1^H} NMR (126 MHz, D_2_O with 0.25% *v*/*v* CH_3_CN): δ 172.0, 148.2, 133.5, 129.0, 118.5, 117.5, 113.5, 103.5, 79.8, 75.4, 73.0, 71.6, 71.5, 71.2, 71.1, 70.6, 68.9, 64.8, 62.5, 61.2, 46.0, 39.3, 34.7, 31.8. HRMS (ESI) m/z [M + H]^+^: calcd for C_24_H_41_N_2_O_13_S^+^, 597.2329; found 597.2324.$\;{\left[\alpha \right]}_{D}^{20}$ − 5.2 (*c* 0.16, MeOH).

#### 3.4.8. Synthesis of N,N′-[1,2-ethanediylbis(oxy-2,1-ethanediyl)]bismaleimide (**25**)

Compound **25** was synthesized according to the protocols described in the literature [34], utilizing the following amounts: triethylene glycol diamine (0.96 mL, 6.4 mmol, 1 eq.), maleic anhydride (1255 mg, 12.8 mmol, 2 eq.), triethylamine (0.90 mL, 6.4 mmol, 1 eq.), sodium acetate trihydrate (524 mg, 6.4 mmol, 1 eq.), and acetic anhydride (6.04 mL, 64.0 mmol, 10 eq.). The procedure was modified, with DMF (20 mL) utilized over the reported acetone solvent for the condensation reaction. The crude product was concentrated and purified with silica gel chromatography over the reported precipitations. The sample was re-dissolved in MeOH:DCM (5:95) and loaded onto silica gel (Ø 5 cm, h_C_ 15 cm, V_Fr_ 12 mL), and the product was eluted with isocratic MeOH:DCM (5:95), yielding compound **25** (R_f_ = 0.3). The compound was previously published in the literature [34].

#### 3.4.9. Synthesis of Lactose-TEAB-PEG-Bismaleimide BSA (**26**)

BSA (10 mg, 0.000150 mmol, 1 eq.) and N,N′-[1,2-ethanediylbis(oxy-2,1-ethanediyl)]bismaleimide **25** (2.3 mg, 0.00750 mmol, 50 eq.) were dissolved in water (1 mL), and the solution was left to stir for 1 h. The solution then underwent dialysis with 10–13 MWKO tubing against water (3.5 L), with the dialysate being changed every 12 h for 24 h. Lactose-TEAB **14** (0.4 mg, 0.000766 mmol, 5.1 eq.) was then added to the solution and left to stir for an additional hour. The solution underwent dialysis again, was placed inside of a 10–13 MWKO tubing, and was left to undergo dialysis against water (3.5 L) for a further 3 days, with the dialysate being changed every 12 h. The product was then lyophilized, yielding the synthetic BSA glycoprotein **26** (10 mg) in excellent yield. The final product was analyzed via MALDI to determine connectivity to the desired lactose-TEAB-maleimide connector.

### 3.5. Application of TEAB Glycans

#### 3.5.1. Preparation of Activated SiliMet-SH (**27**)

SO_2_Cl_2_ (25 g, mmol, 10 eq.) was poured into a round-bottom flask, and SiliMet-SH (12.500 g, 19.25 mmol SH content from supplier, 1 eq.) was slowly added to the fuming mixture over a period of 5 min. Once completed, the silica gel was placed onto a rotary evaporator and heated to 45 °C, and reduced pressure was applied until the complete removal of any excess SO_2_Cl_2_. Separately, 2-thiopyridine (4.280 g, 38.5 mmol, 2 eq.) was dissolved in dry dichloromethane (150 mL) and slowly added to the dried chlorinated silica. The sample was stirred for 10 min and then filtered through a glass sinter, and the resin was washed with dichloromethane, methanol, and acetone. The silica was then further washed with sodium bicarbonate (20 mL) to quench any unreacted chlorine present, re-acidified with 1M HCl (40 mL), and further washed with water (200 mL) until the run-off was between pH 6 and 7. Finally, the activated silica was dried with acetone (200 mL) and dried further under vacuum overnight, yielding the purified activated SiliMet-SH (**27**, 14.678 g).

#### 3.5.2. Optimized Catch and Release Process

To the solution of recently reductively aminated glycans, activated resin (~0.31 mmol of SH/g of resin) was added and stirred for 2 h. The solution was diluted with water and then centrifuged for 3000 rpm at 5 min, which was repeated with methanol and then acetone. Following this, the resin was recovered and suspended in PBu_3_ (10 eq.) with ethanol:water (1:1), where it was stirred for 30 min and the solution was separated from the resin via a syringe filter (1 µM); the process was repeated an additional two times. The resin was further washed with additional water and methanol; then, the filtrates combined to be concentrated under vacuum. Next, the yellow crude oil was precipitated with dichloromethane and centrifuged at 3000 rpm for 15 min, and the supernatant was filtered through a syringe filter (1 µM) which was then discarded; additional dichloromethane was added to the pellet, and the process was repeated a further two times. Finally, the precipitate was dissolved in a minimal amount of water and syringe-filtered (1 µM) into a scintillation vial, to which the solution was concentrated, yielding the desired product.

#### 3.5.3. Example Catch and Release of Acarbose-TEAB

Following the optimized procedure, a crude reaction of the reductively aminated acarbose-TEAB (**16**, theoretical yield: 64 mg, 0.0776 mmol, 1 eq.) was added to the activated SiliMet-SH (250 mg). The suspension was left to stir for 2 h and was then placed into a centrifuge tube, diluted up to 15 mL with water, and centrifuged at 3000 rpm for 5 min. The supernatant was decanted, and the process was repeated with the same volume of methanol and acetone. The resin was recovered back into a scintillation vial, then, PBu_3_ (157 mg, 0.775 mmol, 10 eq.) was added and diluted with water:ethanol (1:1, 5 mL). The suspension was stirred vigorously for 30 min, and the solution was back-filtered with a PTFE syringe filter (1 µM); this process was repeated 3 times, and the resin was washed with water (10 mL) and methanol (10 mL), followed by pooling the filtrates together and concentrating until dry. The resulting yellow oil was then diluted with dichloromethane (50 mL) and once again centrifuged at 3000 rpm for 15 min. The supernatant was then discarded, additional dichloromethane (30 mL) was added to the pellet, and the process was repeated a further two times. The pellet was dissolved in water (5 mL x 3) and filtered through a PTFE syringe filter (1 µM). The filtered solution was dispensed into a scintillation vial, which was then concentrated under rotary evaporation to yield the desired acarbose-TEAB (**16**, 37 mg, 0.0450 mmol) in 58% yield.

#### 3.5.4. Preparation of Bis Allylated PEG 1000 (**28**)

Following the literature [41], PEG 1000 MW (10.000 g, 10 mmol, 1 eq.) was added to a 3-necked round-bottom flask and flushed with nitrogen, to which THF (100 mL) was added and stirred until dissolved. To the stirring solution, NaH (60% in oil, 1.3 g, 55 mmol, 5 eq.) was slowly added; then, allylbromide (8.64 mL, 100 mmol, 10 eq.) was added dropwise. The reaction was heated at 40 °C, with additional portions of NaH (5 eq.) being added every 30 min until a total of 15 eq. was reached. After 12 h, the solution was left to cool and then concentrated to obtain the crude product, which was then re-dissolved in DCM (30 mL) and filtered. Again, the sample was concentrated to dryness, and the crude solid was then washed with hexanes (200 mL) three times to remove mineral oil leftover from the NaH, yielding the diallyl PEG 1000 MW (8.734 g, 7.74 mmol, ~95% purity) in 77% yield with trace mineral oil by NMR.

^1^H NMR (500 MHz, CDCl_3_): δ 5.94–5.82 (m, *J* = 10.4, 7.1, 4.1, 1.5 Hz, 2H), 5.25 (d, *J* = 17.2, 1.5 Hz, 2H), 5.15 (d, *J* = 10.4 Hz, 2H), 4.08–3.90 (m, 5H), 3.80–3.42 (m, 76H). ^13^C {^1^H} NMR (126 MHz, CDCl_3_): δ 134.87, 117.18, 72.32, 70.70, 70.61, 69.50.

#### 3.5.5. Preparation of Allyl PEG Microscope Slides

Microscope slides were acquired from Fischer Scientific, and the surface was prepared according to the literature [38]: fresh piranha solution was applied to the glass surface for 1 h, and the surface was then washed and dried overnight under vacuum. The slides were then taken and left in a solution of MPTMS (5% *v*/*v*) in THF with HCl (0.4% *v*/*v*, 10 mL/slide), which was left for 24 h under nitrogen. The dip-coated slides were then removed from the solution and sequentially washed with DCM, toluene, hexanes, acetone, and dichloromethane again. Next, the slides were dried under vacuum and a portion was taken, and the thiol content was estimated utilizing the iodine and starch colorimetric indicator, which approximated around ~3.6 µmol/mm^2^. A saturated solution of divinyl PEG 1000 MW (10% *w*/*v*) and AIBN (10% *w*/*v*) in toluene (9.23 mL) was then prepared, and a portion of the solution (1 mL) was deposited onto the thiol glass slide in a culture dish. The slide was ‘baked’ in an air oven at 100 °C for 1 h and then removed and sequentially washed with an excess of toluene, dichloromethane, methanol, water, and acetone. The now pegylated slides were then utilized for glycosylation, and the free thiol content was estimated to have decreased to ~1.6 µmol/mm^2^ with the iodine starch colorimetric assay.

#### 3.5.6. Glycosylation of PEG-Coated Microscope Slides

To the surface of the pegylated slides, a solution of the glycan of interest (10 mM) and LAP (1% *w*/*v*) in water was dispensed (2 µL). The solution was exposed to UV light (365 nm, 10 mW/cm^2^) for a period of 1 min and was then washed away with excess water, methanol, and acetone, leaving fluorescent spots when viewed with a UV flashlight (365nm). The slide was then dried under vacuum, resulting in the prepared glycosylated surface.

## 4. Conclusions

TEAB is a promising glycan auxiliary, where the use of reductive amination with a glycan has allowed for the preparation of thiol-functionalized glycans in 60–90% yield utilizing standard C18 chromatography, unlike amino derivatives. In addition, TEAB has been shown to undergo rapid conjugation chemoselectively with a variety of coupling partners in 49–93% yield, whilst amino/amide derivatives cannot undergo many of these reactions. These versatile reaction pathways demonstrate the benefits of TEAB as a glycan auxiliary in glycomics investigations. Preliminarily, TEAB-conjugated glycans can be used in glycoprotein synthesis by utilizing reduced cysteine residues. Initial testing of a 2-thiopyridine-activated support demonstrated the utility of a thiol conjugate, where mg quantities of the labeled glycans could be recovered from crude mixtures without the need for chromatographic separations, but this must be validated on reductively aminated biological glycan sources and with confirmation of C18 retention. Additionally, preliminary photolithography of the glycan conjugates demonstrated that glycan arrays can be generated and measured to determine binding to primary enzymatic substrates. Future studies should assess the effect of glycosylation on proteins with respect to folding, substrate binding, and protein–protein interactions. Finally, with respect to photolithography, many applications could be permitted to allow for studies of glycans to biological targets. However, more research surrounding the enzymatic compatibility (glycosyltransferases) to TEAB glycans, assessments of how applicable the pull-down approach is on biological samples, and more in-depth surface characterization (morphology, roughness, and spacing from the surface) of the alkene-pegylated slides are still required. Overall, this novel glycan auxiliary proved to be a diverse, bifunctional, and cost-efficient target to accommodate various studies surrounding the glycome, expanding on previous amino-based derivatives.

## Data Availability

Data can be found in the published manuscript and its Supplementary Materials.

## Supplementary Materials

The following are available online at https://www.mdpi.com/article/10.3390/molecules28041956/s1, (1) Copies of the ^1^H and ^13^C NMR for all compounds (S3–S33,S37); (2) HPLC traces for compounds **4**, **5**, and **17** are provided (S8-10); and (3) UV-Vis data for the optimization of glycosylated PEG microscope slides and compounds **14** & **27** (S19–20, S35, S38–S41).

## Sample Availability

Samples of the compounds are not available from the authors but assistance in synthesis of compounds may be provided upon request.
