# Supplementary material for: Expanding Glycomic Investigations through Thiol-Derivatized Glycans
Source: Molecules. 2023 Feb 18;28(4):1956. doi: 10.3390/molecules28041956 (PMC9964202; doi:10.3390/molecules28041956)
Supplement: Supplementary file 1 [file molecules-28-01956-s001.zip › molecules-2199523-supplementary.pdf]

Supplementary information

Contents

**^{1}H NMR data of compound 3**	**S3**
**^{1}H, ^{13}C{^{1}H} NMR data of compound 4**	**S4**
**^{1}H, ^{13}C{^{1}H}, HSQC NMR data of compound 5**	**S5-6**
**Proposed reductive amination mechanism of glycans with an amine**	**S7**
**Analytical HPLC of oxidation of compounds 4, 5 and 17**	**S8-10**
**^{1}H, ^{13}C{^{1}H} NMR data of compound 6**	**S11**
**^{1}H, ^{13}C{^{1}H} NMR data of compound 7**	**S12**
**^{1}H, ^{13}C{^{1}H} NMR data of compound 8**	**S13**
**^{1}H, ^{13}C{^{1}H} NMR data of compound 9**	**S14**
**^{1}H, ^{13}C{^{1}H} NMR data of compound 10**	**S15**
**^{1}H, ^{13}C{^{1}H} NMR data of compound 11**	**S16**
**^{1}H, ^{13}C{^{1}H} NMR data of compound 12**	**S17**
**^{1}H, ^{13}C{^{1}H} NMR data of compound 13**	**S18**
**^{1}H, ^{13}C{^{1}H} NMR and UV-Vis absorption and fluorescence of compound 14**	**S19-20**
**^{1}H, ^{13}C{^{1}H} NMR data of compound 15**	**S21**
**^{1}H, ^{13}C{^{1}H} NMR data of compound 16**	**S22**
**^{1}H, ^{13}C{^{1}H} NMR data of compound 17**	**S23**
**^{1}H, ^{13}C{^{1}H} NMR data of compound 18**	**S24**
**^{1}H, ^{13}C{^{1}H}, Assignment, NOESY, HSQC NMR data of compound 19**	**S25-27**
**^{1}H, ^{13}C{^{1}H} NMR data of intermediate compound 20**	**S28**
**^{1}H, ^{13}C{^{1}H} NMR data of compound 20**	**S29**
**^{1}H, ^{13}C{^{1}H} NMR data of compound 21**	**S30**
**^{1}H, ^{13}C{^{1}H} NMR data of compound 22**	**S31**

**^{1}H, $^{13}C\{^{1}H\}$ NMR data of compound 23**	**S32**
**^{1}H, $^{13}C\{^{1}H\}$ NMR data of compound 24**	**S33**
**MALDI of compound 26**	**S34**
**UV-Vis quantification of activated Sili-MetSH 27**	**S35**
**Column format of compound (27) for thiol capture**	**S36**
**^{1}H, $^{13}C\{^{1}H\}$ NMR data of compound (28)**	**S37**
**UV-Vis optimization of allylated PEG microscope slides**	**S38**
**UV-Vis optimization of glycosylated PEG microscope slides**	**S39-40**
**Fluorescence data of Concanavalin A assay**	**S41**

^{1}H NMR data of N,N′-(dithiodi-2,1-ethanediyl)bis[2-amino-benzamide] (3)

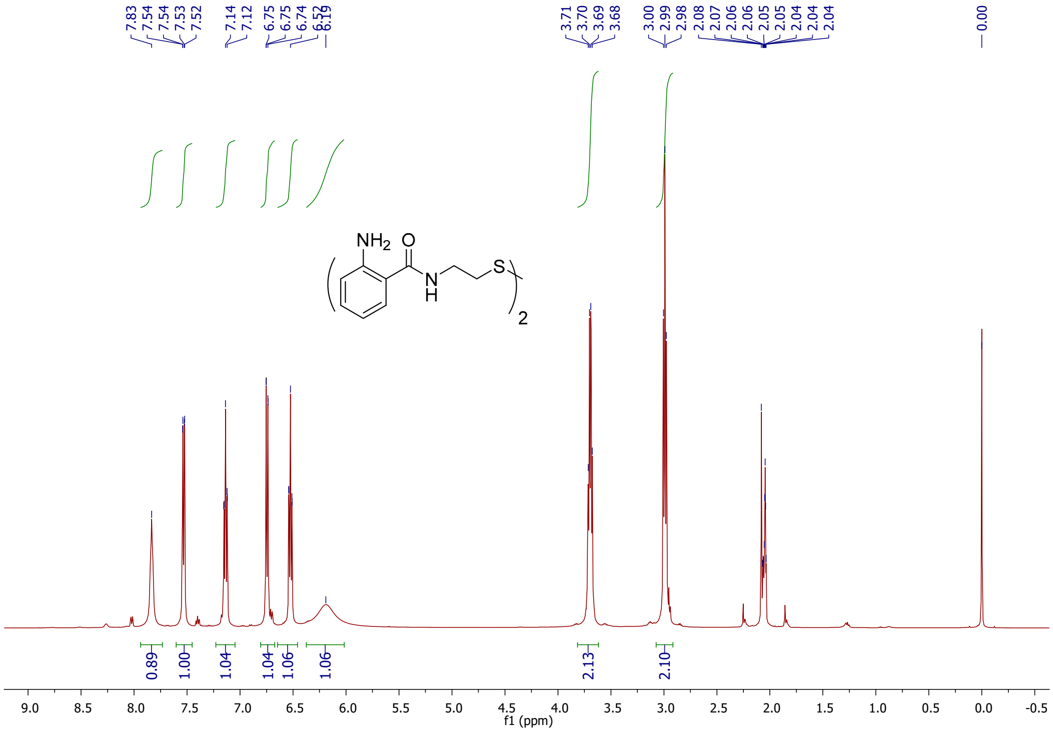

^{1}H and $^{13}C\{^{1}H\}$ NMR data of N-(2-ThioEthyl)-2-AminoBenzamide (4)

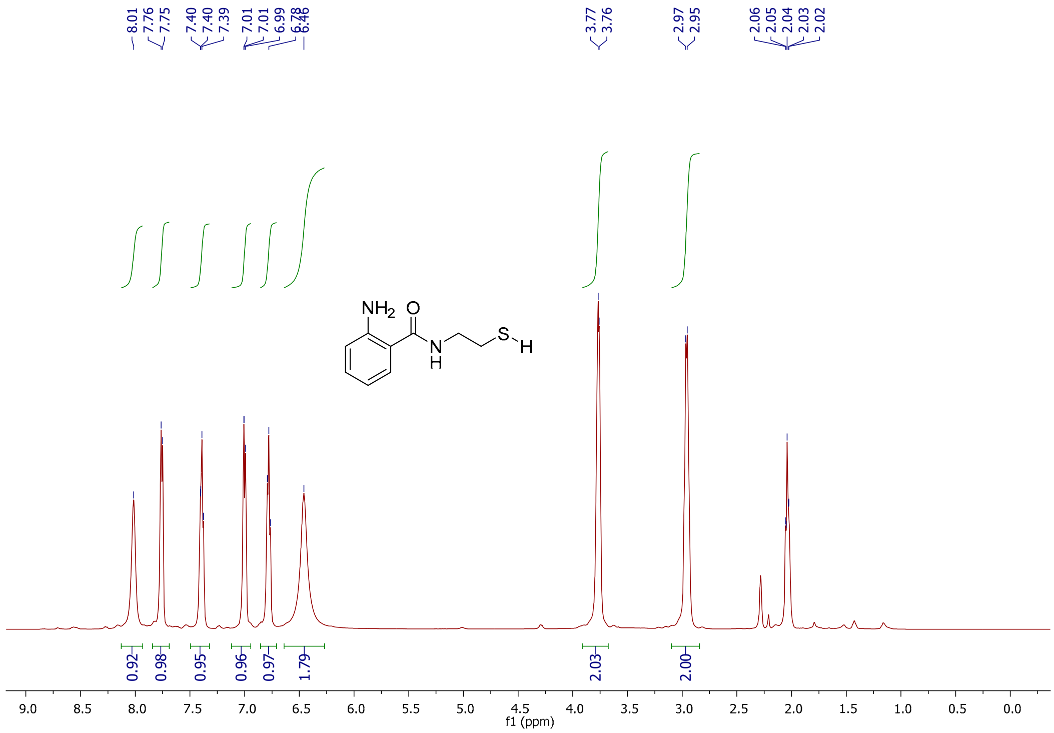

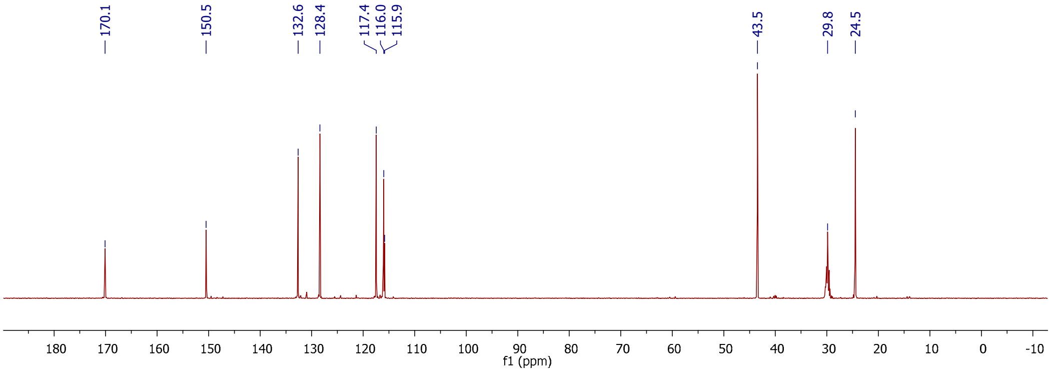

^{1}H, $^{13}C\{^{1}H\}$ and HSQC NMR data of N-(2-ThioEthyl)-2-AminoBenzamide zinc salt (5)

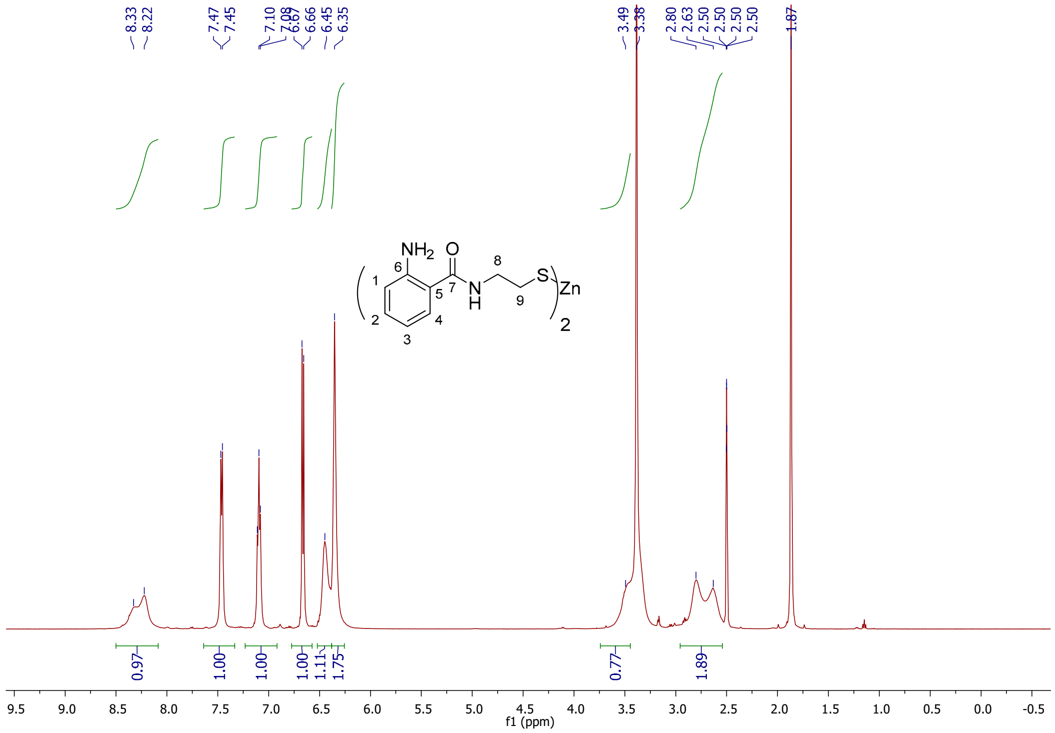

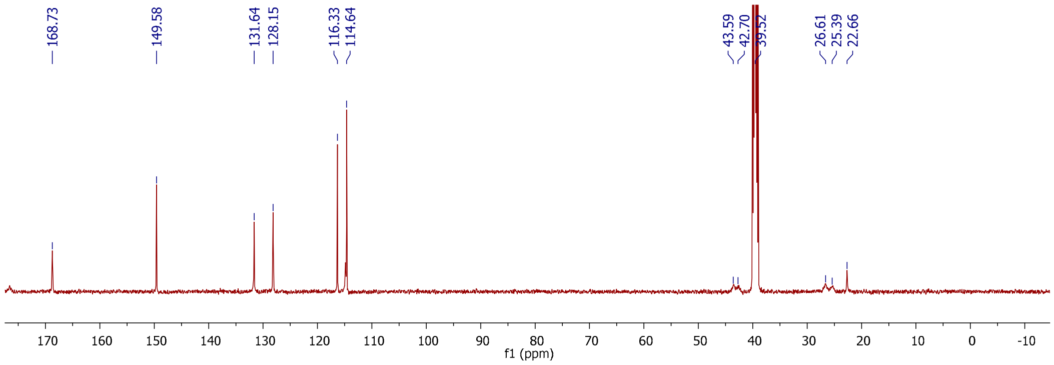

Phase-sensitive ge-2D HSQC using PEP and matched sweep adiabatic pulses for inversion and refocusing with gradients in back-inept of compound 5 – (HSQCEDETGPSISP2.3)

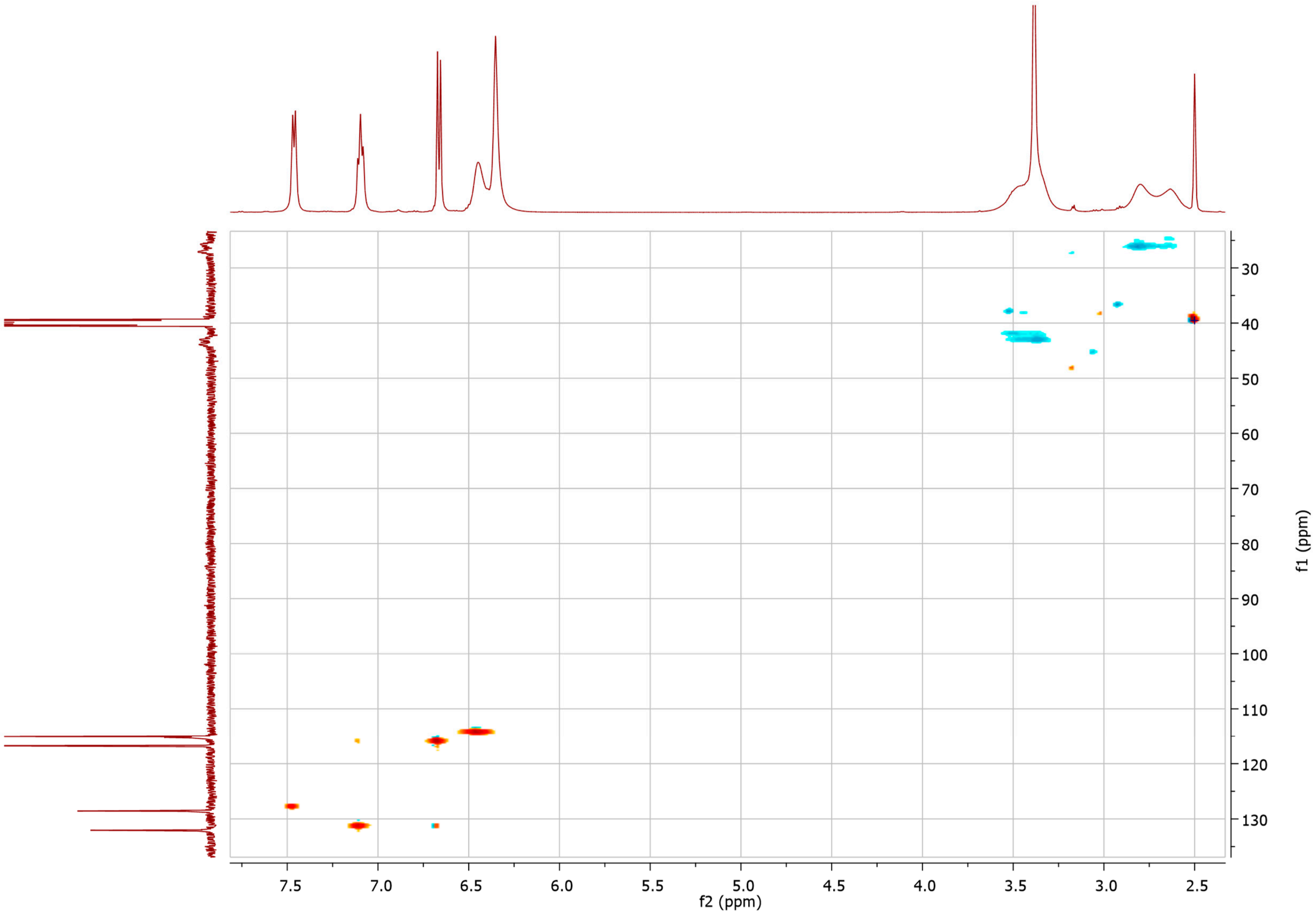

Proposed reductive amination mechanism of glycans with an amine

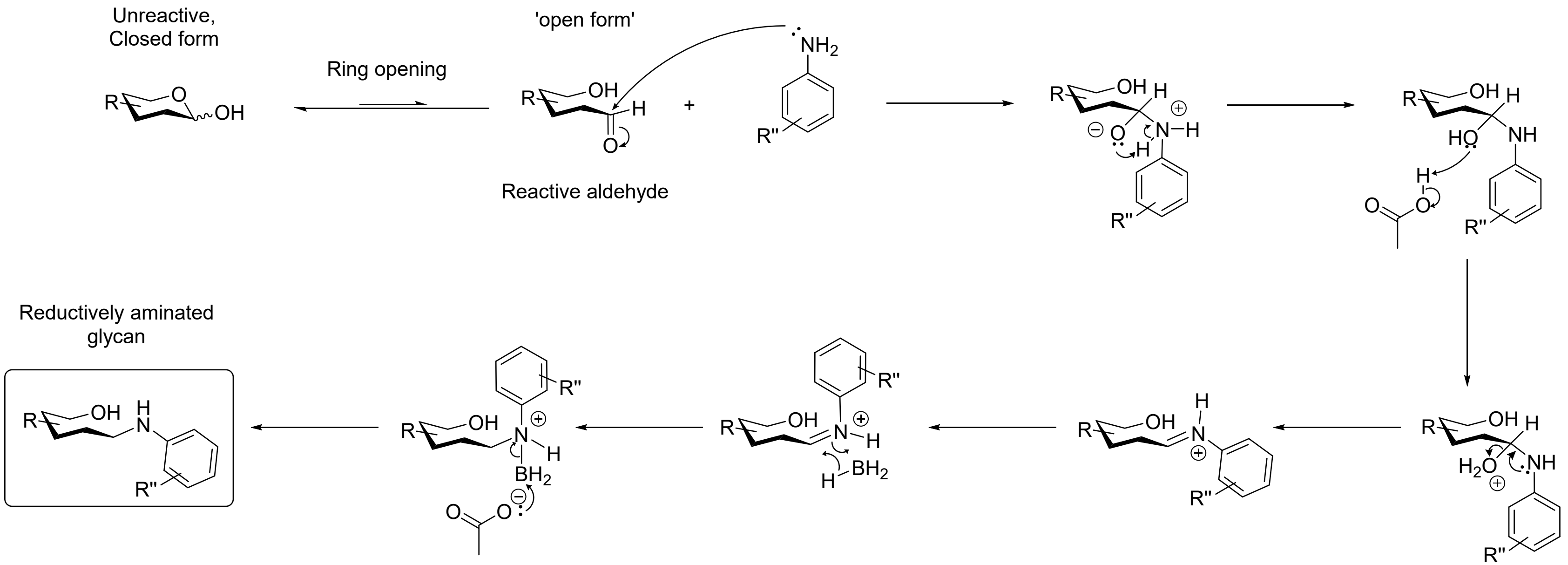

Analytical C18 of oxidation analysis of TEAB 4 (1 mg / mL water)

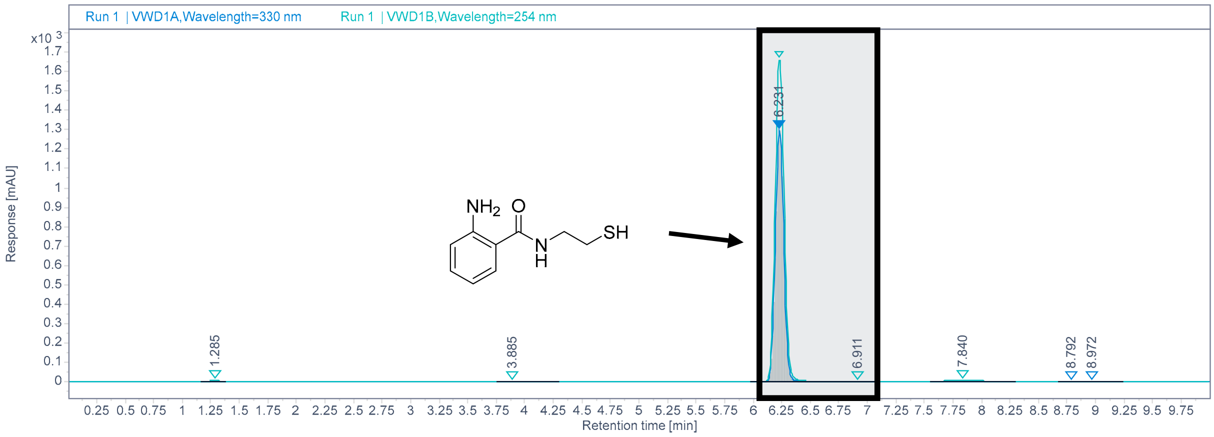

Analytical HPLC of TEAB T=0, gradient 1:99% to 0:100% (water : acetonitrile) over 10 minutes

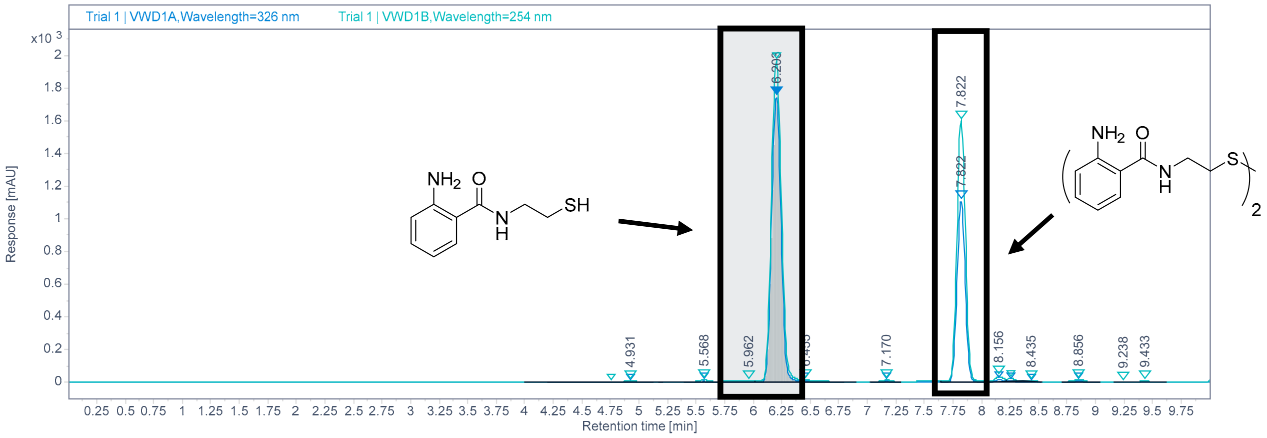

Analytical HPLC of TEAB T = 1 month, gradient 1:99% to 0:100% (water : acetonitrile) over 10 minutes

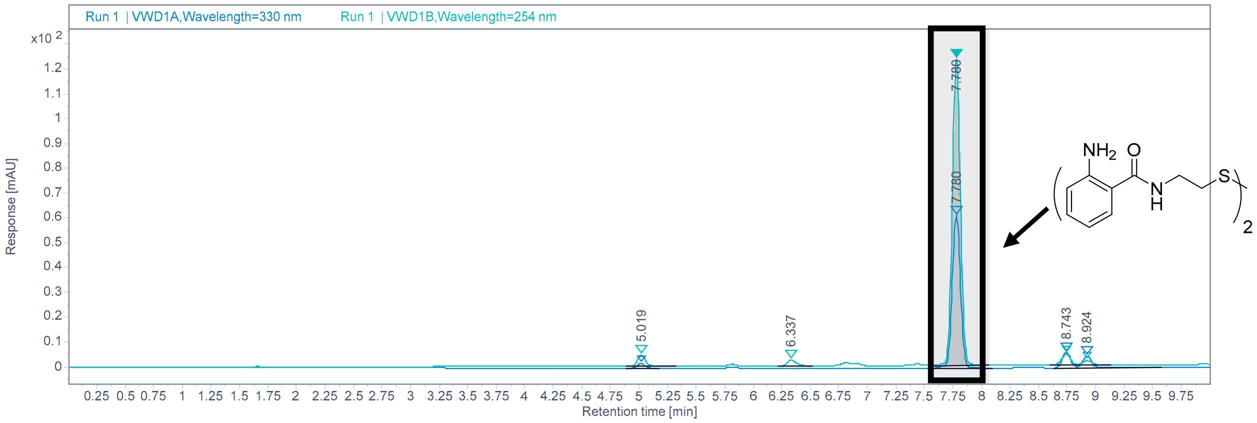

Analytical HPLC of TEAB T = 2 months, gradient 1:99% to 0:100% (water : acetonitrile) over 10 minutes

Analytical C18 of oxidation analysis of Zn-TEAB 5 (1 mg / mL water)

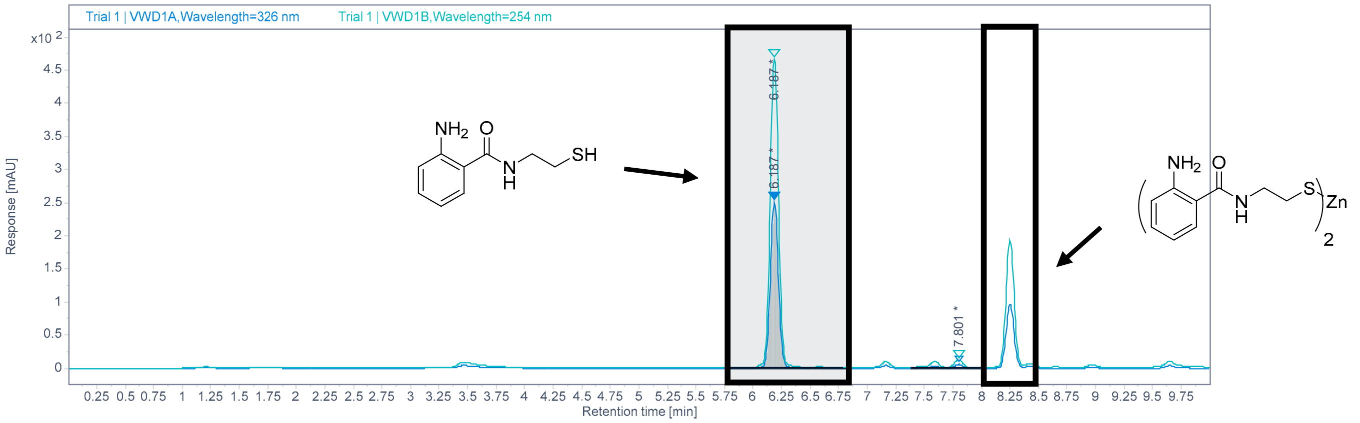

Analytical HPLC of Zn-TEAB T=0, gradient 1:99% to 0:100% (water : acetonitrile) over 10 minutes

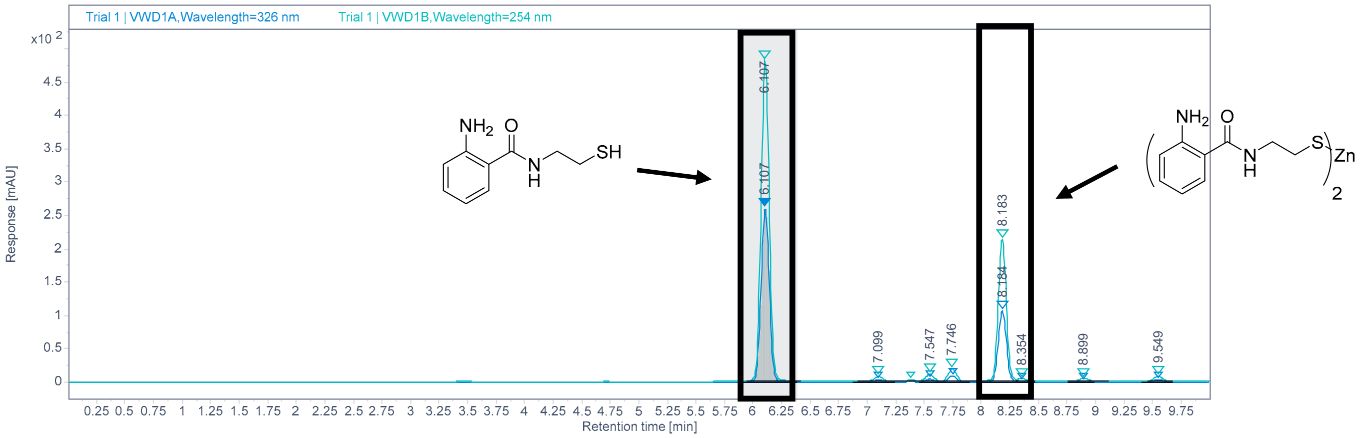

Analytical HPLC of Zn-TEAB T = 1 month, gradient 1:99% to 0:100% (water : acetonitrile) over 10 minutes

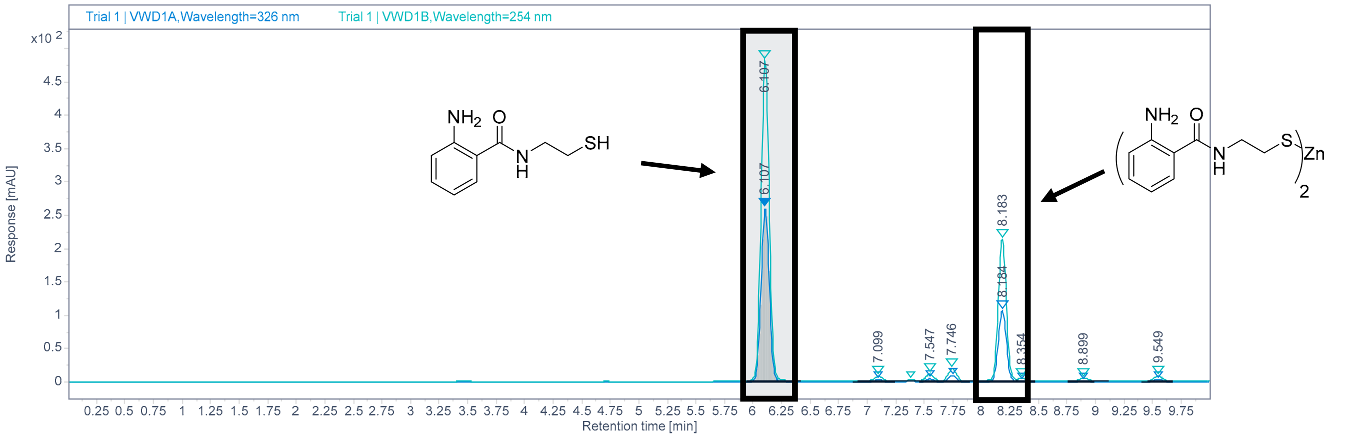

Analytical HPLC of Zn-TEAB T = 2 months, gradient 1:99% to 0:100% (water : acetonitrile) over 10 minutes

Analytical C18 of standard reductive amination of a glycan – maltoheptaose -TEAB 17

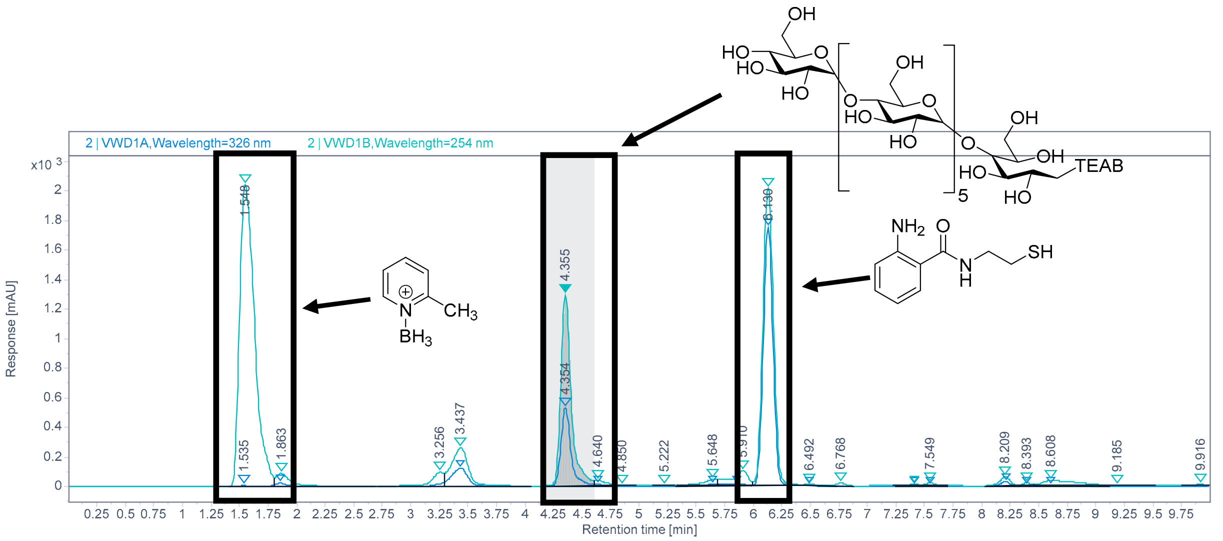

Analytical HPLC of crude reaction, gradient 1:99% to 0:100% (water : acetonitrile) over 10 minutes

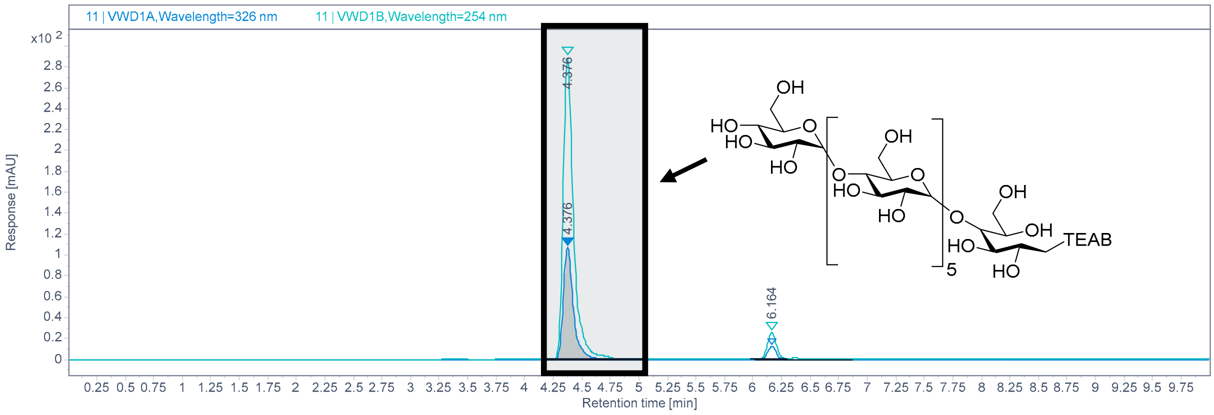

Analytical HPLC of purified product, gradient 1:99% to 0:100% (water : acetonitrile) over 10 minutes

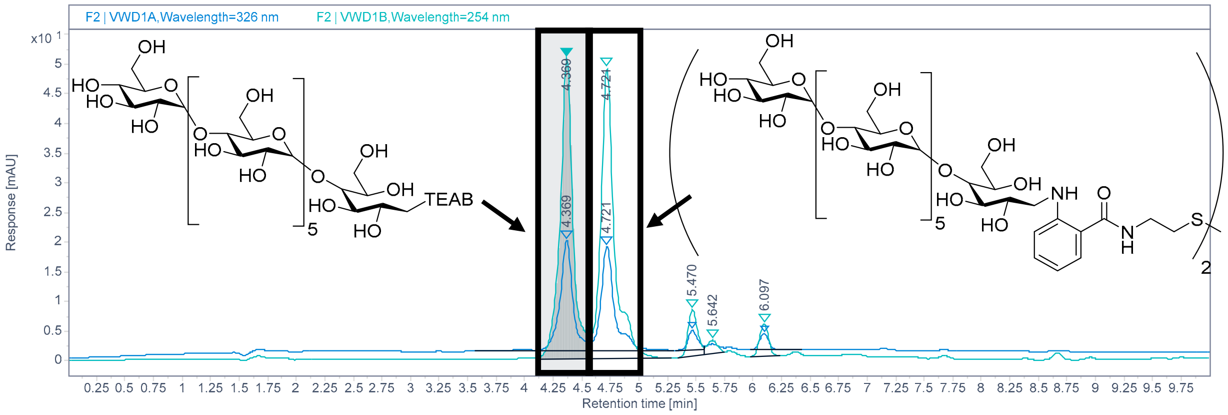

Analytical HPLC of partially oxidised product, gradient 1:99% to 0:100% (water : acetonitrile) over 10 minutes

^{1}H and $^{13}C\{^{1}H\}$ NMR data of D-glucose-TEAB (6)

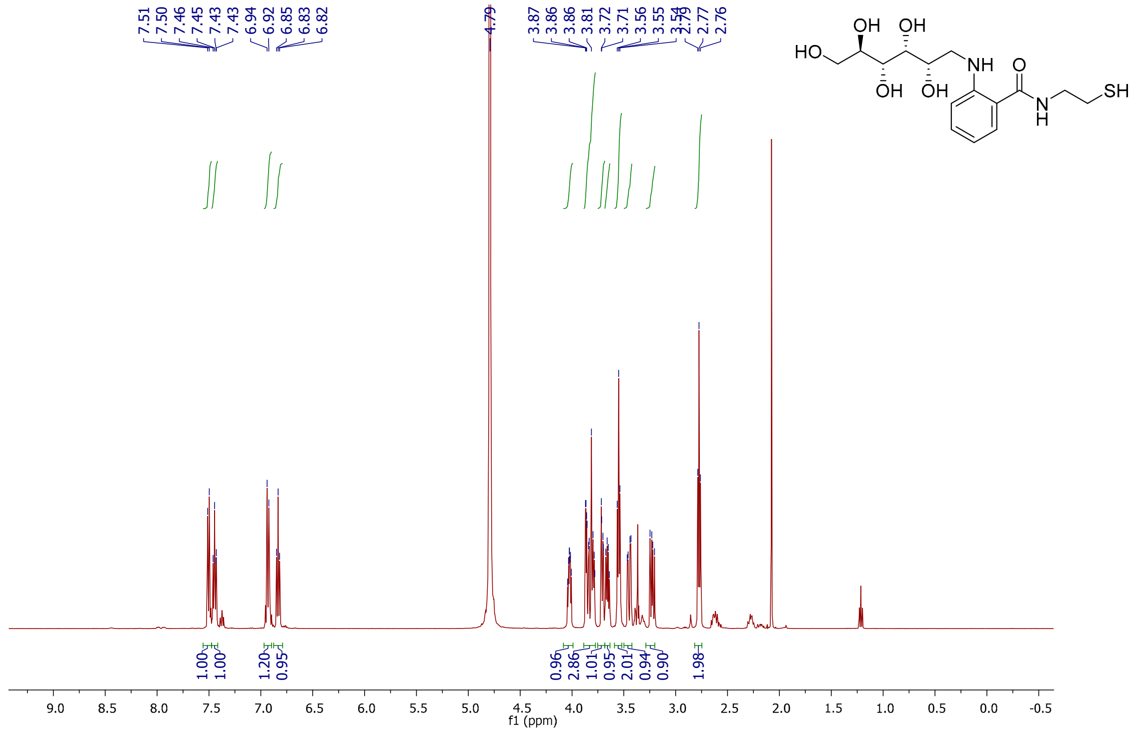

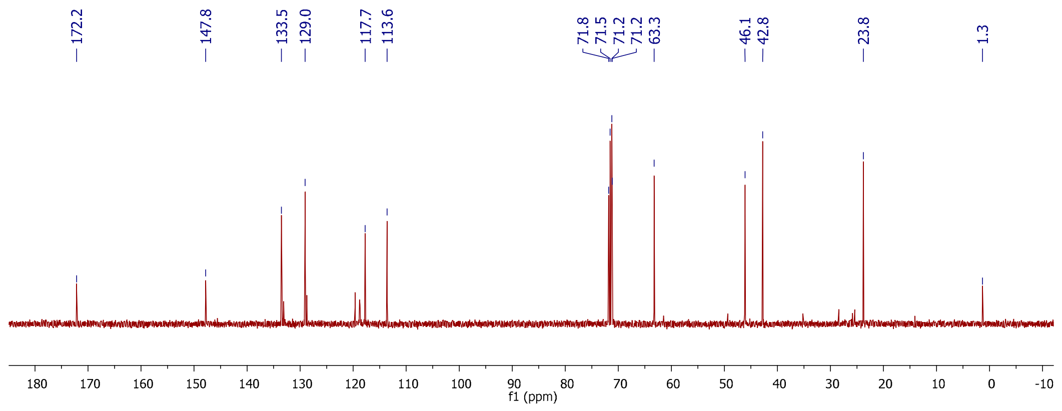

^{1}H and ^{13}C{^{1}H} NMR data of D-xylose-TEAB (7)

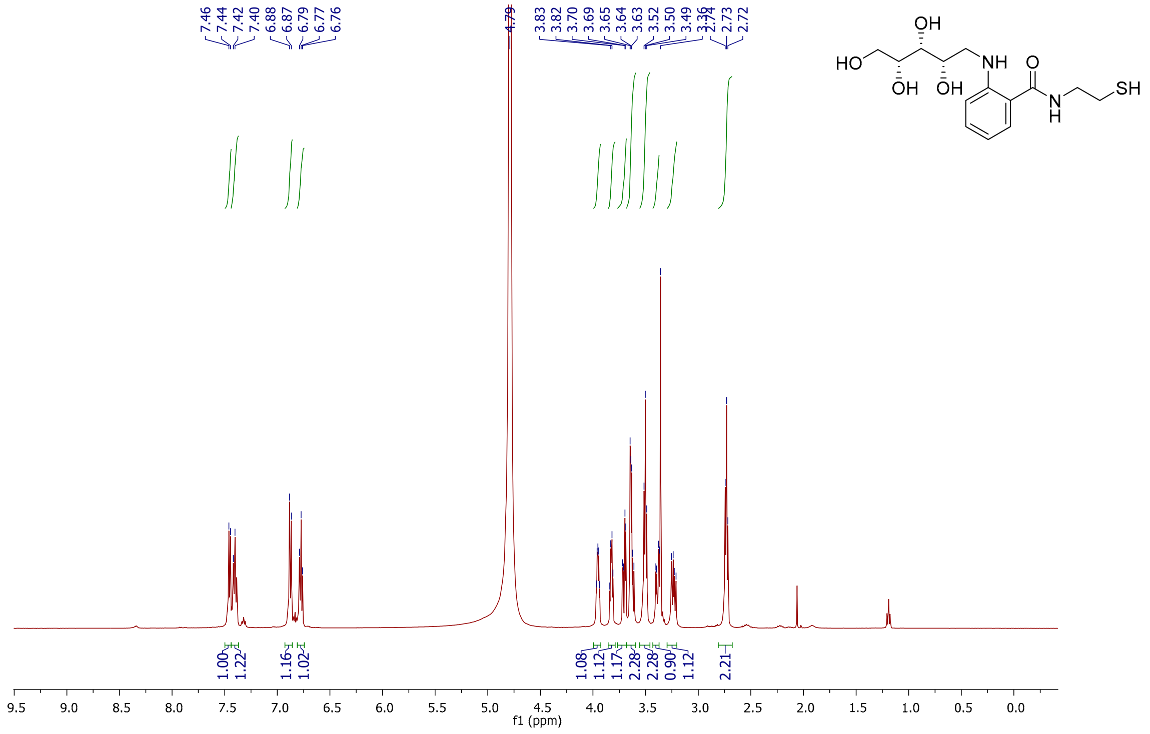

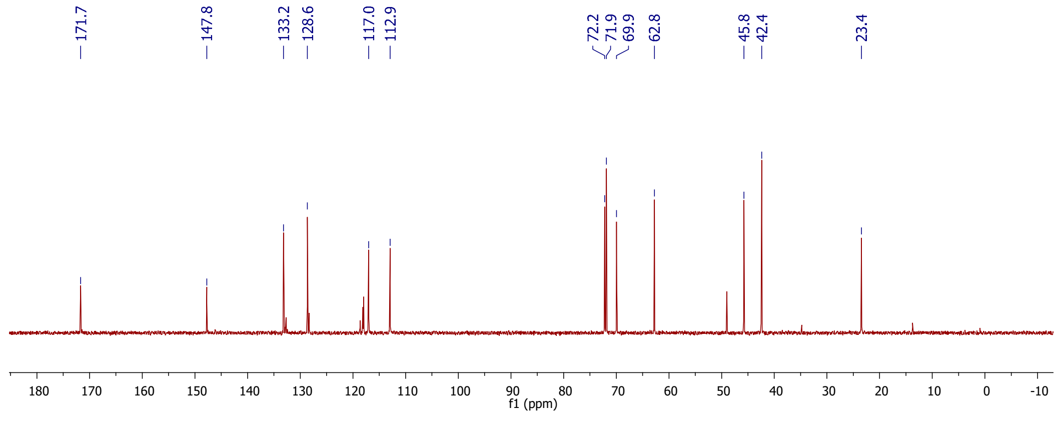

^{1}H and ^{13}C{^{1}H} NMR data of L-fucose-TEAB (8)

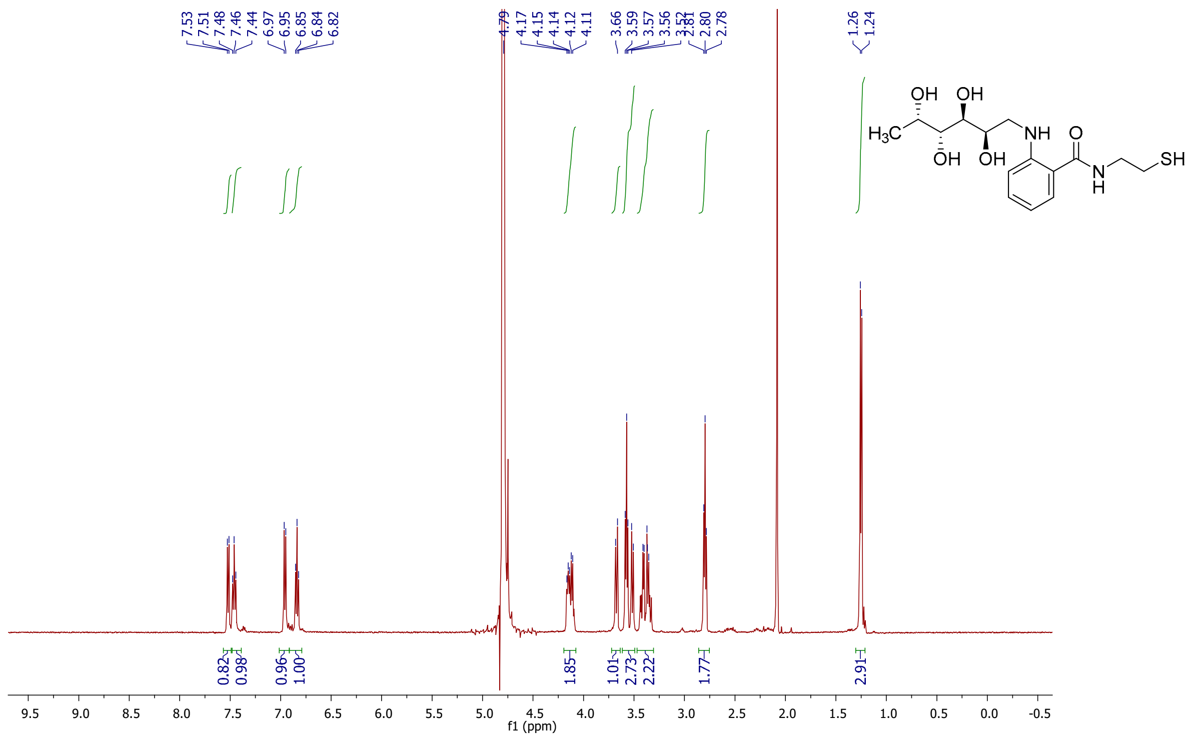

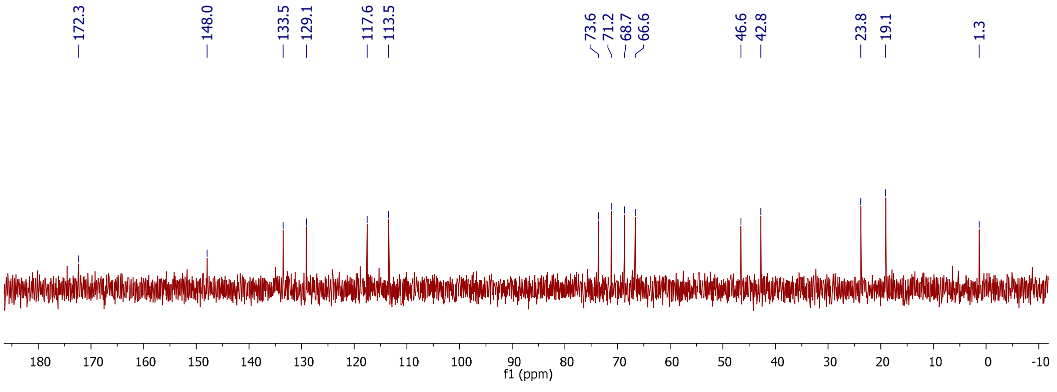

^{1}H and ^{13}C{^{1}H} NMR data of N-acetyl-D-glucoseamine-TEAB (9)

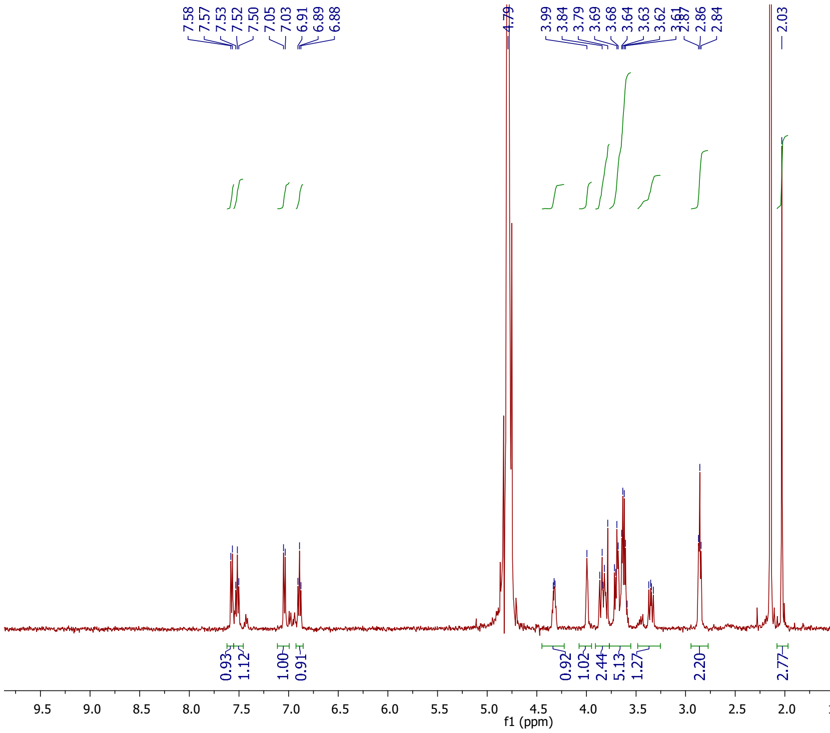

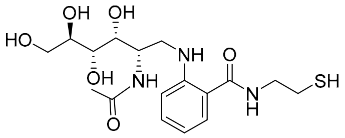

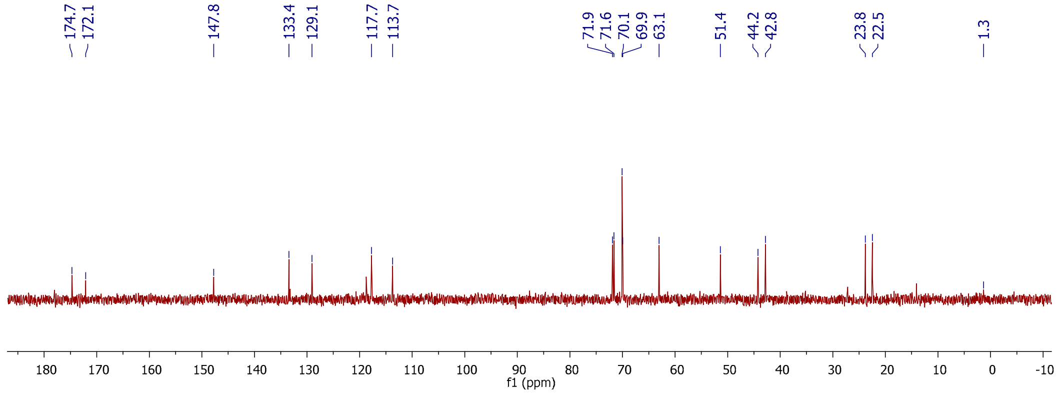

^{1}H and ^{13}C{^{1}H} NMR data of D-allose-TEAB (10)

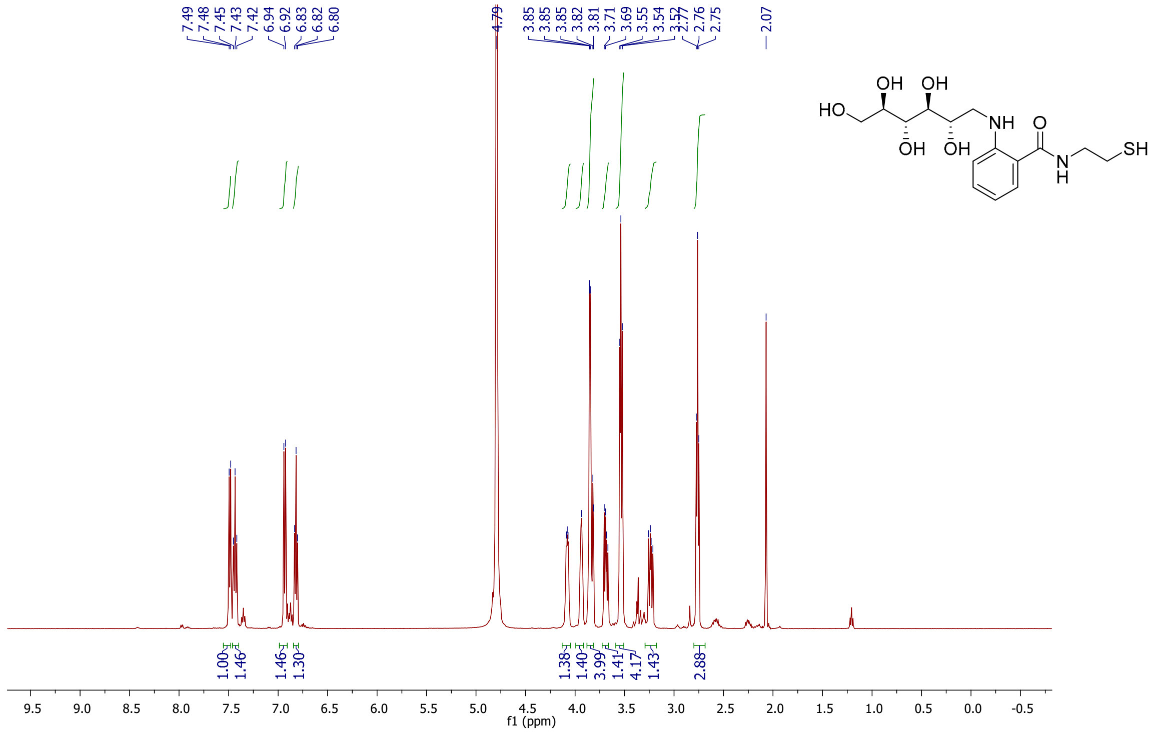

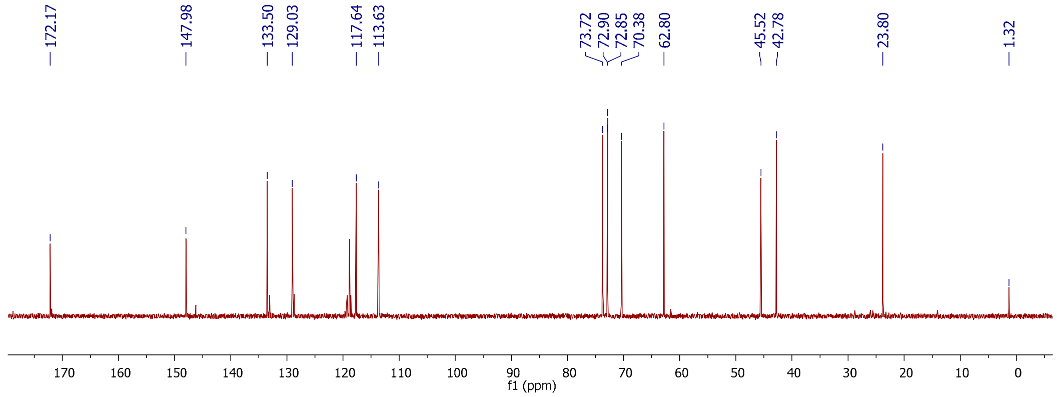

^{1}H and ^{13}C{^{1}H} NMR data of D-ribose-TEAB (11)

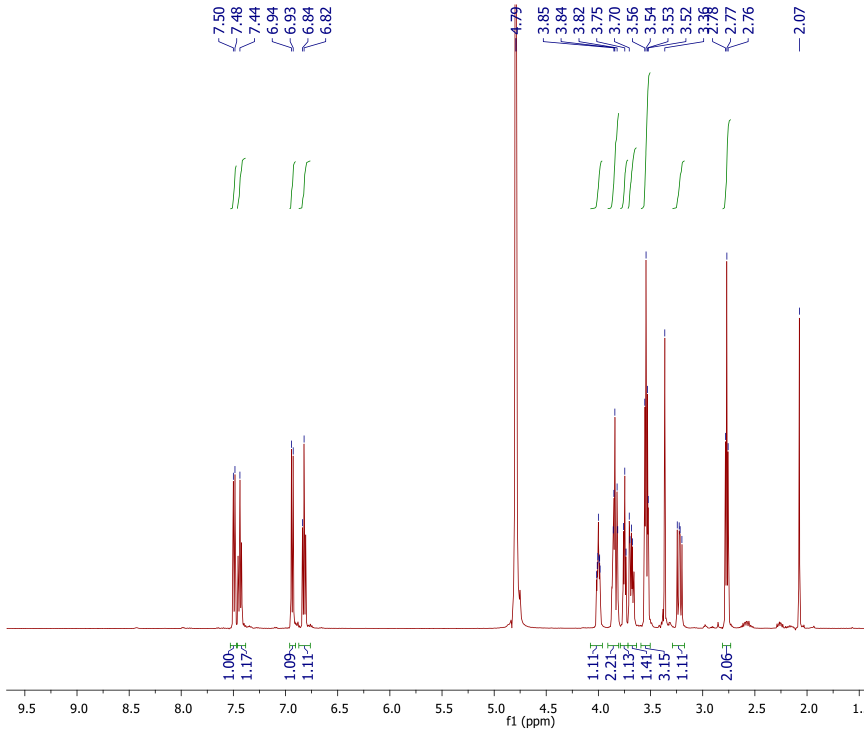

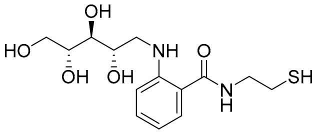

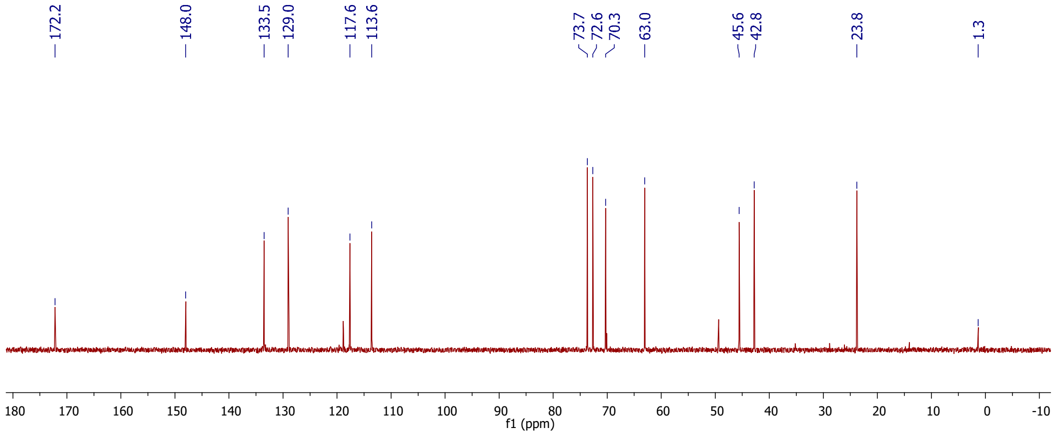

^{1}H and ^{13}C{^{1}H} NMR data of D-maltose-TEAB (12)

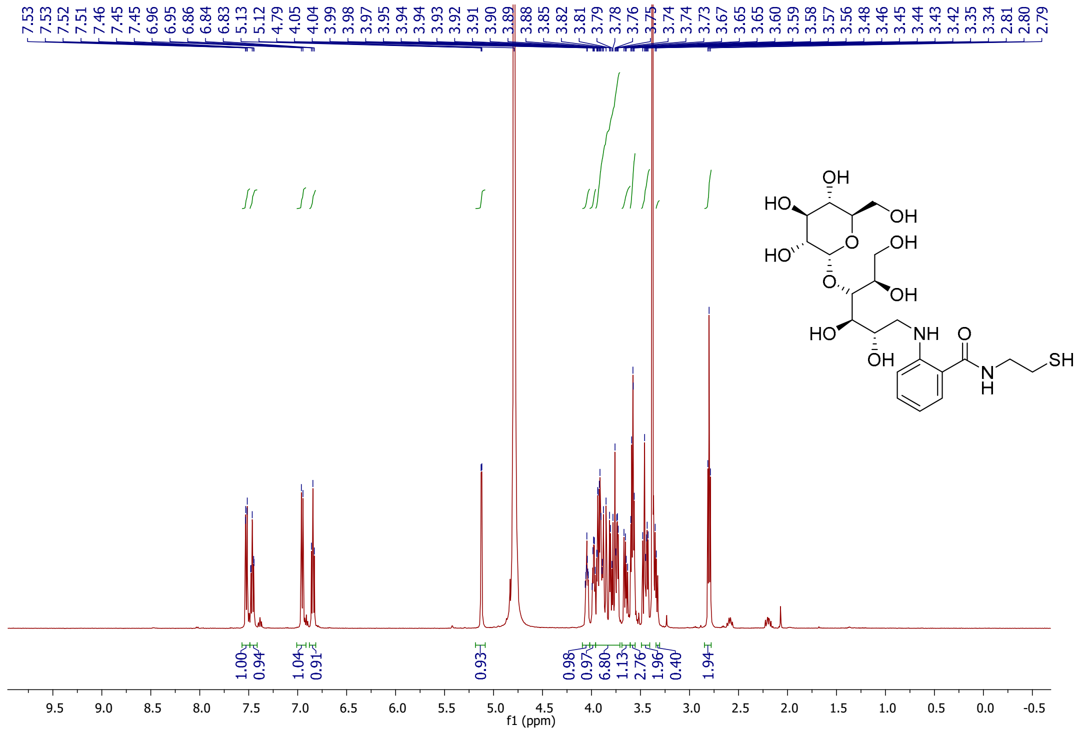

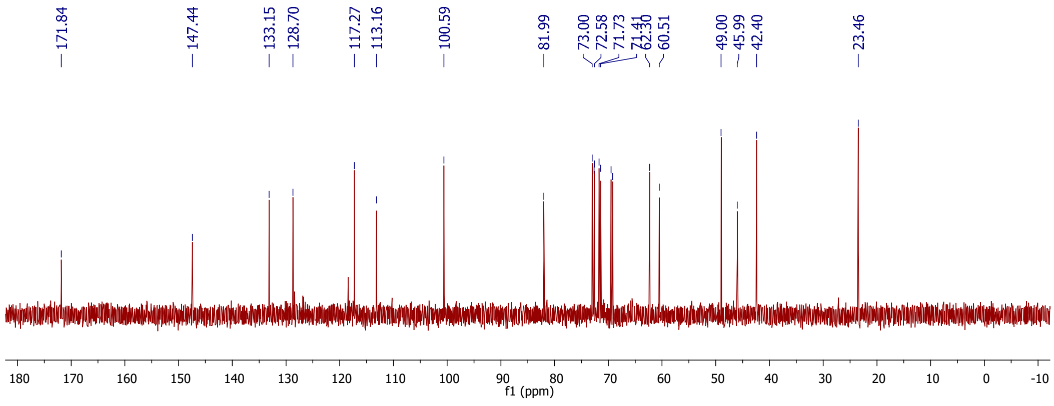

^{1}H and ^{13}C{^{1}H} NMR data of N-Acetyl-D-lactosamine TEAB conjugate (13)

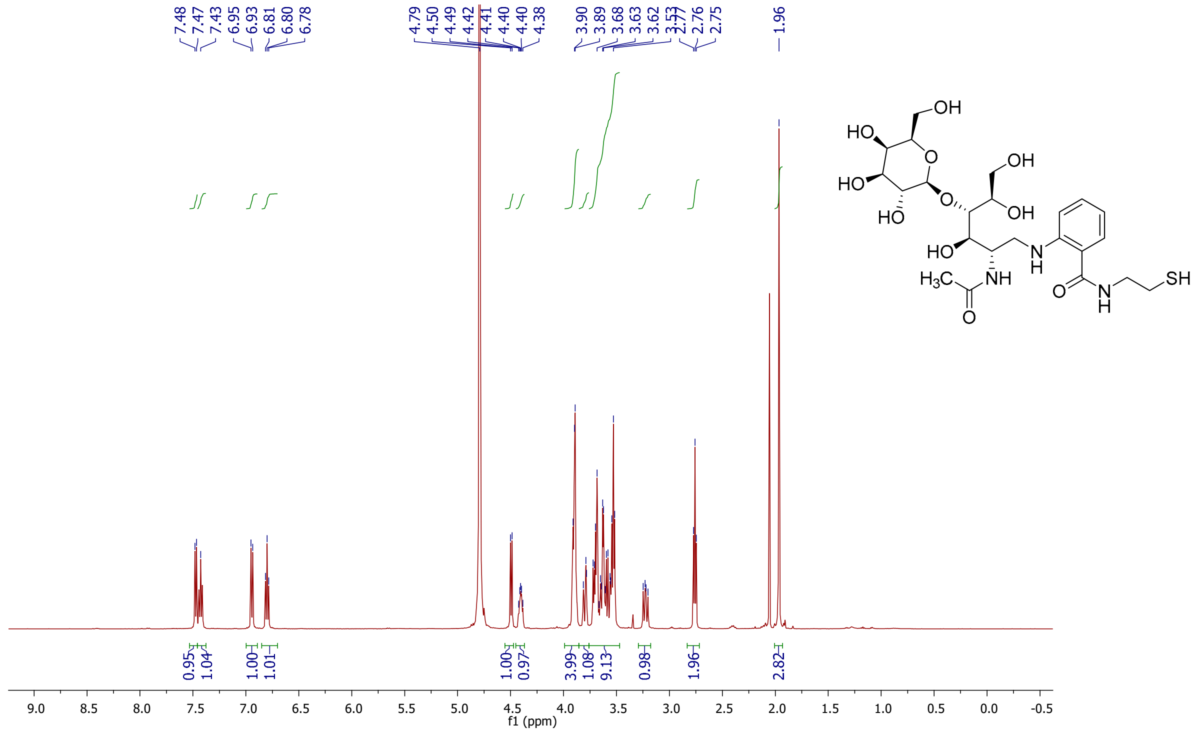

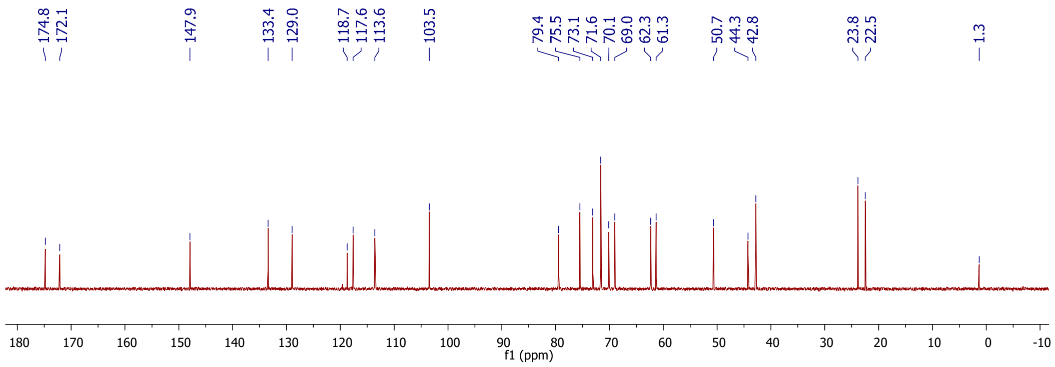

^{1}H and ^{13}C{^{1}H} NMR data of β-lactose-TEAB conjugate (14)

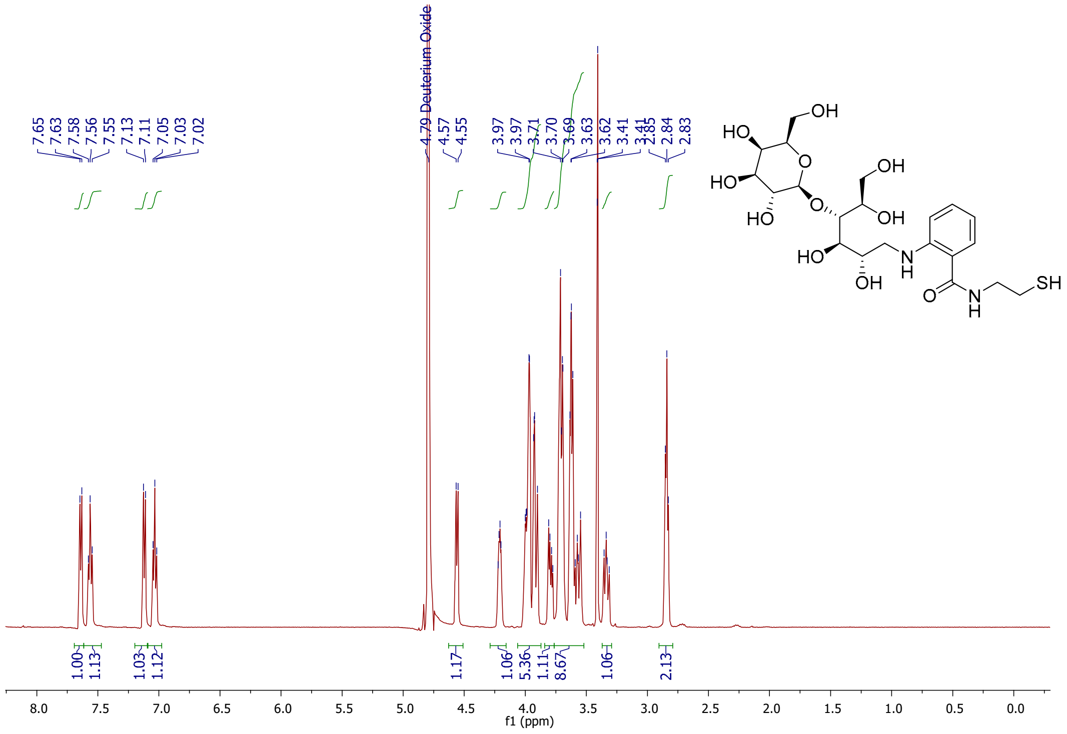

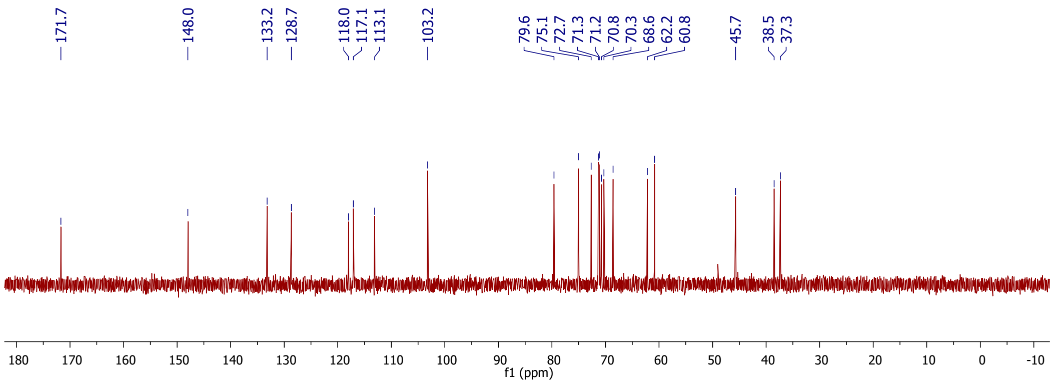

UV-Vis absorption and fluorescence data of lactose-TEAB conjugate (14)

Acquired in water; $\lambda_{max\ 1}$ 278 nm, $\lambda_{max\ 2}$ 326 nm $\lambda_{ex\ 2}$ 440 nm – 4700 M^{-1} cm^{-1} at 326 nm

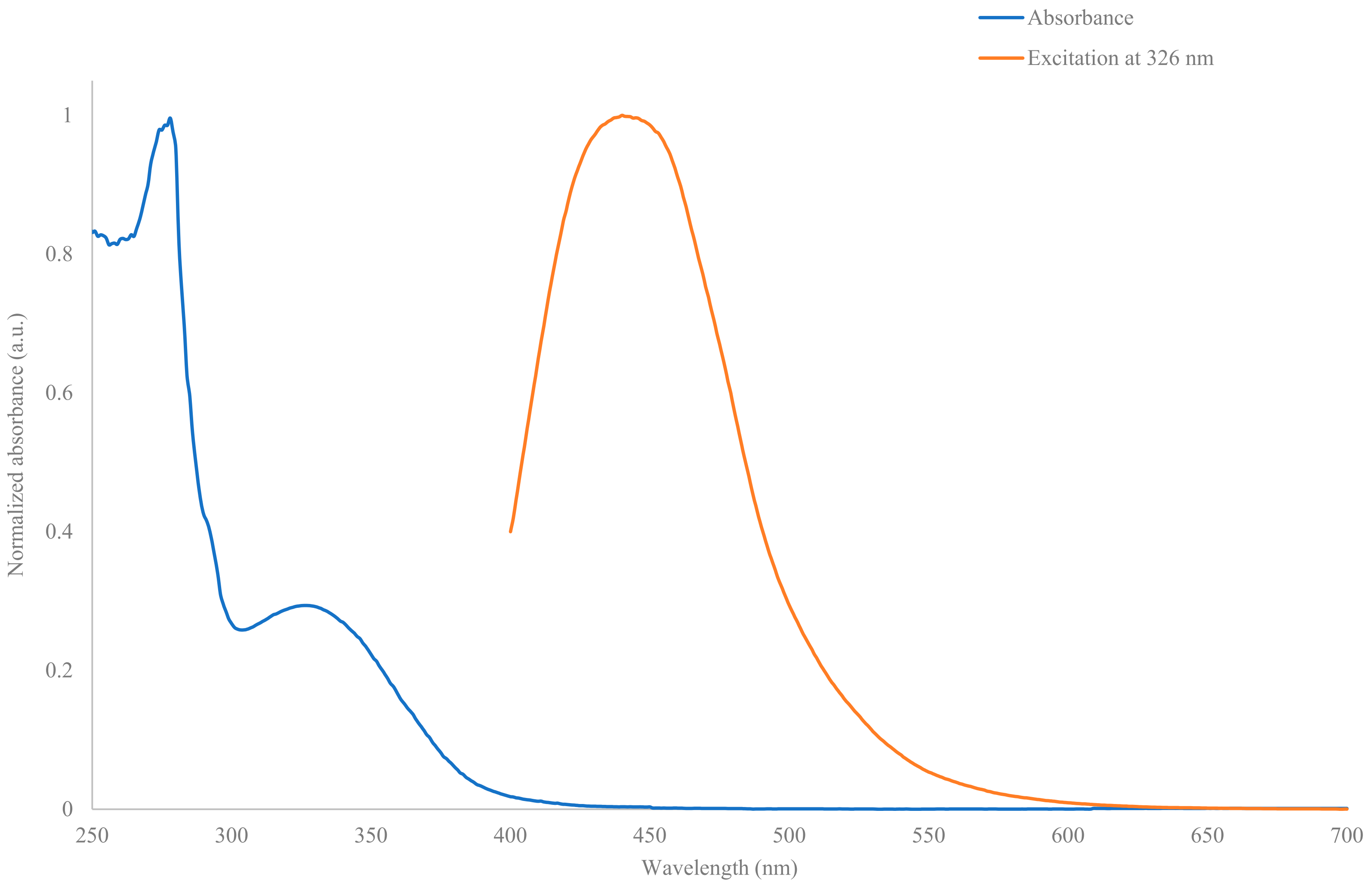

^{1}H and ^{13}C{^{1}H} NMR data of 1,3-α-1,6-α-D-mannotriose-TEAB conjugate (15)

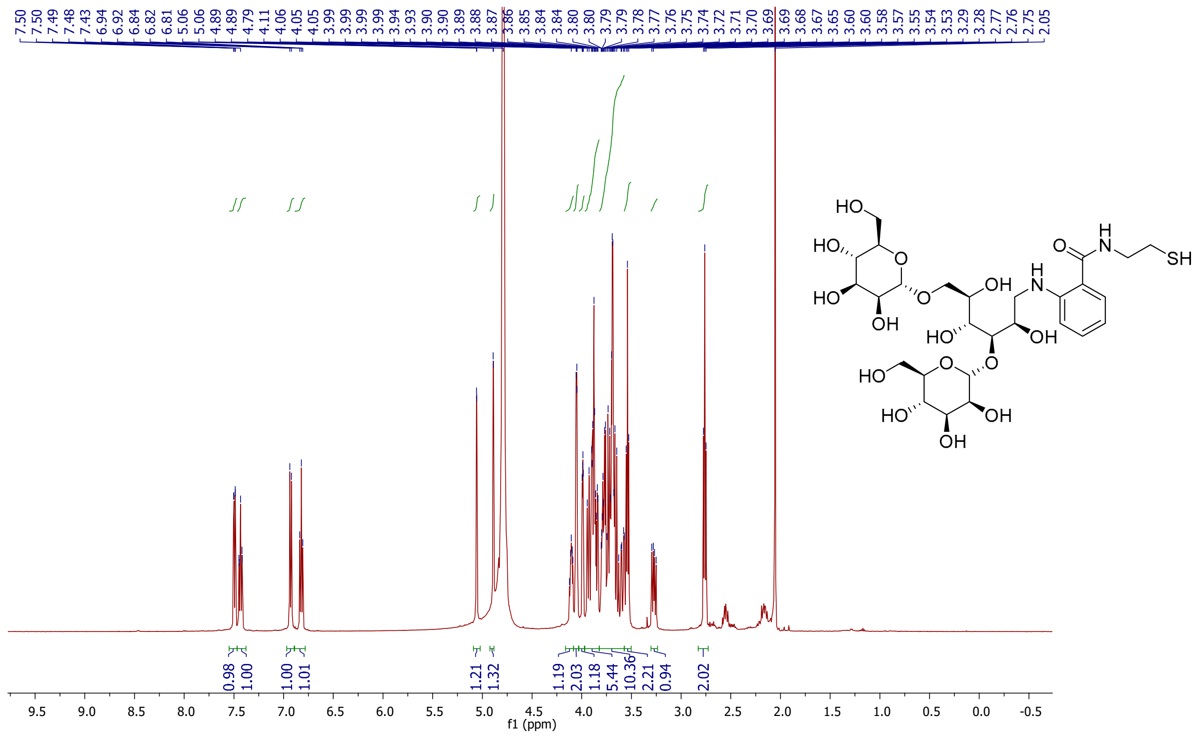

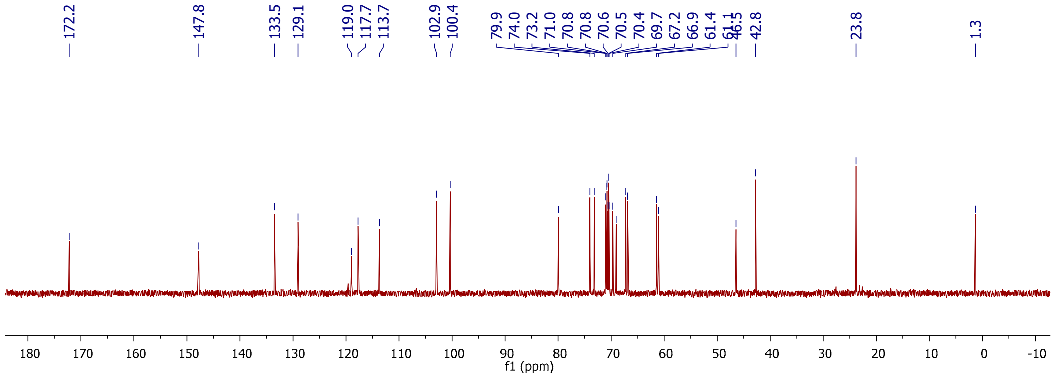

^{1}H and ^{13}C{^{1}H} NMR data of acarbose-TEAB (16)

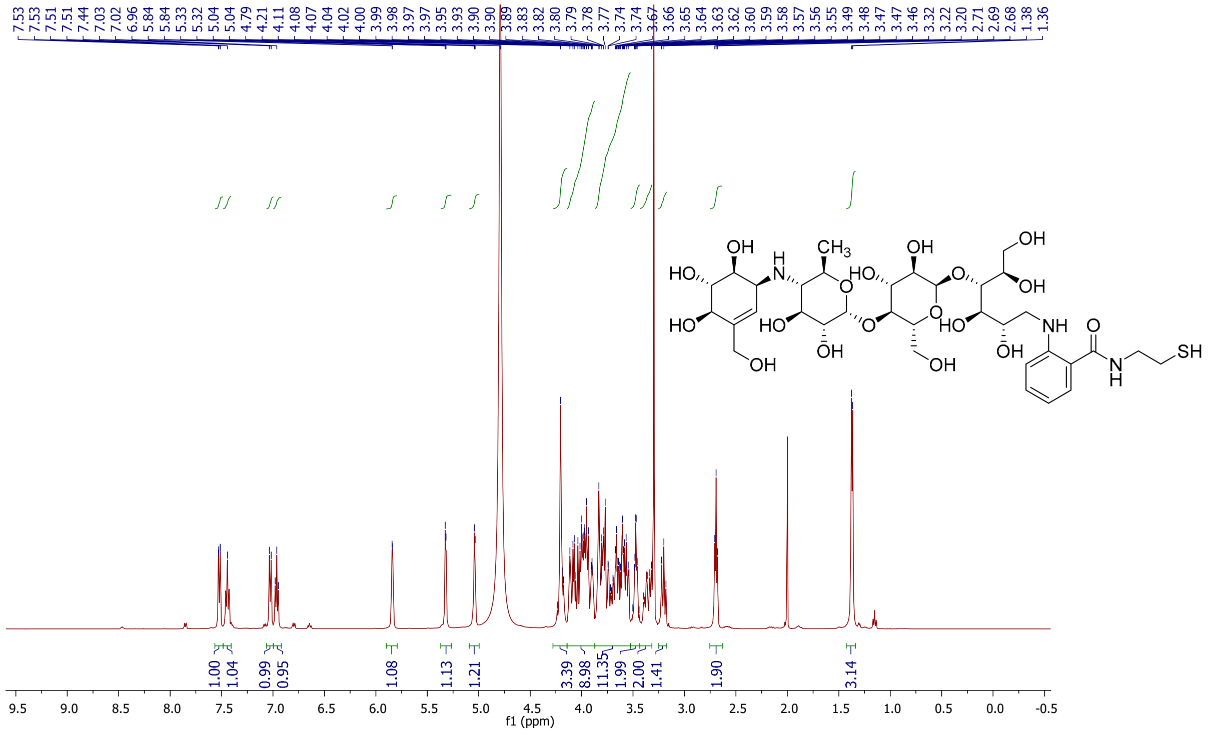

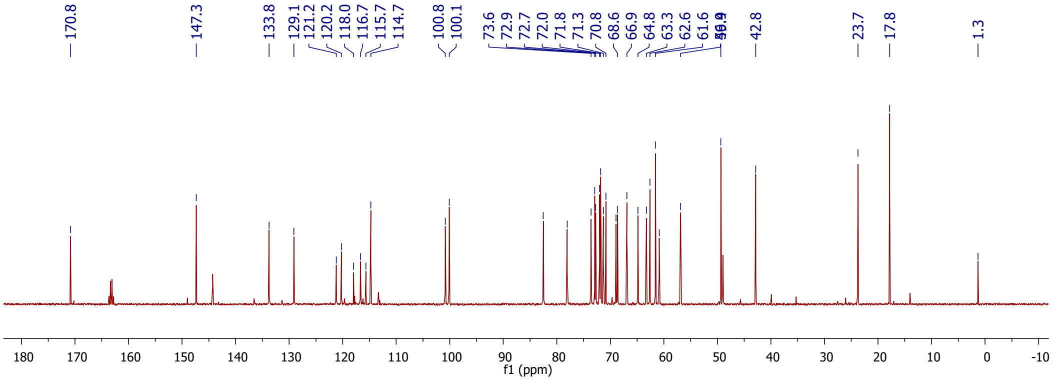

^{1}H and ^{13}C{^{1}H} NMR data of maltoheptaose-TEAB (17)

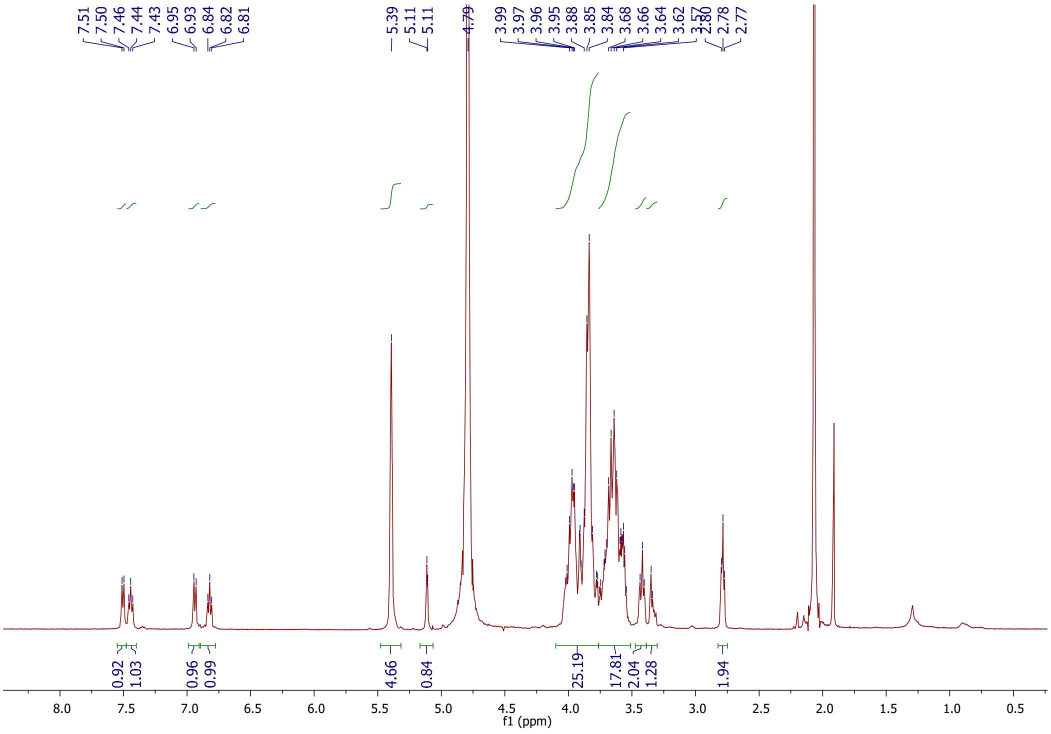

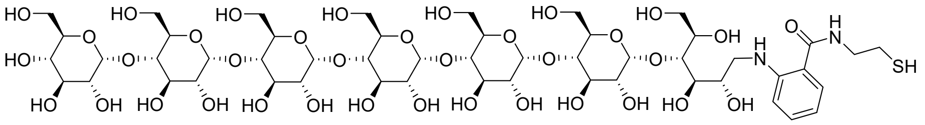

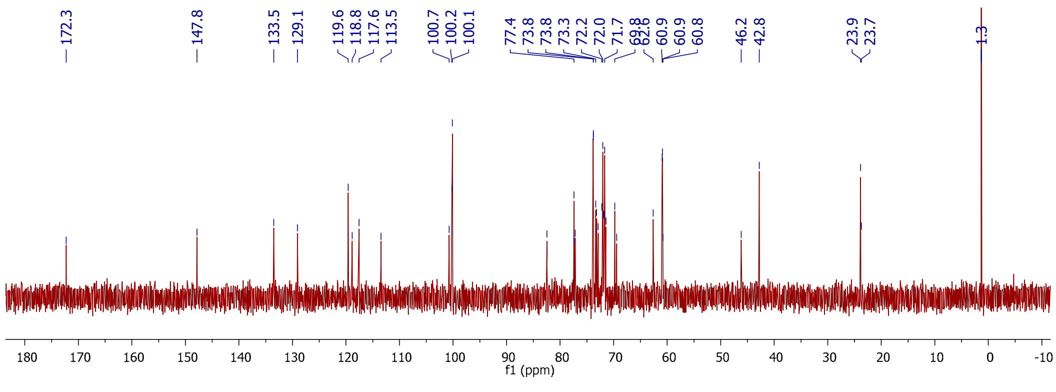

^{1}H and ^{13}C{^{1}H} NMR data of lactose-TEAB-maleimidoundecanoic acid (18)

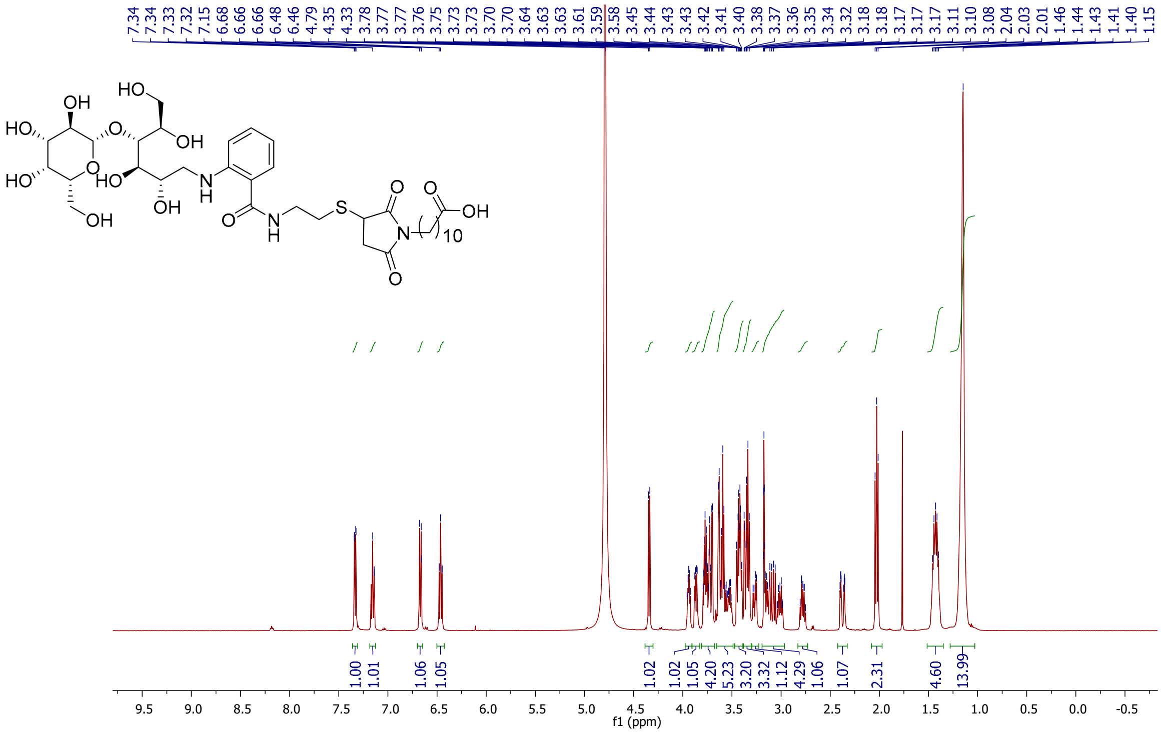

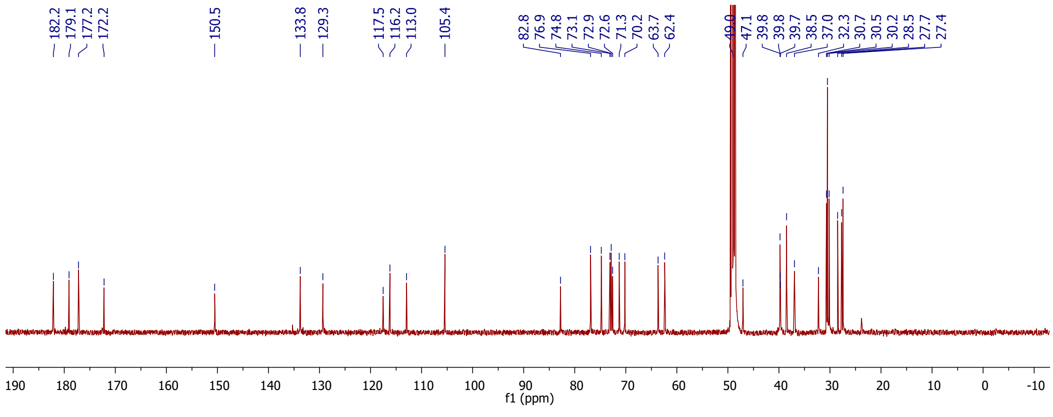

^{1}H, ^{13}C{^{1}H}, NOESY, HSQC NMR data of lactose-TEAB-noroborene dicarboxylic acid (19)

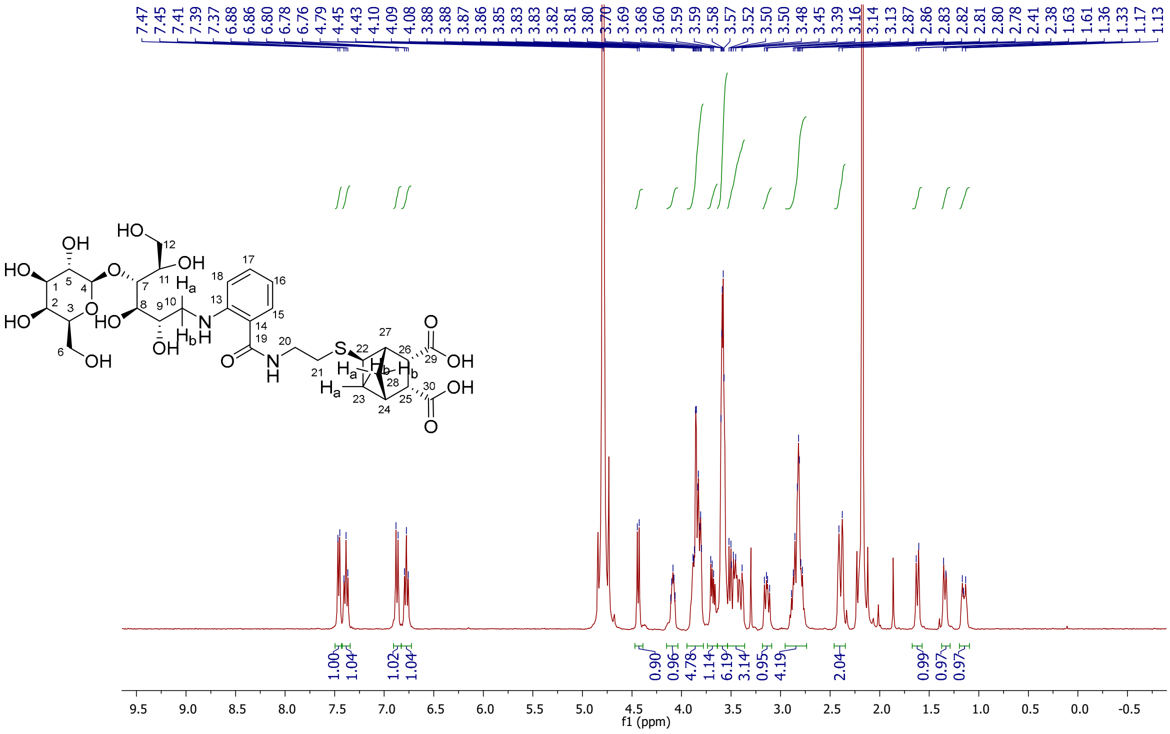

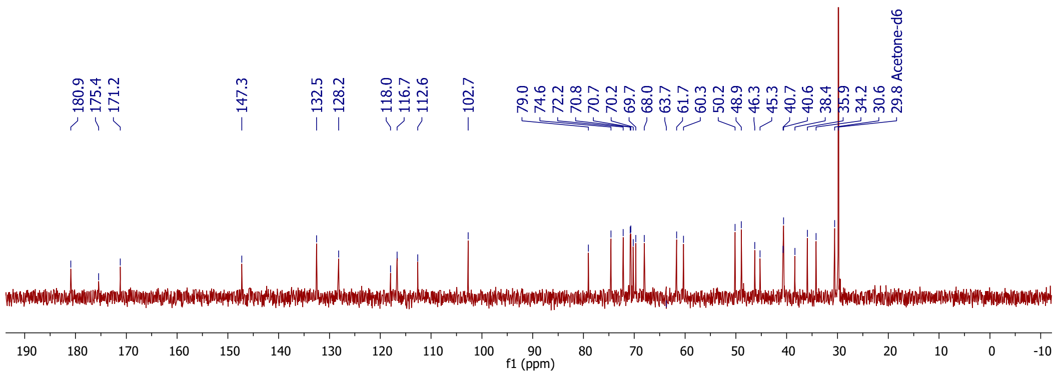

Phase-sensitive ge-2D NOESY with z-spoil of compound 19 – (NOESYGPPHPP)

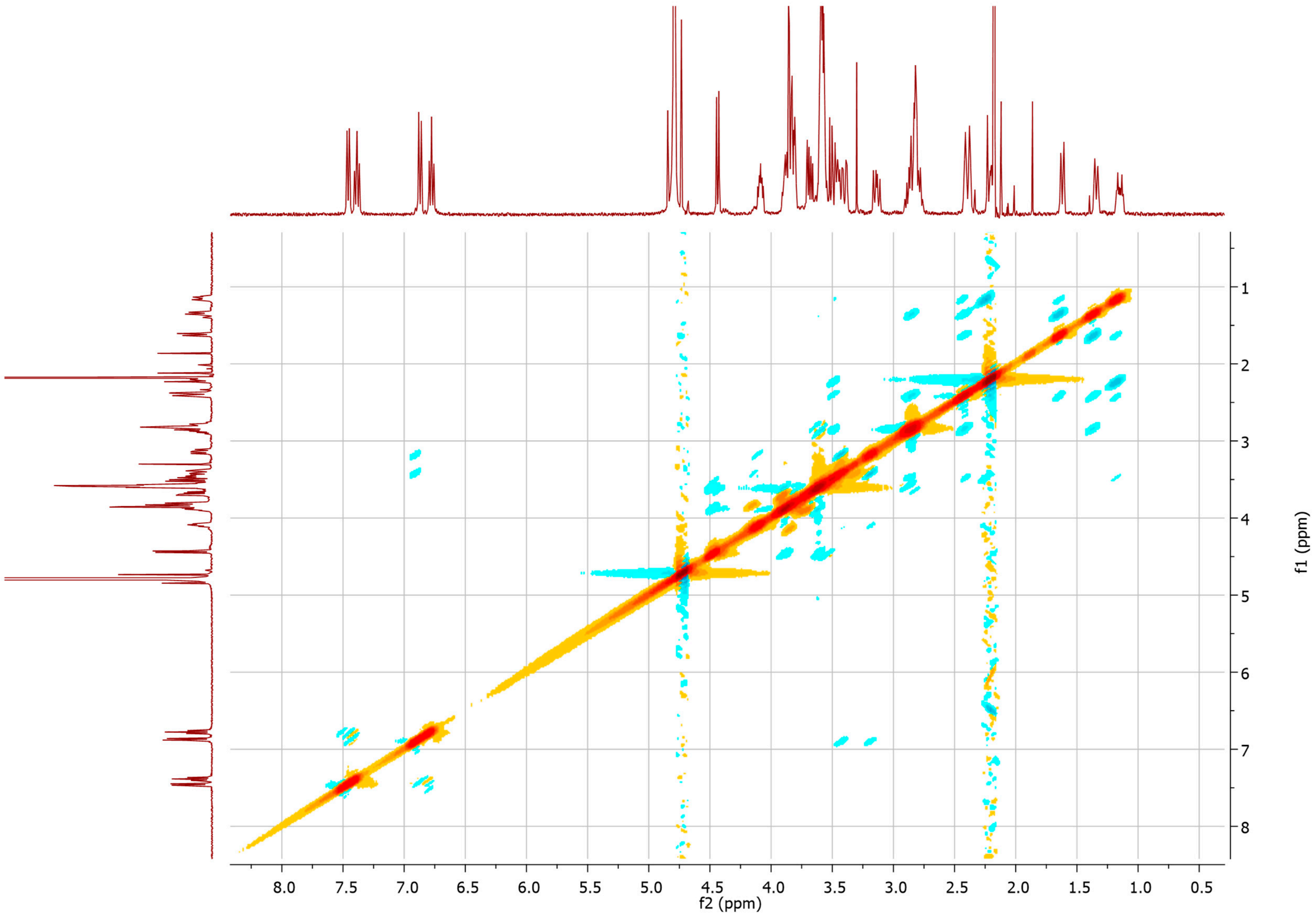

Phase-sensitive ge-2D HSQC using PEP and matched sweep adiabatic pulses for inversion and refocusing with gradients in back-inept of compound 19 – (HSQCEDETGPSISP2.3)

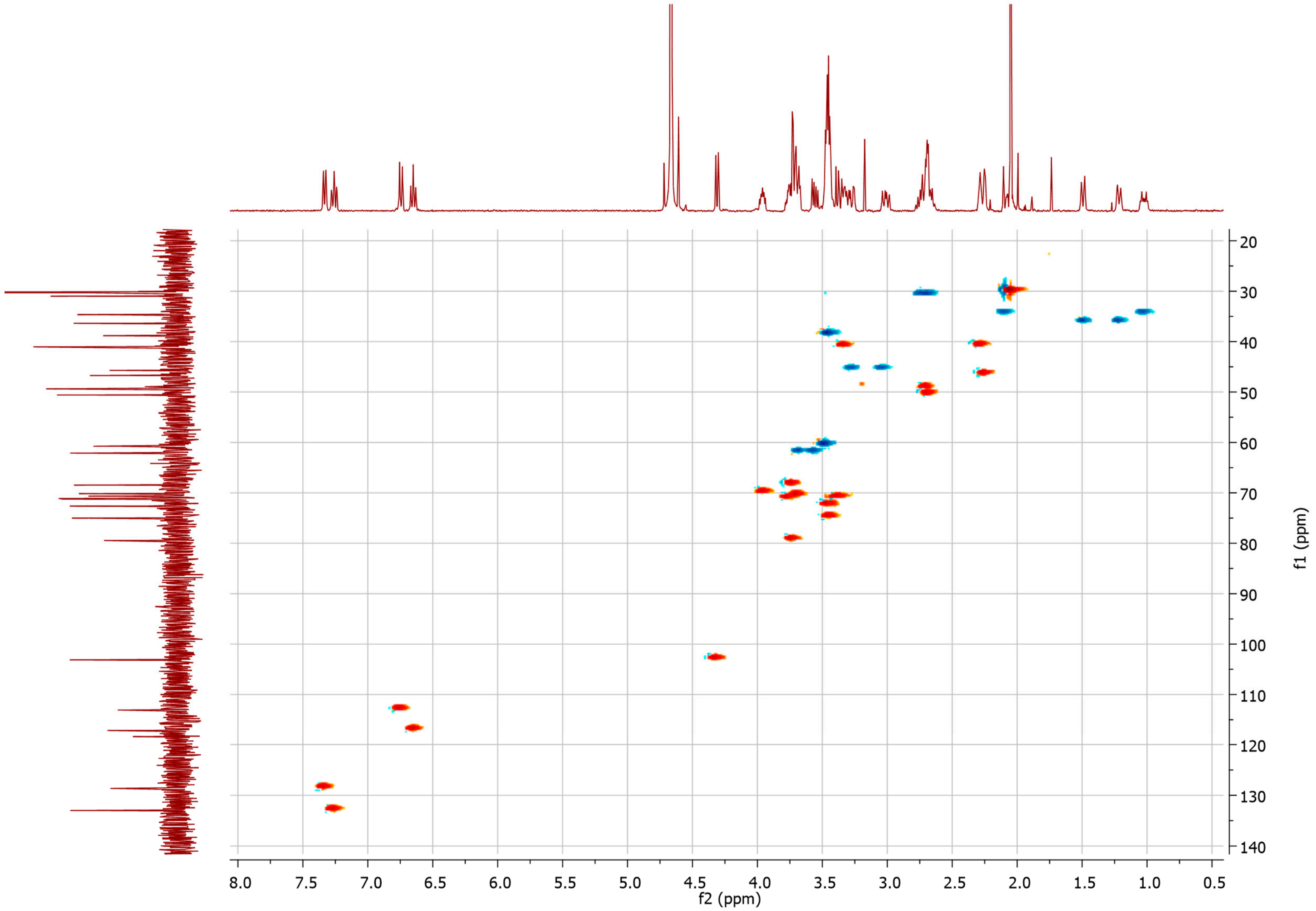

^{1}H and ^{13}C{^{1}H} NMR data of activated lactose-TEAB-disulfide pyridine (20, intermediate)

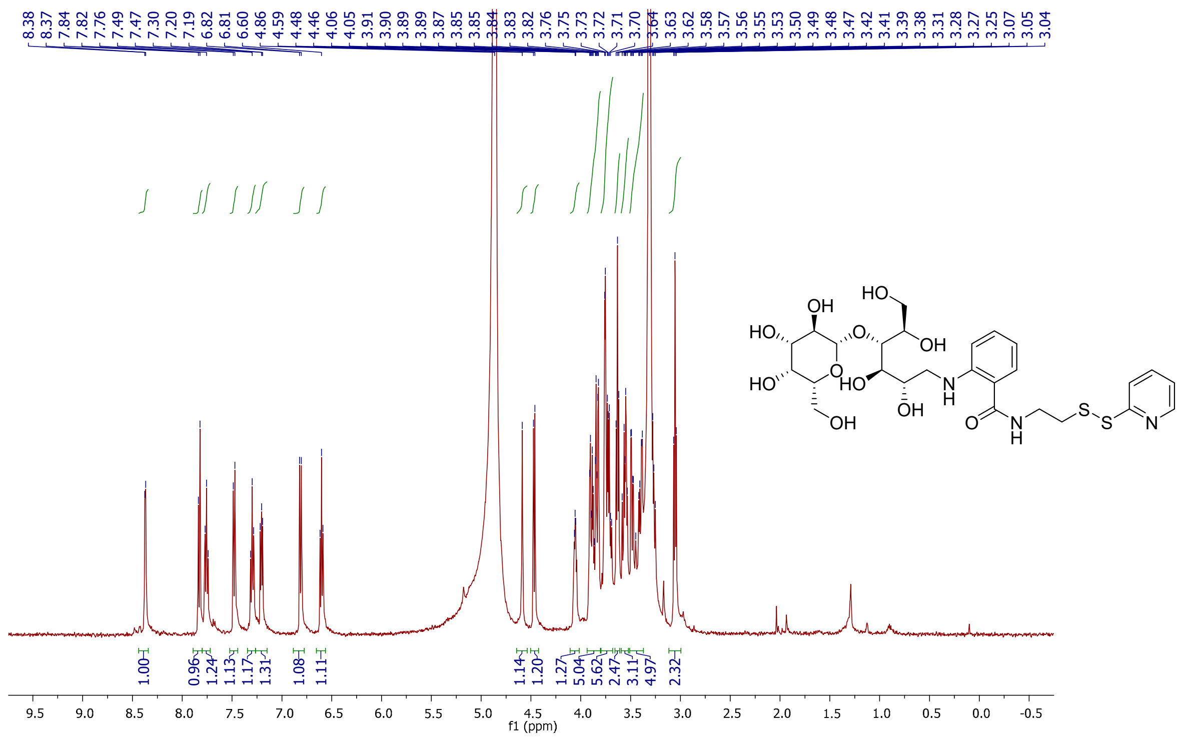

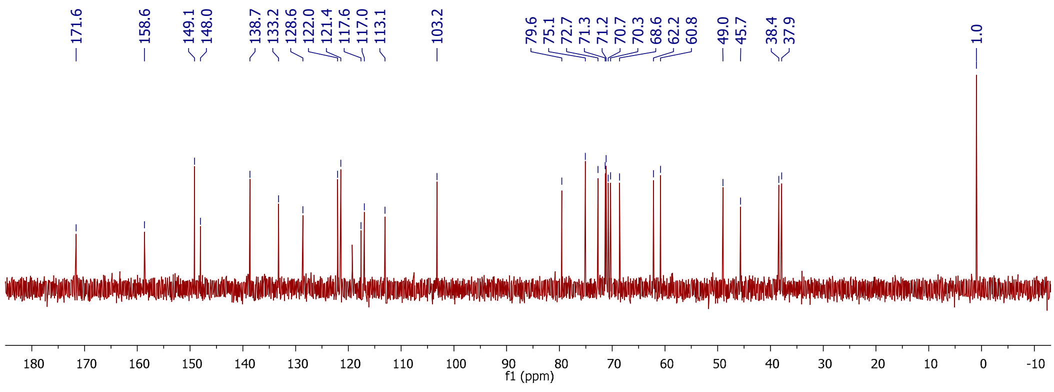

^{1}H and ^{13}C{^{1}H} NMR data of lactose-TEAB-difulfidehexane (20)

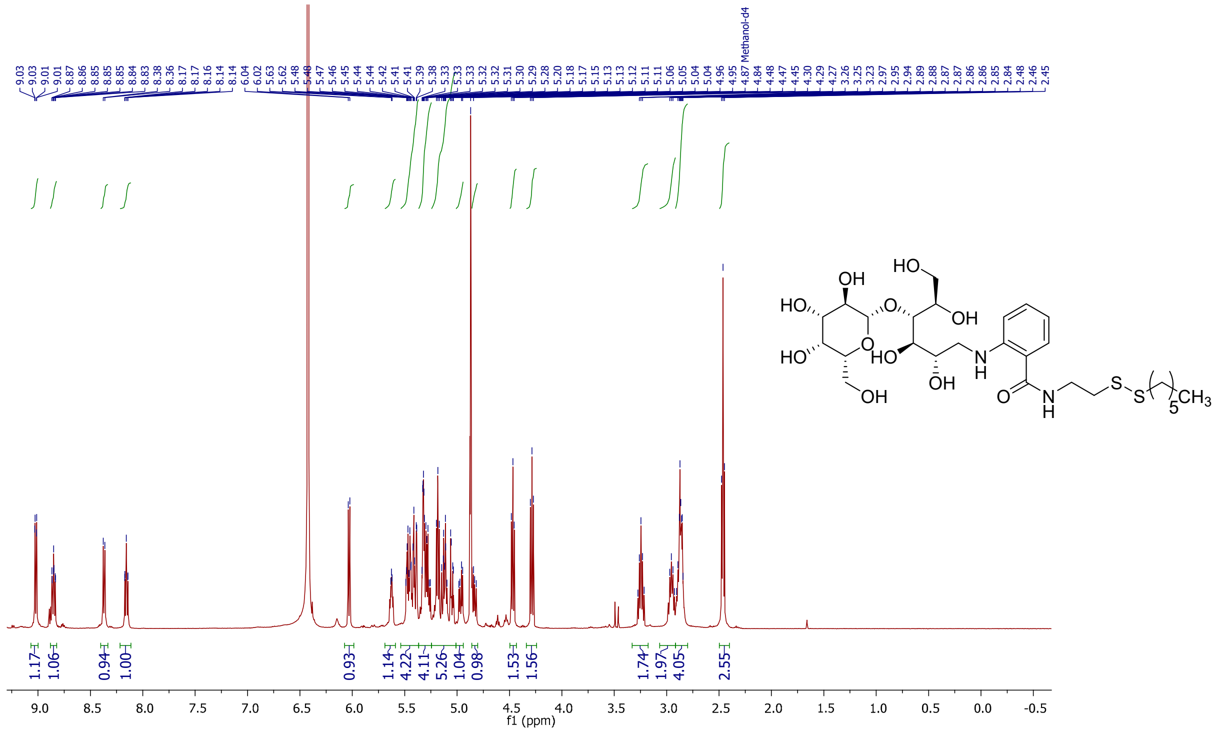

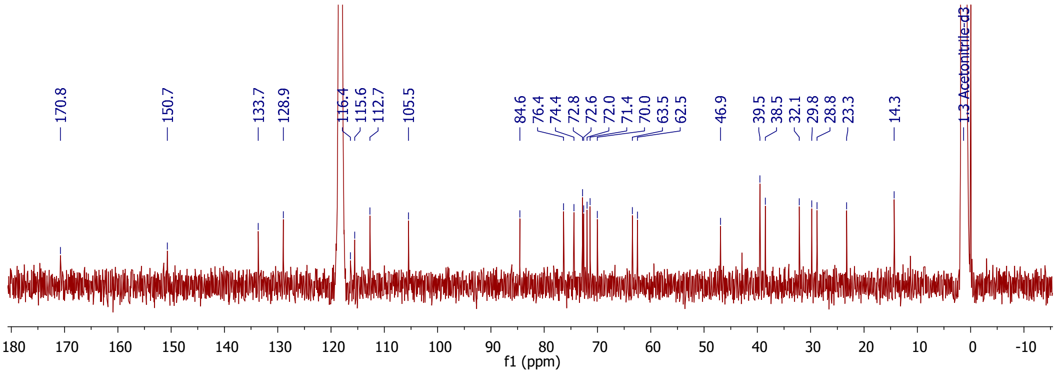

^{1}H and ^{13}C{^{1}H} NMR data of lactose-TEAB-acetamide (21)

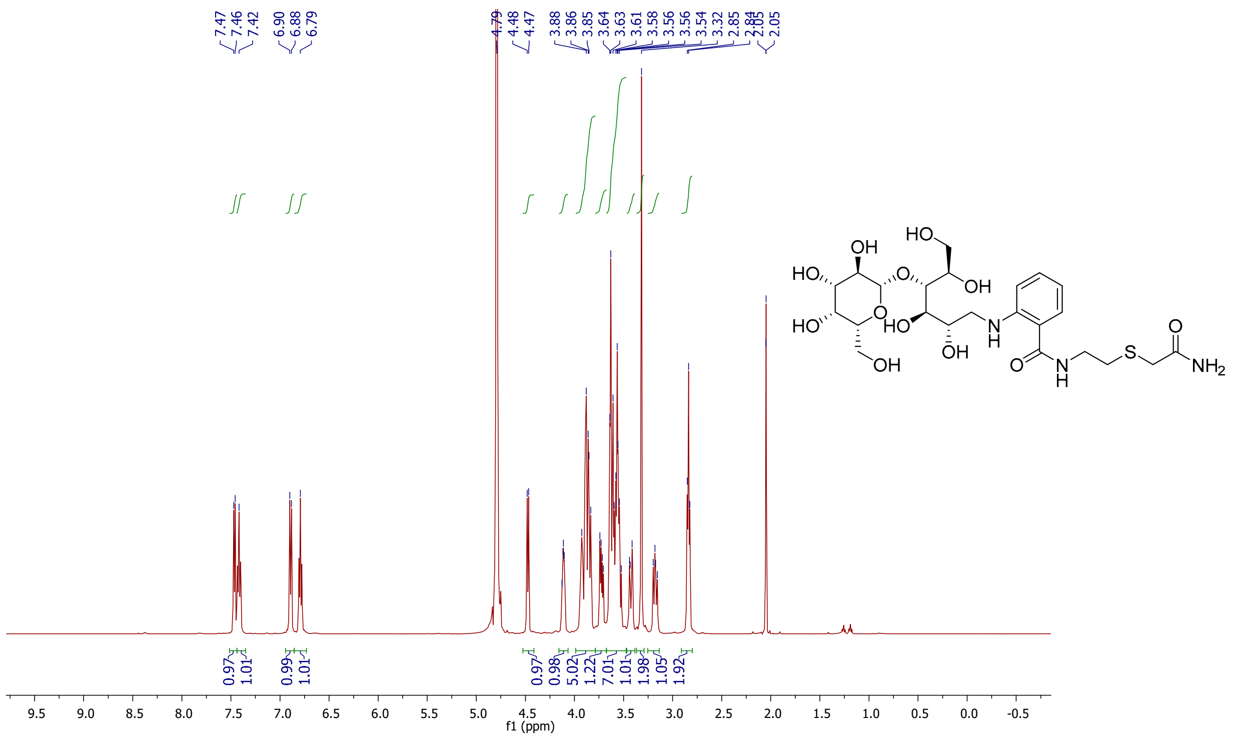

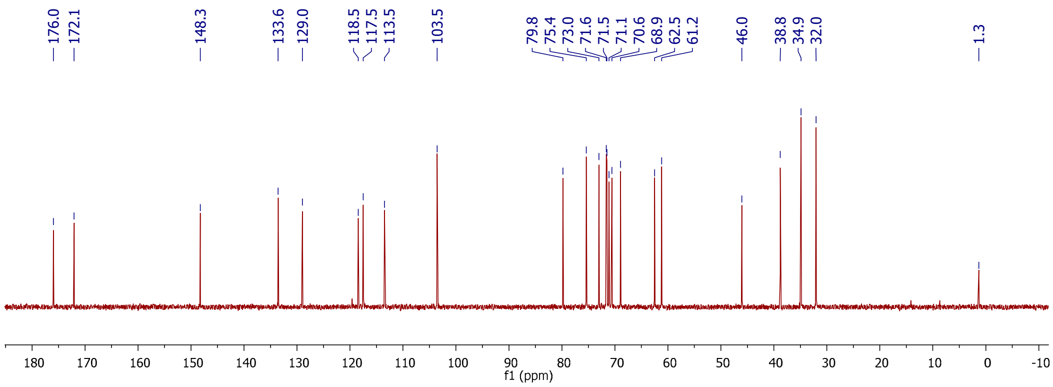

^{1}H and ^{13}C{^{1}H} NMR data of lactose-TEAB-BOC-L-Leucine thioester (22)

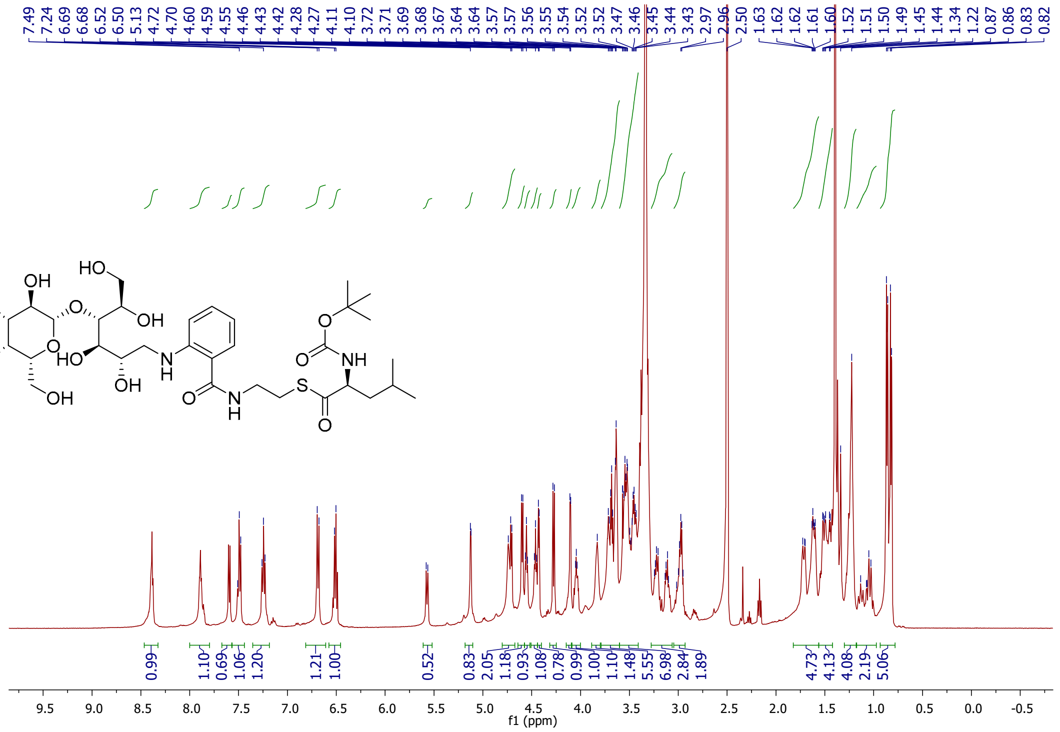

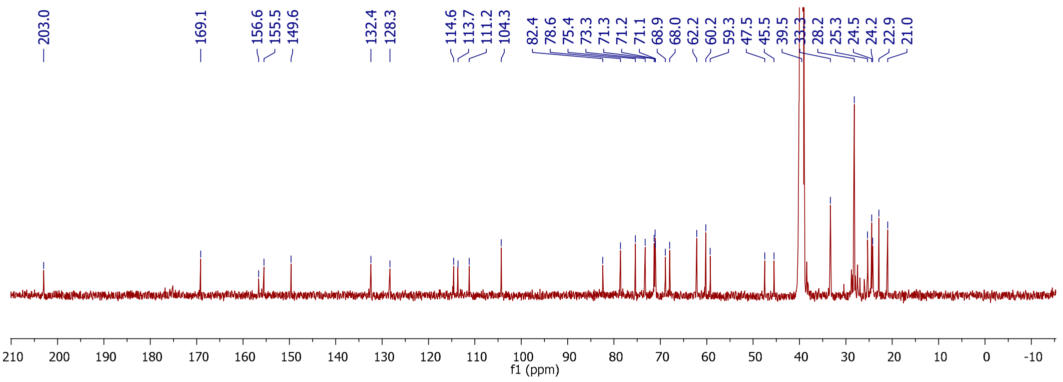

^{1}H and ^{13}C{^{1}H} NMR data of lactose-TEAB-butyne (23)

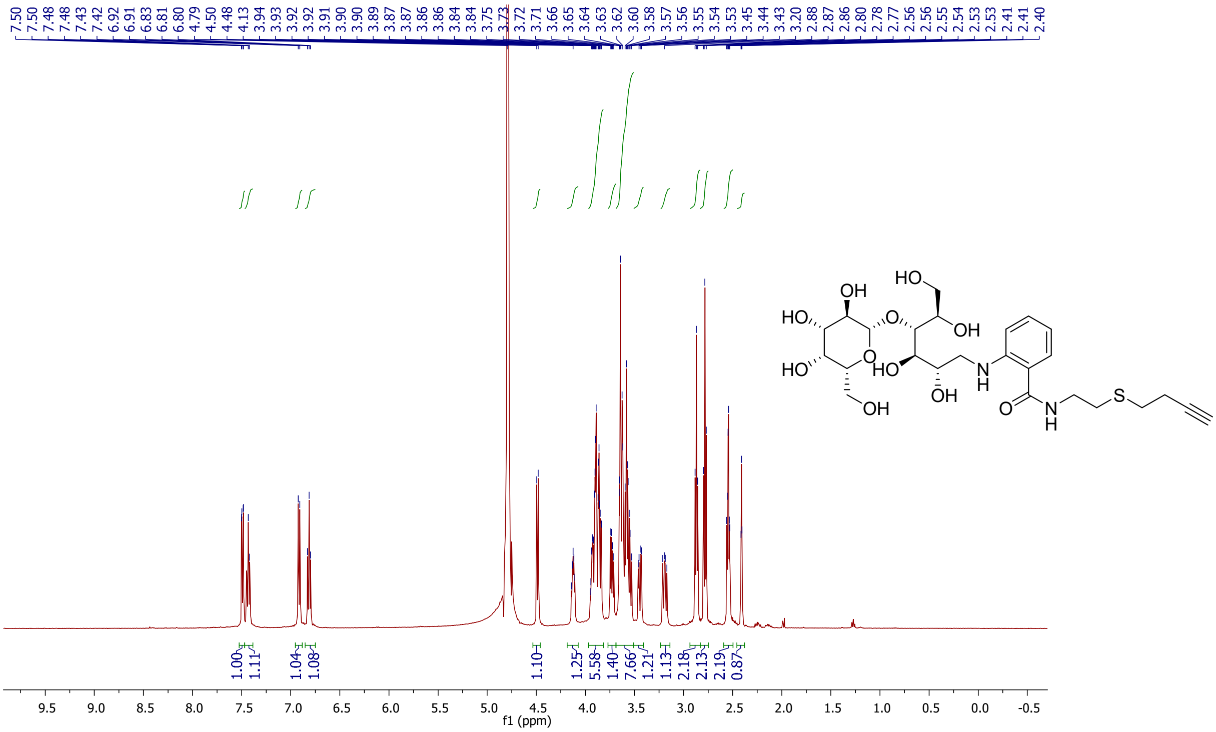

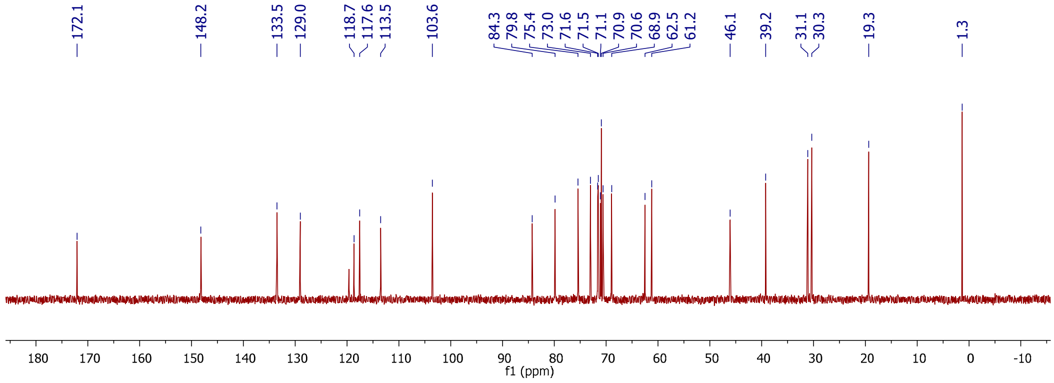

^{1}H and ^{13}C{^{1}H} NMR data of lactose-TEAB-propanediol (24)

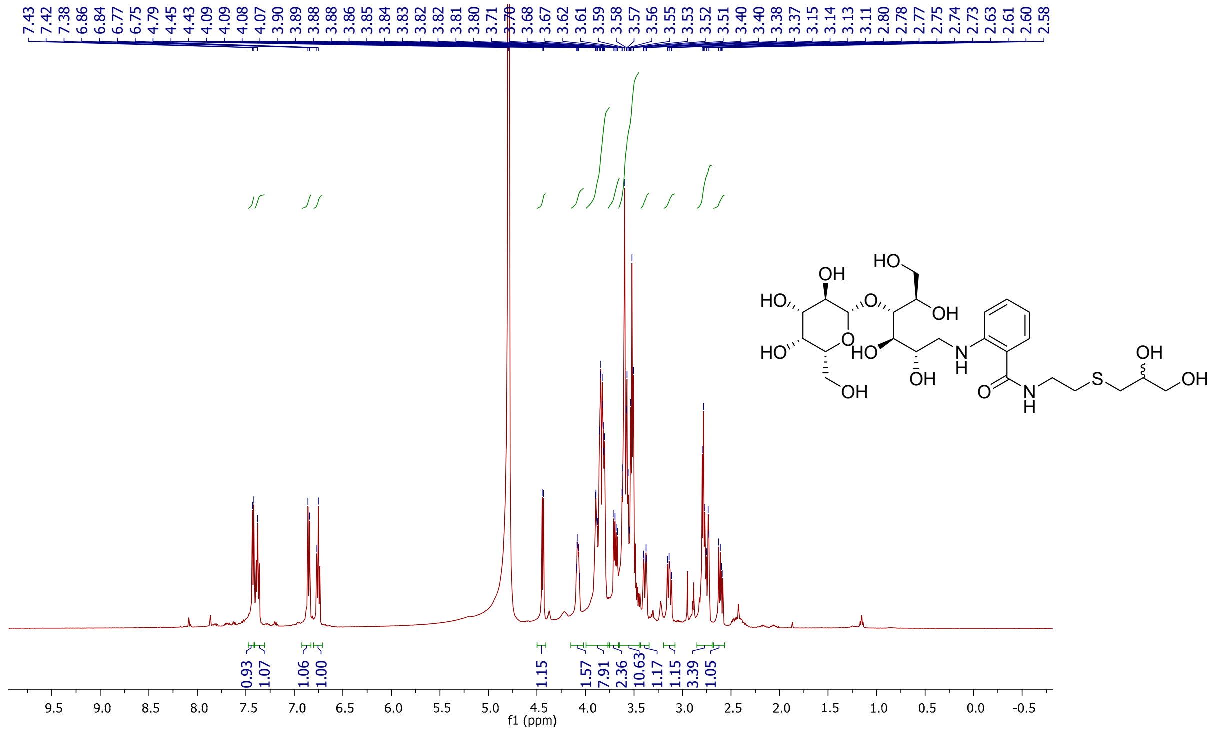

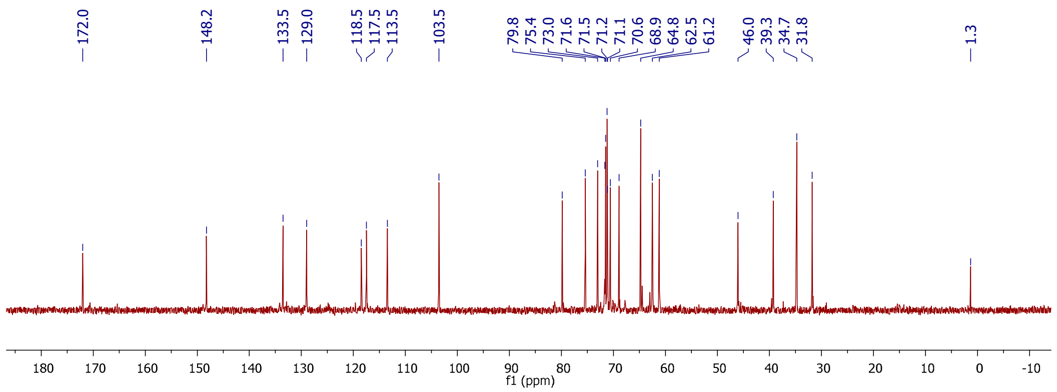

Spectroscopy data of lactose-TEAB-bismaleimide-BSA (26)

Calculated mass of 26: 66,429 Da + 522 Da + 308 Da = 67,259 Da ~ 67.26 KDa

(BSA Protein) + Lactose-TEAB (**14**) + bismaleimide (**25**)

MALDI (Base Peak = 67,245 Da ~ 67.25 KDa)

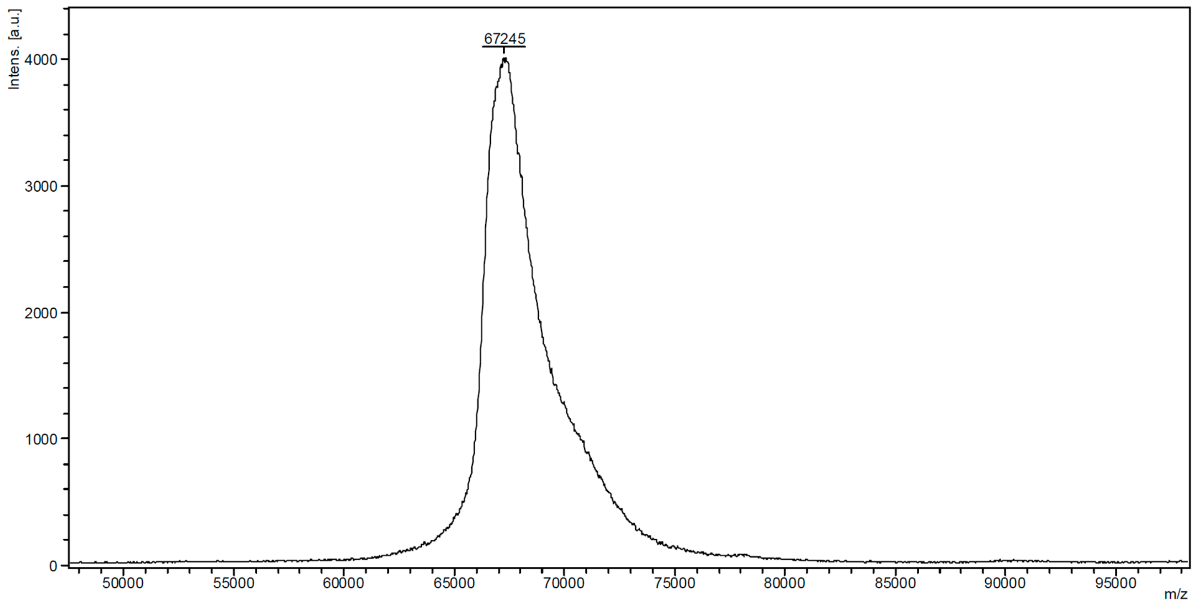

UV-Vis quantification of activated Sili-MetSH (27)

Calibration curve of 2-mercaptopyridine in DMF

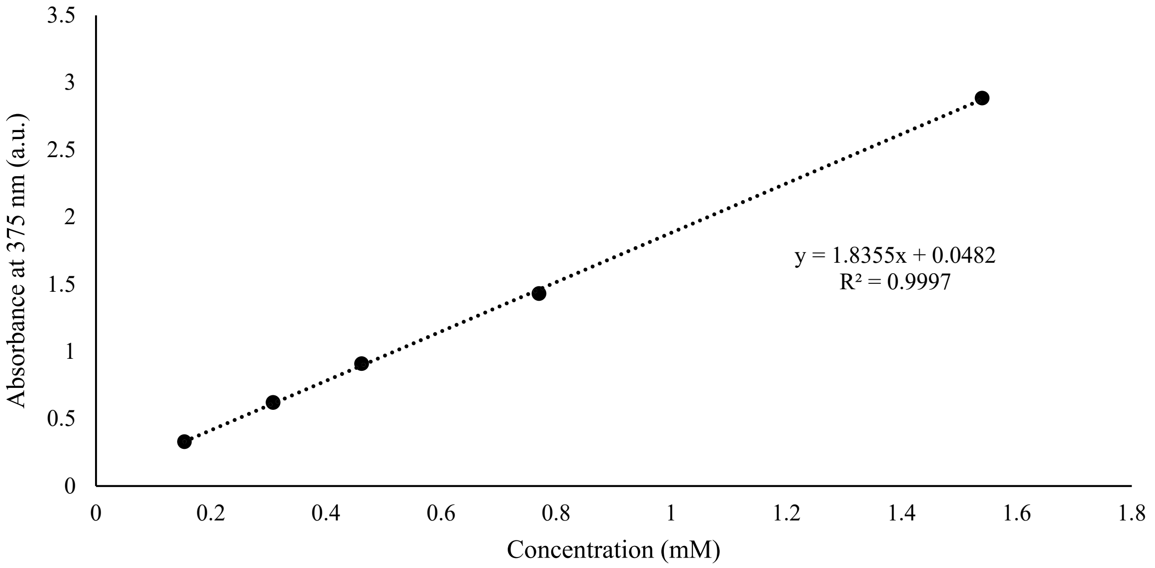

Modification of compound 27 based on 2-thiopyrdine produced

Protocol 1: SO_2Cl_2 (x eq.) and 2-thiopyridine (2 eq.) were dissolved in dry DCM (3 mL) was poured into a vial to which SiliMetSH (500 mg, 0.77 mmol SH content from supplier, 1 eq.) was slowly added to the fuming mixture over a period of 5 minutes. Once completed the suspension was stirred for 10 minutes and then placed onto a rotary evaporator at 45 ˚C, reduced pressure was applied until complete removal of solvent. The dried solid was then re-dissolved in dry DMF and UV-Vis absorption taken of the diluted sample.

Protocol 2: SO_2Cl_2 (10 eq.) was dispensed into a vial to which SiliMetSH (500 mg, 0.77 mmol SH content from supplier, 1 eq.) was slowly added to the fuming mixture over a period of 1 minute. Once completed the silica gel was placed onto a rotary evaporator and heated to 45 ˚C, reduced pressure was applied until complete removal of any excess SO_2Cl_2. Separately 2-thiopyridine (2 eq.) was dissolved in dry DCM (3 mL) and slowly added to the chlorinated silica. After stirring the suspension for 10 minutes, the suspension was then concentrated and re-dissolved in dry DMF and UV-Vis absorption taken of a diluted sample.

Reaction conditions varied	Activated SH (mmol)	Remaining SH (mmol)
Protocol 1 - SO_2Cl_2 (10 eq.) TP (2 eq.)	0.355	0.415
Protocol 1 - SO_2Cl_2 (25 eq.) TP (2 eq.)	0.277	0.493
Protocol 2	0.997	-0.227

TP = 2-Thiopyridine

Column format of compound (27) for thiol catchment

Application of compound **27** in a column format was investigated where the initial filtration step was applied with similar loadings and concentrations relative to the described optimized stirring format, a flow rate of ~1 mL per 3 minutes was used with **27** (250 mg) equilibrated in water within a cotton plug pipette. Purified glycan (**14**, 40 mg) was loaded into the column dissolved in water (2 mL), the column was then further washed with water (5 mL), methanol (5 mL) and acetone (5 mL) where the resulting filtrate was then concentrated under reduced pressure. It was found that the initial filtration had consumed 19 mg, 41% (approximated by mass loss of dried filtrate 21 mg) of the derivatized glycan with the remaining filtrate containing 43:57 (S-H:S-S) determined by NMR quantification of the CH_2 triplet at 3.00 ppm (S-S) and 2.70 ppm (S-H) not accounting for the 2-thiopyridine in the resulting mass.

^{1}H, ^{13}C{^{1}H} NMR data of compound (28)

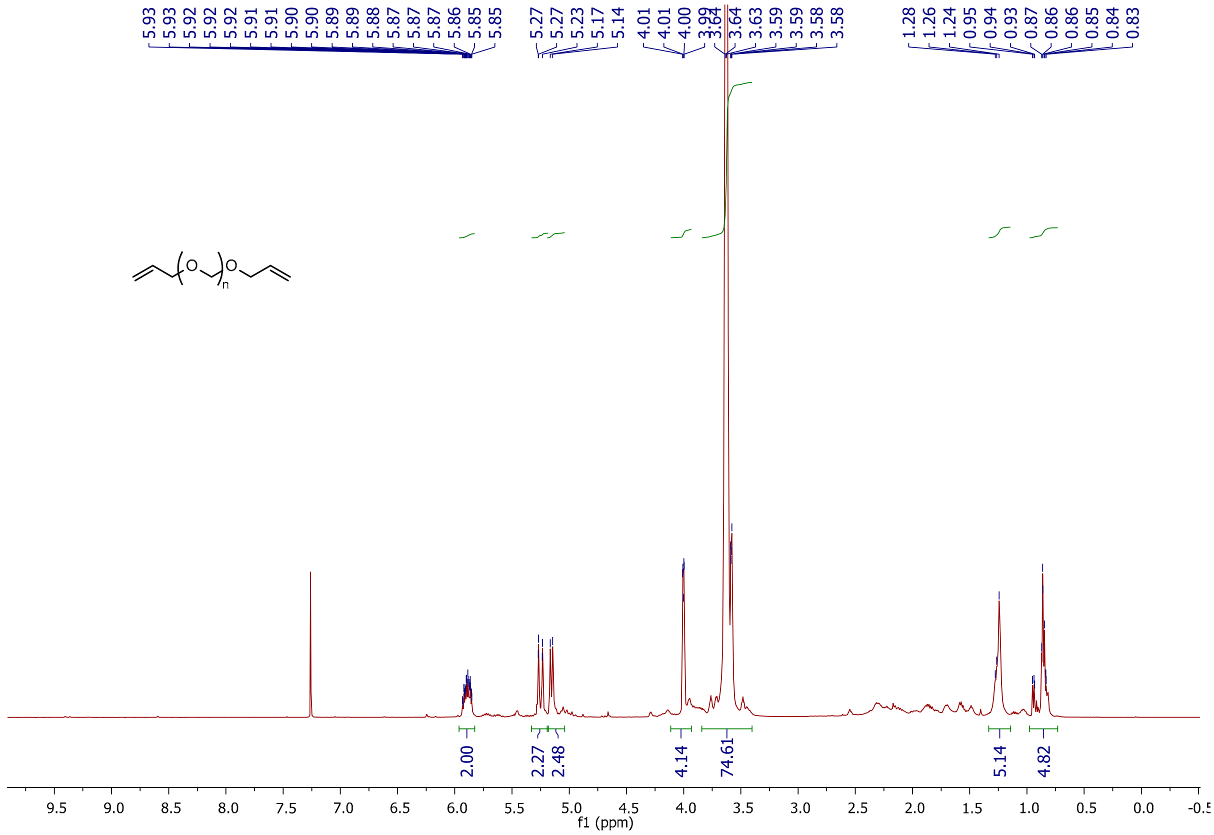

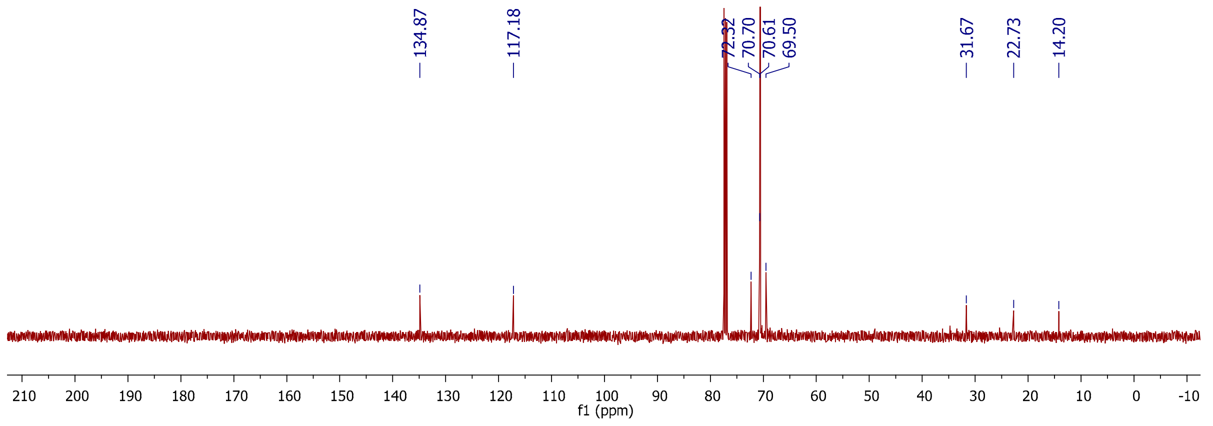

UV-Vis optimization of allylated PEG microscope slides

Adjusting time of PEGylation – 10 w/v% PEG, 5 w/v% AIBN in toluene at varying time.

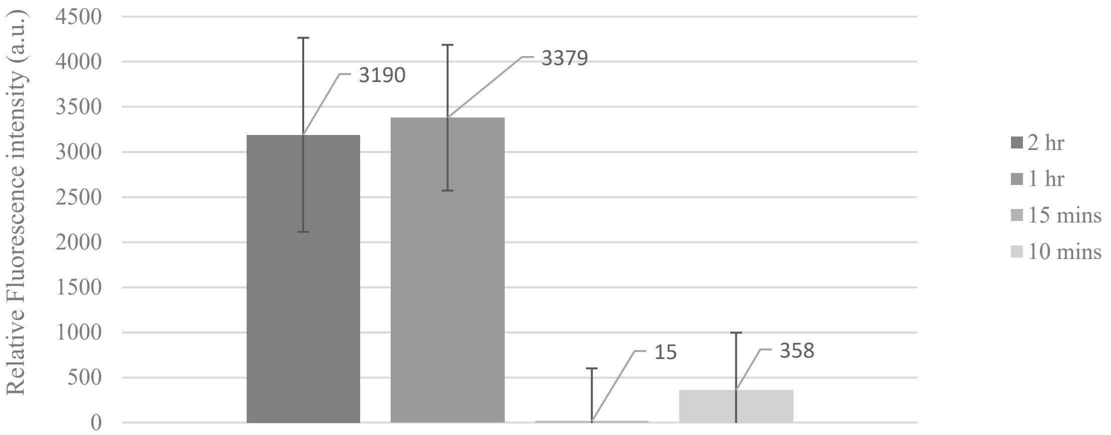

Adjusting concentrations of initiator and PEG - All samples reacted for 1 hr with stated solution in toluene. Error bars are shown as one standard deviation between triplcate sample spots on the same slide.

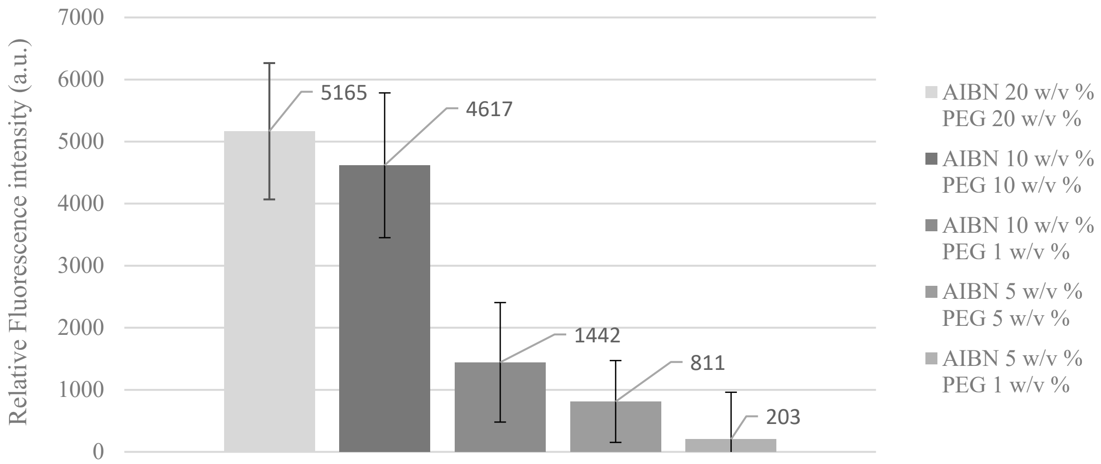

All samples run in triplicate with 3 sample wells on a 10 mm x 25 mm cut from the same prepared slide batch, 10 µL aliquots were dispensed onto each well. Fluorescence was measured with 50 flashes per read of the wells analyzed with 385 nm excitation and 490 nm emission, reading from the underneath the plate. Blank is defined as a non-reacted (non-pegylated) region on a thiol coated glass slide measured at the same time of the other readings with the same settings defined, all samples have been made relative to the blank region to determine significance. All regions were thiol-ene clicked with the optimized conditions: lamp 22.41 mW/cm^2 for 1 minute with glycan **14** (10 mM), LAP (1 w/v%). The slide (25 mm x 75 mm) was drop casted with 1 mL of the stated solution for the stated time at 100 ◦C in an air oven. Error bars are shown as one standard deviation between triplcate sample spots on the same slide.

UV-Vis optimization of glycosylated PEG microscope slides

Adjusting irradiation time - Lamp 22.41 mW/cm2 for x minutes with glycan **14** (10 mM) and LAP (1 w/v%)

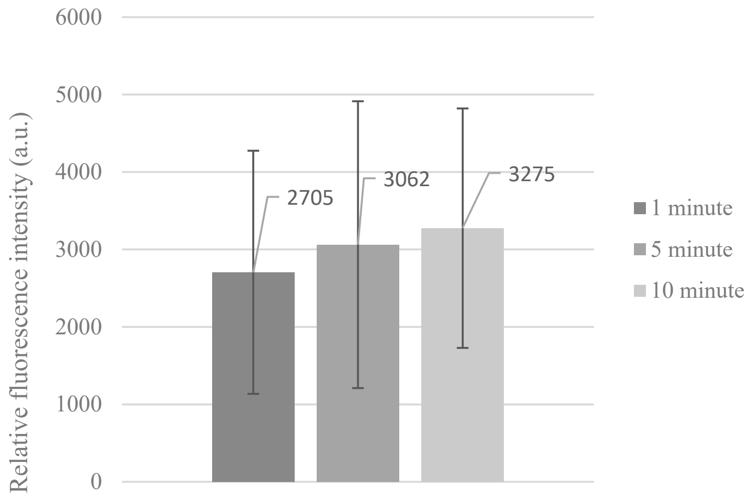

Adjusting lamp power - Lamp x mW/cm^2 for 5 minutes with glycan **14** (10 mM) and LAP (1 w/v%). Error bars are shown as one standard deviation between triplcate sample spots on the same slide.

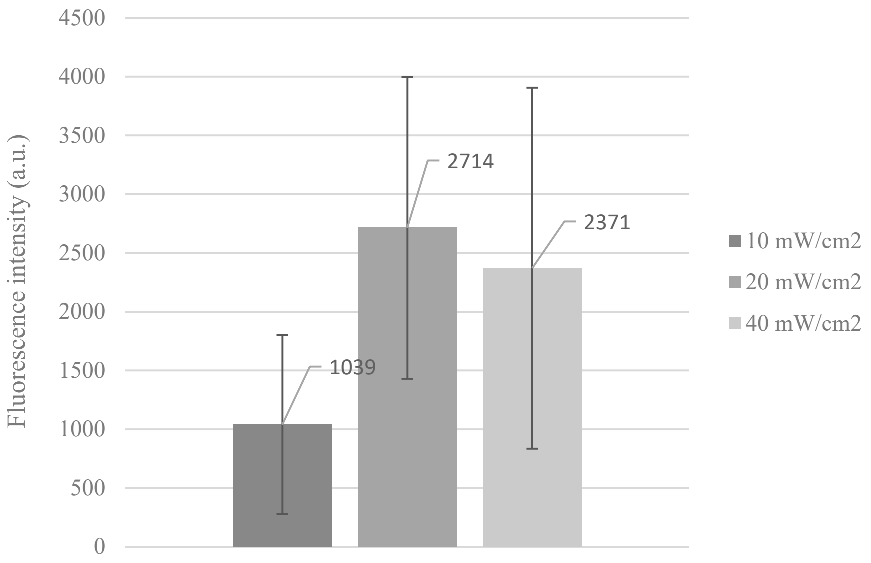

Additionally, 2-Hydroxy-4′-(2-hydroxyethoxy)-2-methylpropiophenone (I2959) was investigated as a photo initiator however efficiency of initiation was found to be significantly lower compared to LAP, requiring orders of magnitude higher concentrations to achieve observable fluorescent spots. Error bars are shown as one standard deviation between triplcate sample spots on the same slide.

Adjusting LAP - Lamp 22.41 mW/cm^2 for 5 minutes with glycan **14** (10 mM), LAP (x w/v%)

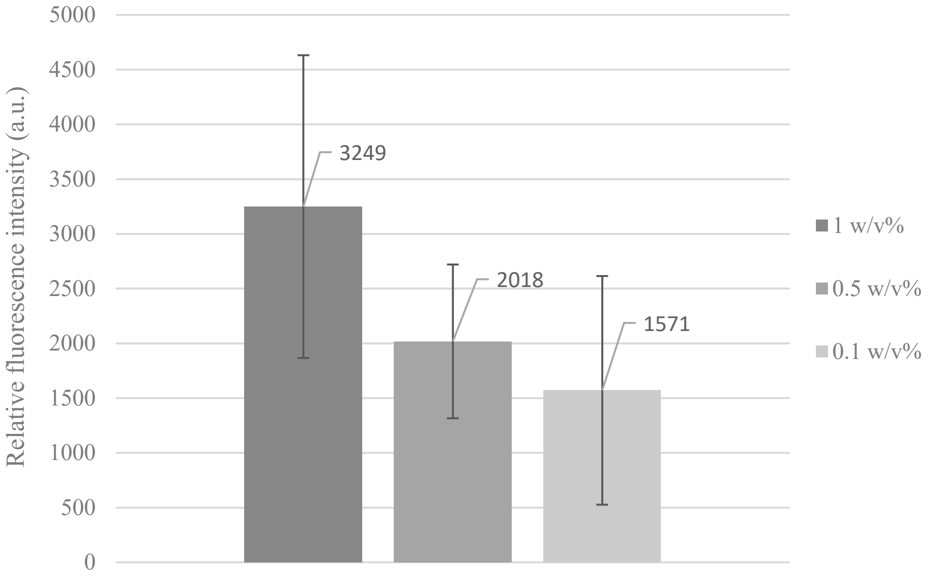

Adjusting glycan concentration - Lamp 22.41 mW/cm^2 for 5 minutes with glycan **14** (x mM), LAP (1 w/v%). Error bars are shown as one standard deviation between triplcate sample spots on the same slide.

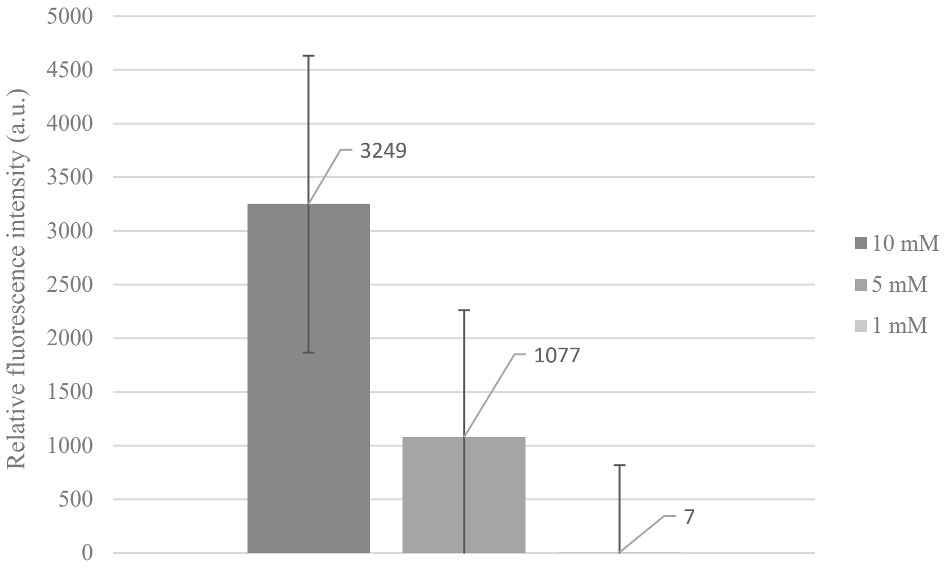

All samples run in triplicate with 3 sample wells on a 10 mm x 25 mm cut from the same prepared slide batch, 10 µL aliquots were dispensed onto each well. Fluorescence was measured with 50 flashes per read of the wells analyzed with 385 nm excitation and 490 nm emission, reading from the underneath the plate. Blank is defined as a non-reacted (non-glycosylated) region on the glass slide measured at the same time of the other readings with the same settings defined, all samples have been made relative to the blank region to determine significance. All samples reacted with 10 w/v% PEG, 5 w/v% AIBN in toluene for 1 hr at 100 ◦C with 1 mL deposited onto a slide (25 mm x 75 mm). Error bars are shown as one standard deviation between triplcate sample spots on the same slide.

Fluorescence data of Concanavalin A assay

Sample	Well			Average	Relative fluorescence	u_c	σ
	1	2	3				
Blank (non-glycosylated)	1140	878	1468	1162	0	241	273
D-ribose (**11**)	1797	1561	2197	1522	359	262	296
D-glucose (**6**)	2131	2861	3428	3203	2041	531	601
N-acetyl -D-glucoseamine (**9**)	1945	2368	2829	2513	1351	361	409
D-maltose (**12**)	3582	4460	3912	4223	3060	362	410
β-lactose (**14**)	4122	4830	5649	5296	4134	624	706
N-acetyl -D-lactoseamine (**13**)	5221	4784	3856	5390	4228	569	644
1,3-α-1,6-α-D-mannotriose (**15**)	8759	6844	8113	7825	6663	795	900
Acarbose (**16**)	5678	4408	4651	4912	3750	550	622
Maltoheptaose (**17**)	4973	5297	3548	4606	3444	760	860

Fluorescence was measured with 50 flashes per read of the wells analyzed with 485 nm excitation and 525 nm emission, reading from the underneath the plate. Blank is defined as a non-reacted (non-glycosylated) region on the glass slide measured at the same time of the other readings with the same settings defined, all samples have been made relative to the blank region to determine significance. The standard uncertainty (u_c ,k=1) and expanded uncertainty (σ, k=2) are given.
